# RNA sequencing of myeloid sarcoma, shed light on myeloid sarcoma stratification

**DOI:** 10.1002/cam4.5654

**Published:** 2023-03-14

**Authors:** Yunfan Yang, Yang Shu, Yuan Tang, Sha Zhao, Yongqian Jia, Jie Ji, Hongbing Ma, Ting Lin, Ke Zheng, Heng Xu, Yu Wu

**Affiliations:** ^1^ Department of Hematology, Institute of Hematology West China Hospital of Sichuan University Chengdu People's Republic of China; ^2^ Department of Gastrointestinal Surgery, State Key Laboratory of Biotherapy and Cancer Center West China Hospital of Sichuan University Chengdu People's Republic of China; ^3^ State Key Laboratory of Biotherapy and Cancer Center West China Hospital of Sichuan University Chengdu People's Republic of China; ^4^ Department of Pathology West China Hospital of Sichuan University Chengdu People's Republic of China; ^5^ Department of Laboratory Medicine West China Hospital of Sichuan University Chengdu People's Republic of China

**Keywords:** myeloid sarcoma, risk stratification, RNA sequencing

## Abstract

**Background:**

Myeloid sarcoma (MS) is a rare, extramedullary tumor consisting of myeloid blasts. Little is known about the genetic background of MS and the prognostic value of genetic abnormalities in MS. In particular, the broad variety of gene fusions that occur in MS is marginally covered by traditional testing methods due to lack of fresh tumor specimens.

**Methods:**

Here, we analyzed the clinical and genetic features of 61 MS cases. We performed RNA sequencing (RNA‐seq) on formalin‐fixed paraffin‐embedded (FFPE) or fresh samples to analyze fusion genes in 26 cases. In addition, we performed genetic abnormalities‐based risk stratification using fusion genes and gene mutations.

**Results:**

A total of 305 fusion genes were identified in 22 cases, including the following five recurrent fusion genes: *RUNX1‐RUNX1T1*, *CBFβ‐MYH11*, *ETV6‐MECOM*, *FUS‐ERG*, and *PICALM‐MLLT10*. The prognosis in the adverse‐risk group was significantly worse than that in the favorable/intermediate‐risk group (median survival: 12 months vs. not reached; *p* = 0.0004).

**Conclusion:**

These results indicated the efficacy of RNA‐seq using FFPE‐derived RNA as a clinical routine for detecting fusion genes, which can be used as markers for risk stratification in MS.

## Graphical abstract

We analyzed the clinical and genetic features of 61 MS cases. We performed genetic abnormalities based risk stratification based on gene mutations and fusion genes identified by RNA‐seq on FFPE. Our study suggested that RNA‐seq on FFPE could be as markers for MS risk stratification since fresh samples are usually unavailable.

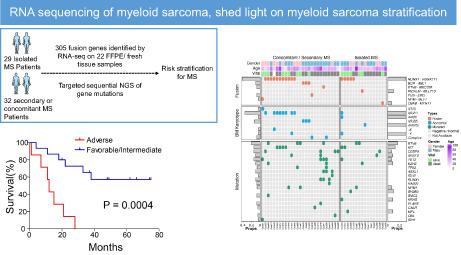

## 1 INTRODUCTION

Myeloid sarcoma (MS) is a rare hematological neoplasm characterized by the proliferation of myeloid blasts at extramedullary sites.^1^ The most common sites of MS presentation are the skin and soft tissue, lymph nodes, testis, bone, and mediastinum, although it can occur in almost any region in the body.^2^, ^3^, ^4^, ^5^, ^6^, ^7^, ^8^, ^9^, ^10^, ^11^, ^12^, ^13^, ^14^, ^15^ Isolated MS, which is defined as MS occurring before the bone marrow is infiltrated with blasts, may develop in about one‐fourth of all cases.^6^, ^7^ Other forms of MS develop concurrently with or secondary to acute myeloid leukemia (AML), chronic myeloid leukemia (CML), myeloproliferative neoplasm, or myelodysplastic syndrome (MDS).^2^, ^3^, ^4^, ^5^, ^6^, ^7^, ^9^, ^10^, ^14^, ^16^ As body scanning is not a standard radiologic survey for leukemia, the incidence of MS might be underestimated.^17^ Stolzel et al. found that positron emission tomography/computed tomography scans revealed extramedullary involvement in up to 22% patients with AML.^18^

Genetic changes are of fundamental importance in hematological neoplasms. The classification of AML was identified by cytogenetic and mutational profiles because of its overriding impact on disease phenotype and disease outcome.^19^ Furthermore, AML was classified into favorable, intermediate, and adverse‐risk groups depending on the chromosomes, gene mutations, and fusion genes involved. This risk stratification directly impacts the treatment strategy of AML, such as whether to perform allogeneic hematopoietic stem cell transplantation (allo‐HSCT). Multiple chromosomal anomalies, including *t*(8;21) (q22;q22), inv(16), 11q23, +8, −7, and complex cytogenetics, have been reported in MS.^4^, ^7^, ^15^, ^20^, ^21^, ^22^ Recurrent gene mutations in AML, such as *FLT3*‐internal tandem duplication (*FLT3‐*ITD), *NPM1*, and *NRAS*, have been reported in MS as well.^23^, ^24^, ^25^, ^26^, ^27^, ^28^, ^29^, ^30^ However, no comprehensive fusion gene analysis has been conducted in MS. This is because formalin‐fixed paraffin‐embedded (FFPE) tissue is almost always the only sample specimen available for isolated MS. Fusion assessment by polymerase chain reaction (PCR) requires RNA derived from fresh samples. Moreover, fluorescence in situ hybridization (FISH) can only be performed for the most common variants, although FISH can be conducted using FFPE samples. Therefore, performing extensive gene fusion screening of FFPE tissue is technically challenging. Because of the lack of information on fusion genes, we have been unable to perform genetic abnormalities‐based risk stratification for MS patients in clinical practice, as we do for AML.

In this study, we performed a clinical, cytogenetic, molecular, and prognostic analysis of a large series of MS cases. For the first time, we performed RNA sequencing (RNA‐seq) of FFPE samples to detect fusion genes in MS, and to preliminary analysis the prognostic value of these fusion genes. This study aimed to investigate a feasible way of deriving a prognosis and identifying therapeutically relevant molecular anomalies in MS.

## 2 MATERIALS AND METHODS

### 2.1 Patients and clinical information

This is an ambidirectional cohort study. Twenty‐two MS patients who were diagnosed in the West China Hospital of Sichuan University between October 2006 and May 2015 were retrospectively enrolled. Then, 39 cases between June 2015 and July 2020 were prospectively enrolled. All patients were diagnosed with MS on the basis of the tissue biopsy results and the World Health Organization criteria: MS is a tumor mass consisting of myeloid blasts and occurring at an anatomical site other than bone marrow. Infiltration of myeloid blasts into any site of the body in a patient with leukemia is not classified as MS unless it presents with tumor masses in which the tissue architecture is effaced.^1^ Patients with AML with only skin infiltration were excluded from this study because only skin infiltration did not present with “tumor masses,” and should be termed as “leukemia cutis.” Patient clinical information, including age; sex; MS presentation sites; and presence of previous, concomitant, or following hematopoietic/ nonhematopoietic tumors, was obtained from electronic medical records and telephone follow‐up. The survival status and date of death were obtained through the household registration system in China for patients who could not be reached by telephone. Treatment information was available in 57 patients. Among these, 47 (82.5%) received cytarabine‐containing chemotherapy, 11 (19.3%) surgical excision, 10 (17.6%) allo‐HSCT, eight (14.0%) local radiotherapy, seven (12.3%) target therapy (three tyrosine kinase inhibitor, two sorafenib and two venetoclax), and two (3.5%) palliative treatment. This study was carried out in accordance with the Helsinki Declaration and was approved by the ethics review committee of the West China Hospital (ethics committee approval number: 2020‐854). For the prospective aspect of the study, all the patients or their relatives provided written informed consent. Prior to the telephone follow‐up, patients or their relatives provided oral informed consent for the retrospective aspect of the study

### 2.2 Targeted testing for gene mutation and fusion genes

For MS patients secondary to or concomitant with leukemia or MDS, targeted gene mutation and fusion gene tests were performed using bone marrow as a clinical routine. Germline DNA was obtained from hair follicles or fingernails when specimens were available. High‐throughput sequencing of gene mutations was performed using a 15‐ or 34‐gene panel (Tables S1 and S2) on a MiSeq sequencer (Illumina Inc.). Fusion genes were tested using an 16‐fusion or 41‐gene panel (Tables S3 and S4) on multiplex reverse transcription PCR according to our institute's protocol.^31^ Specimens of interphase cells were examined using FISH, and 200 cells were examined for each probe used. FISH was performed using commercially available probes (Wuhan HealthCare Biotechnology) according to the manufacturers' protocols. The following probes were used: *RUNX1‐RUNX1T1* (dual color, dual fusion), *PICALM‐MLLT10* (dual color, dual fusion), and *FUS‐ERG* (dual color, dual fusion).

### 2.3 RNA‐seq

Total RNA was extracted and purified from FFPE samples or fresh MS samples using the RNeasy FFPE Kit (Qiagen) and TRIzol (Invitrogen Corporation), respectively. RNA integrity was assessed using the RNA Nano 6000 Assay Kit of the Bioanalyzer 2100 system (Agilent Technologies). The mRNA was purified from total RNA using poly‐T oligo‐attached magnetic beads and divided into short fragments using NEBNext First Strand Synthesis Reaction Buffer (5×; New England Biolabs). First strand cDNA was synthesized using a random hexamer primer and M‐MuLV Reverse Transcriptase. Second strand cDNA synthesis was subsequently performed using DNA polymerase I and RNase H. Remaining overhangs were converted into blunt ends via exonuclease/polymerase activities. After adenylation of 3′ ends of DNA fragments, adaptors with hairpin loop structures were ligated to prepare for hybridization. Then, PCR was performed using Phusion High‐Fidelity DNA polymerase, universal PCR primers, and an index (X) Primer. The PCR products were purified (AMPure XP system, Beckman Coulter Inc.), and library quality was assessed on the Agilent Bioanalyzer 2100 system. The clustering of the index‐coded samples was performed on a cBot Cluster Generation System using TruSeq PE Cluster Kit v3‐cBot‐HS (Illumina) according to the manufacturer's instructions. After cluster generation, the library preparations were sequenced on an Illumina Novaseq platform and 150‐bp paired‐end reads were generated.

### 2.4 Sequencing data processing

Sequencing data from tumor RNA samples were aligned to the GRCh37/hg19 human reference genome using the HISAT2 (v.2.2.0) alignment program.^32^ Subsequently, we performed duplicate reads removal, N Cigar reads splitting, InDel realignment, and base quality score recalibration with the Genome Analysis Toolkit (GATK, v.4.0.10.1)^33^ according to GATK best practices. The processed bam was subject to samtools (v.1.10)^34^ and varScan2 (v.2.4.4)^35^ for variant calling, where the interval regions were set as those of 42 genes in panel (Table S5). Furthermore, the variants were annotated with annovar software (v.2017‐07‐17).^36^ Meanwhile, gene expression values were quantified with the Subread bundle of featureCounts (v.1.5.3) according to the Gencode (v.19) annotation.^37^ To uncover the gene fusions, we used STAR‐Fusion (v.1.9.0)^38^ on fastq data, and the retained fusions with reads covering the junction or with annotation in databases were selected.

### 2.5 Statistical analysis

Overall survival (OS) was measured from the date of diagnosis of MS until death or date of the last follow‐up. The OS rates of different groups of MS were calculated by the Kaplan–Meier survival method and compared by the log‐rank test. All statistical tests were two‐sided, and *p*‐values below 0.05 were considered significant. All statistical analyses were performed using GraphPad Prism v.5.01 (GraphPad).

## 3 RESULTS

### 3.1 Patients and clinical information

Patients' age, sex, and development sites of MS are summarized in Table 1. Of the 61 patients included, 36 (59.0%) were male and 25 (41.0%) were female. The median age was 37 (range, 8–87) years. The most common sites of MS presentation were the lymph node (31.1%), soft tissue (24.6%), bones and joints (14.8%), mediastinum (11.5%), and central nervous system (9.8%).

**TABLE 1:** Patient characteristics: Age, sex, and development sites of MS in this study.

Patient characteristics	All	Isolated MS	Secondary/concomitant MS
No. case	*N* = 61	*N* = 29	*N* = 32
Median age (range)	37 (8–87)	35 (8–87)	37 (15–79)
Sex, *n* (%)			
Female	25 (41.0)	12 (41.4)	13 (40.6)
Male	36 (59.0)	17 (58.6)	19 (59.4)
Development sites, *n* (%)			
Lymph nodes (including Waldeyer's ring)	19 (31.1)	12 (41.4)	5 (15.6)
Soft tissue	15 (24.6)	4 (13.8)	10 (31.3)
Bones and joints	9 (14.8)	2 (6.9)	6 (18.8)
Mediastinum	7 (11.5)	5 (17.2)	2 (6.3)
Central nervous system	6 (9.8)	4 (13.8)	2 (6.3)
Orbit and eyelid	4 (6.6)	2 (6.9)	2 (6.3)
Pleural and abdominal cavity	4 (6.6)	2 (6.9)	2 (6.3)
Ovaries, uterus, and vagina	3 (4.9)	1 (3.4)	2 (6.3)
Breast	3 (4.9)	1 (3.4)	2 (6.3)
Oral cavity	2 (3.3)	2 (6.9)	0 (0.0)
Sinus and nasal cavity	2 (3.3)	1 (3.4)	1 (3.1)
Testis	2 (3.3)	0 (0.0)	2 (6.3)
Gastrointestine	2 (3.3)	2 (6.9)	0 (0.0)
Kidney	2 (3.3)	1 (3.4)	1 (3.1)
Liver	1 (1.6)	0 (0.0)	1 (3.1)

Twenty‐nine patients (47.5%) were diagnosed with isolated MS, of which 11 progressed to AML. The median time of progressing to AML was 8 (range, 2–42) months. Thirty‐two patients (52.5%) presented as secondary/concomitant MS as follows: Eight following AML (2 relapsed after allo‐HSCT), one following T lymphoblastic lymphoma, one following the blastic phase of CML (occurred after allo‐HSCT, concomitant diffuse large B cell lymphoma, and oral squamous cell carcinoma), 19 with concomitant AML (1 case with normal bone marrow morphology but *NPM‐MLF1* fusion gene‐positive that soon progressed to leukemia), one with CML, one with MDS, and one with primary myelofibrosis.

### 3.2 Cytogenetic and molecular findings of MS

**FIGURE 1:**
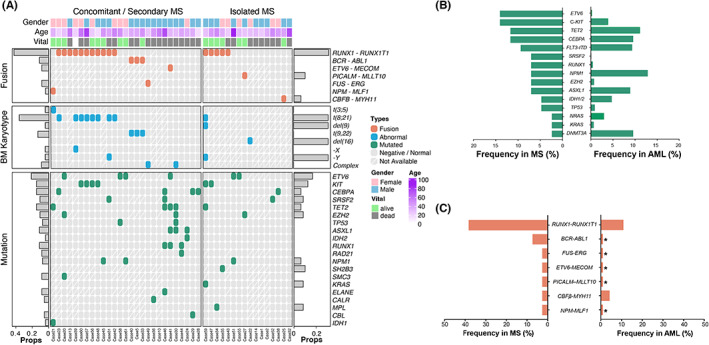
The cytogenetic and molecular profile of myeloid sarcoma (MS). (A) an overview of clinical and genetic information of 43 MS patients. (B) comparison of the gene mutation frequencies of MS in this study and Chinese patients with acute myeloid leukemia (AML)^36^. (C) comparison of the fusion genes frequencies of MS in this study and Chinese patients with AML^37^.

This study included patients over a period of 15 years. As a result, only a subset of patients had access to cytogenetic and molecular data. The cytogenetic analysis, fusion gene, and gene mutation results of evaluable MS patients are shown in Figure 1A and Table S6. In 25 patients with bone marrow involvement in AML, CML, or MDS, G‐band metaphase chromosome analysis was performed on the bone marrow samples. Of these, nine cases (36.0%) had *t*(8;21) (q22;q22), nine (36.0%) showed a normal karyotype, three (12.0%) had the Philadelphia chromosome, and two showed a complex karyotype.

Gene mutations were detected in 43 patients, with 29 (67.4%) harboring 55 gene mutations (Figure 1A and Table S6) and 13 (30.2%) with more than one mutation. Overall, the patients' mutational profiles showed frequent mutations in genes recurrently mutated in AML. However, the gene mutation frequencies in our series differed from those in Chinese patients with AML, as previously reported (Figure 1B).^39^ Mutations in *C‐kit* and *ETV6* were the most common in MS (6/43, 14.0%), followed by mutations in *TET2* (5/43, 11.6%), biallelic *CEBPA* (4/43, 9.3%), and *FLT3*‐ITD (4/43, 9.3%). Interestingly, all patients with a C‐*kit* mutation also possessed the *RUNX1‐RUNX1T1* fusion gene. Fusion gene test was performed in 42 patients by targeted next‐generation sequencing and/or RNA‐seq. Recurrent fusions in AML were detected in 57.1% (24/42) patients (Figure 1A and Table S6). *RUNX1‐RUNX1T1* was the most common gene fusion in MS (16/42, 38.1%), and its frequency in MS is much higher than that in the Chinese AML study (38.1% vs. 10.7%) (Figure 1C).^40^ Taking gene mutations and fusions together, 51.2% (22/43) of MS patients harbored genetic anomalies associated with transcription factor genes (*RUNX1* and *ETV6*).

### 3.3 RNA‐Seq of FFPE‐derived RNA can identify gene fusions in MS

**FIGURE 2:**
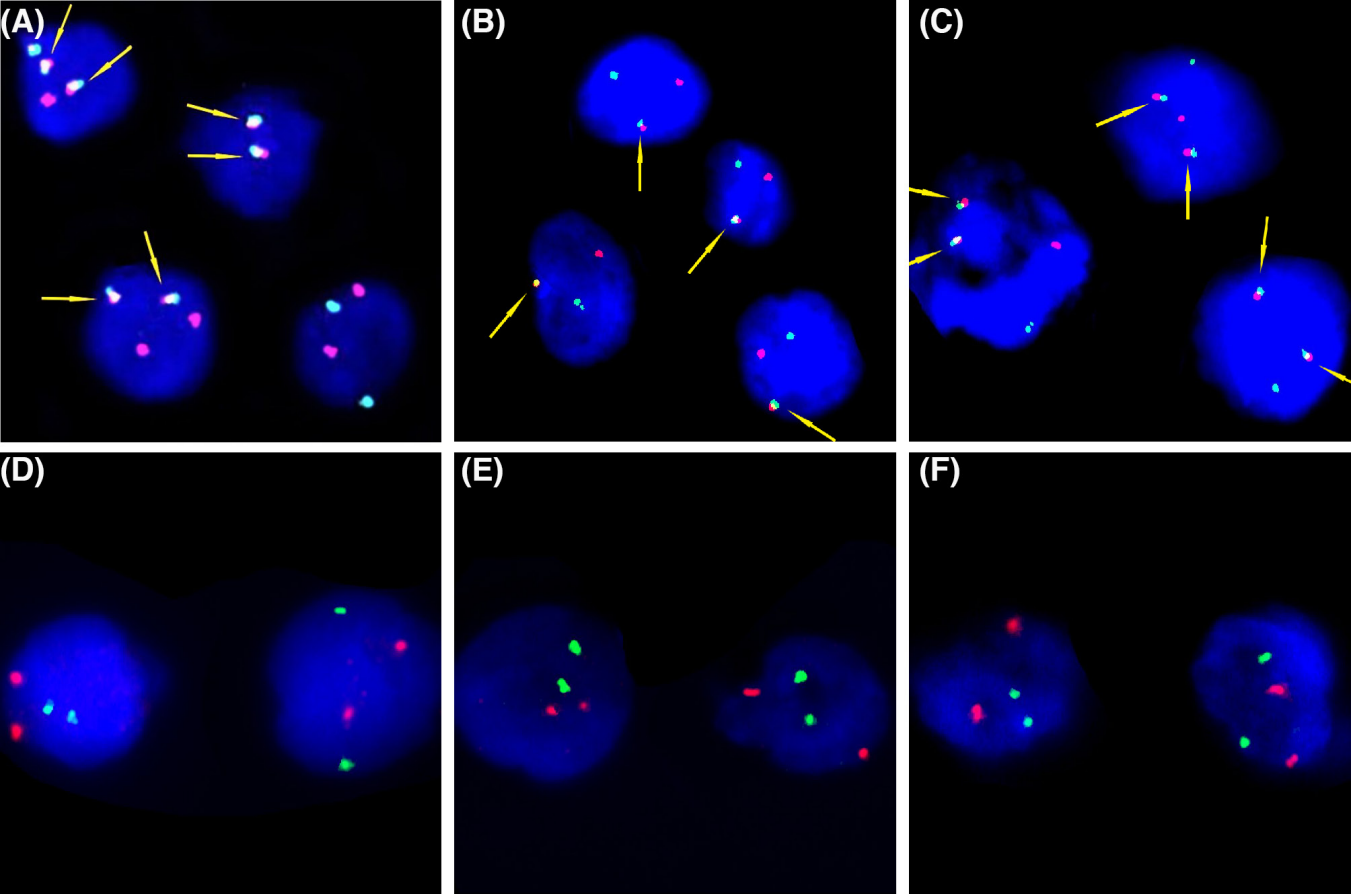
FISH validation of fusion genes detected by RNA sequencing (RNA‐seq). Interphase FISH analysis of the *RUNX1‐RUNX1T1* (A), *PICALM‐MLLT10* (B), and *FUS‐EGR* (C) positive cases; arrow: gene fusion (one red/green fusion signal). Interphase FISH analysis of the *RUNX1‐RUNX1T1* (D), *PICALM‐MLLT10* (E), and *FUS‐EGR* (F) negative cases.

We performed RNA‐seq in 26 patients. Sequencing library preparation for four FFPE samples failed due to RNA degradation or DNA contamination. The other 22 samples included 16 FFPE samples and six fresh tissue samples. A total of 305 fusion genes were identified in 22 patients (Figure S1 and Table S7). Five identified fusions were previously reported as recurrent fusion genes in AML, including the common fusion genes *RUNX1‐RUNX1T1* and *CBFβ‐MYH11*, as well as the rare fusion genes *ETV6‐MECOM*, *FUS‐ERG*, and *PICALM‐MLLT10*.^19^ In eight patients with concomitant/secondary MS, RNA‐seq of MS tissue and bone marrow were performed, and the recurrent fusions tested in MS tissue and bone marrow were concordant in all the eight patients. We validated the gene fusions tested by RNA‐seq of FFPE‐derived RNA by FISH in five patients, including three patients with the *RUNX1‐RUNX1T1* fusion gene (cases 20, 45, and 47), one patient with *PICALM‐MLLT10* (case 27), and one patient with *FUS‐ERG* (case 49) (Figure 2). All FISH tests confirmed the fusion gene results tests by RNA‐seq. These results proved that RNA‐seq of FFPE‐derived RNA reliably identified known fusion genes in MS, which provide essential information for MS risk stratification.

### 3.4 Gene mutations and fusion genes could be used for risk stratification in MS

**FIGURE 3:**
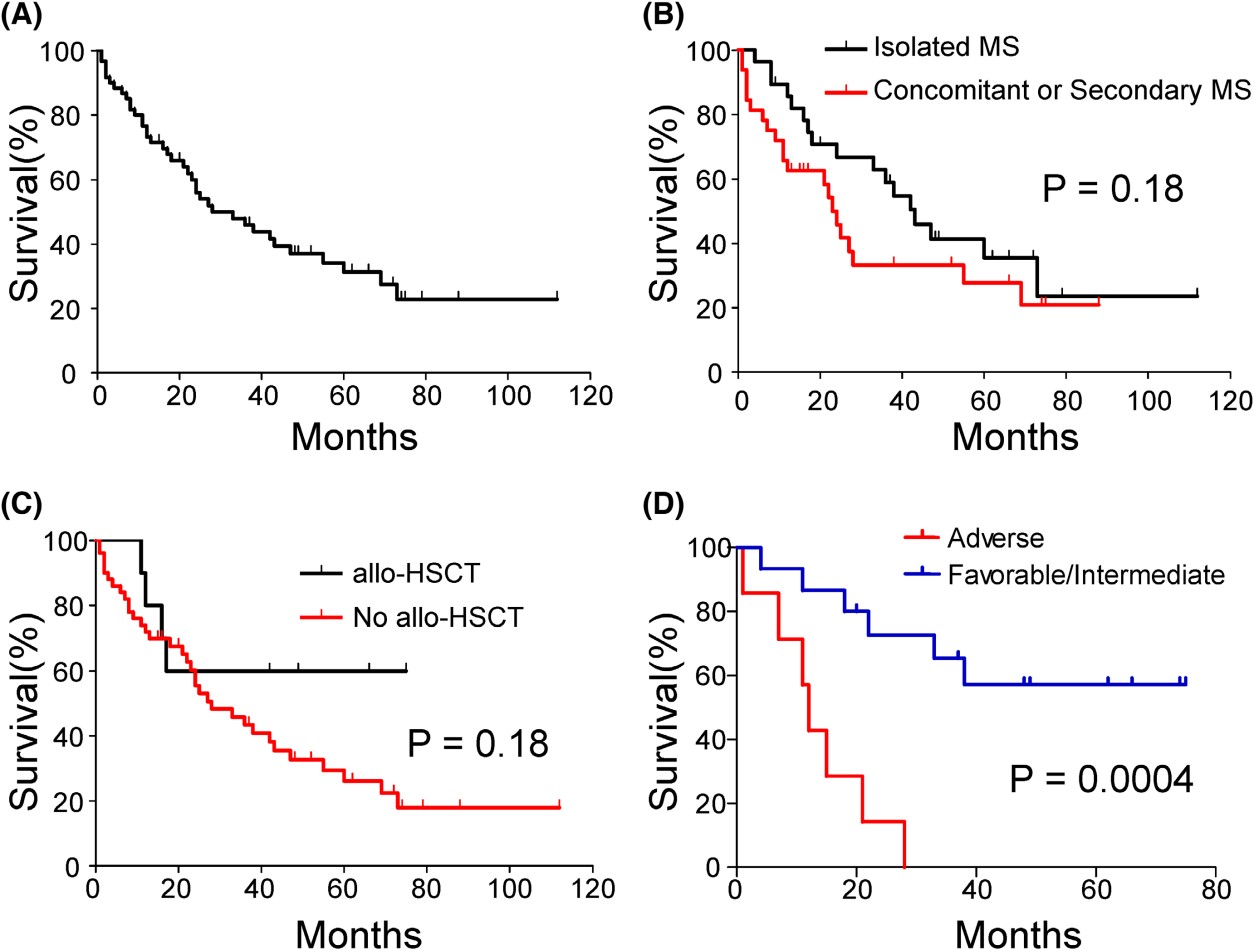
Survival analysis of MS patients. (A) overall survival (OS) curve of 60 MS patients. (B) Kaplan–Meier plot comparing the OS between isolated MS and concomitant/secondary MS (C) Kaplan–Meier plot evaluating the impact of allogeneic hematopoietic stem cell transplantation on OS of MS. (D) Kaplan–Meier plot comparing the OS between adverse‐risk group and the favorable or intermediate‐risk group of 22 patients with RNA‐seq information. Risk groups were divided based on the 2017 European Leukemia Network risk stratification for AML^17^ according to gene fusions and mutations of MS.

Follow‐up data were available on 60 patients. At a median follow‐up of 66 months, 38 (63.3%) patients died of disease. The OS data show strong heterogeneity in the prognosis of MS patients. The median OS was 28 (range, 1–112) months (Figure 3A). The OS curves of isolated MS (*n* = 28) and concomitant or secondary MS (*n* = 32) are shown in Figure 3B. The median survival of isolated MS and concomitant/secondary MS were 43 and 23.5 months, respectively. Isolated MS tended to have longer OS, although not statistically significant (p = 0.18). Ten patients received allo‐HSCT, and the median survival was longer than that of those who did not receive allo‐HSCT (not reached vs. 28 months) (Figure 3C), although not statistically significant (*p* = 0.18). To investigate whether genetic information can predict the prognosis of MS as it can in AML, we divided patients into different risk groups according to their genetic anomalies. The cytogenetic information was replaced by fusion genes identified by RNA‐seq. Based on the 2017 European Leukemia Network risk stratification for AML,^19^ 22 patients with RNA‐seq information were divided into the adverse‐risk group and the favorable or intermediate‐risk group according to gene fusions and mutations; seven patients were included in the adverse‐risk group, whereas the other 15 fell under the favorable/intermediate group. The median survival of the adverse‐risk group was significantly shorter than that of the favorable/intermediate‐risk group (12 months vs. not reached, *p* = 0.0004) (Figure 3D).

**FIGURE 4:**
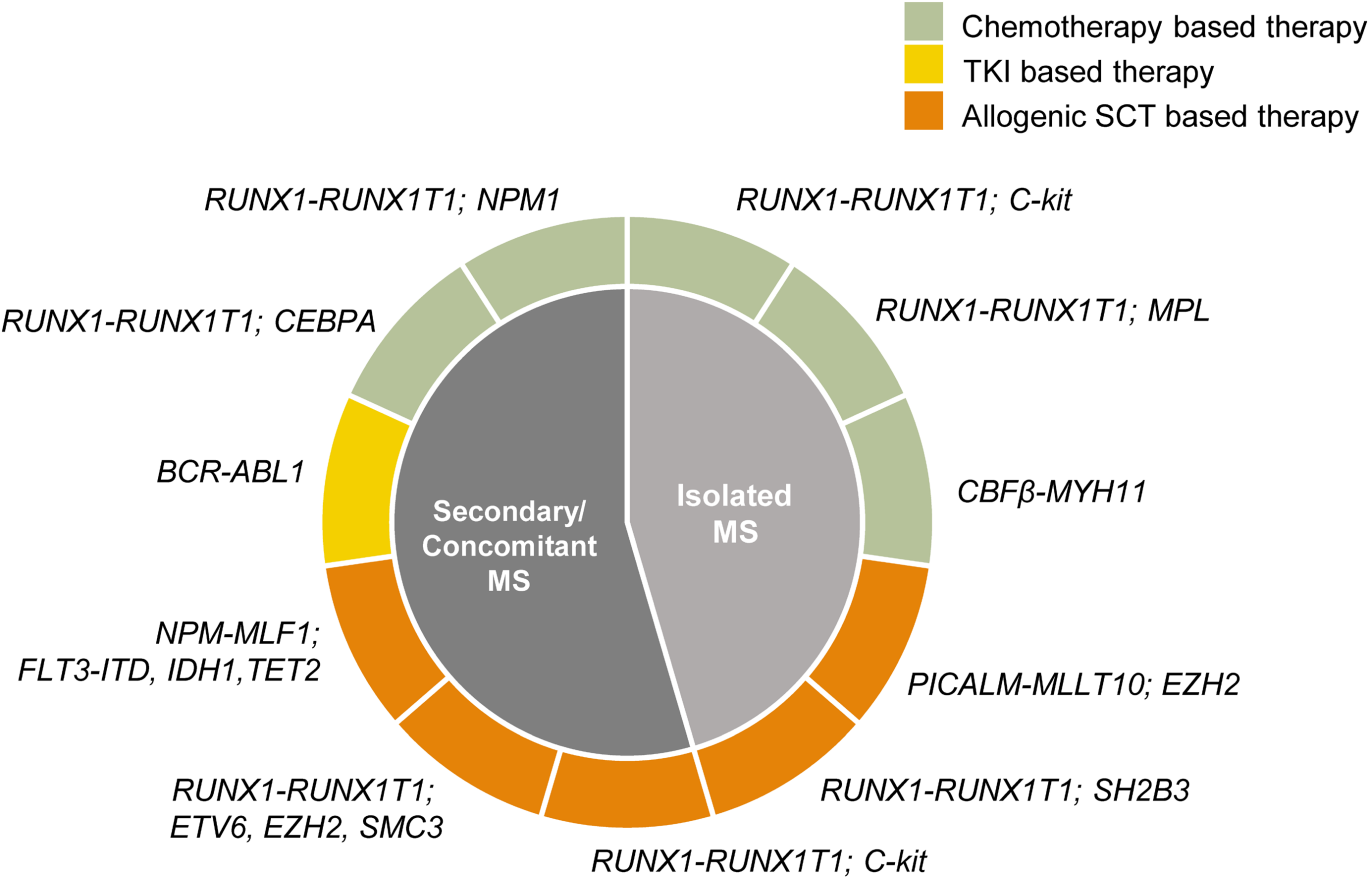
Clinical and genetic information of 11 MS patients achieved long‐term survival. Long‐term survival was defined as OS >36 months and achieving complete remission until the last follow‐up.

Fifteen MS patients achieved long‐term survival, defined as OS >36 months and achieving complete remission until the last follow‐up. The median follow‐up of these patients was 66 (range, 37–112) months. Only 6 of 15 cases underwent allo‐HSCT. Seven cases received chemotherapy‐based treatment, one received surgery and radiotherapy, and one received only nilotinib. Details on the molecular genetics were only available for 11 patients (Figure 4). All long‐term survival patients treated only with chemotherapy harbored favorable‐prognosis mutations or fusions (*NPM1*, biallelic *CEBPA*, and *RUNX1‐RUNX1T1*). Interestingly, case 25, an isolated MS patient with the fusion *CBFβ‐MYH11* who underwent tumor resection and only two chemotherapy cycles, achieved long‐term survival at a follow‐up of 57 months. In contrast, only two patients with adverse fusion or mutation achieved long‐term survival. One patient with *BCR‐ABL1* received nilotinib therapy, whereas another received allo‐HSCT.

## 4 DISCUSSION

MS is a disease that often poses therapeutic dilemmas. In contrast to AML, the significance of molecular abnormalities in MS is not fully established, particularly in isolated MS.^21^ The clinically feasible detection methods of fusion genes and the prognostic significance of fusion genes are especially the missing links. In clinical practice, it is still difficult to integrate AML risk stratification into the treatment of myeloid sarcoma. In this study, we performed a comprehensive analysis of 61 MS cases with clinical, pathologic, and genetic information. For the first time, we demonstrated that RNA‐seq from FFPE‐derived RNA is a reliable method for detecting fusion genes in MS. Furthermore, these fusion genes, together with gene mutations, can be used as markers for MS risk stratification.

The current World Health Organization classification of AML has introduced many fusion genes as molecular markers. The fusions in MS, especially in isolated MS, have rarely been systematically investigated due to a lack of fresh samples. Our results showed that 57.1% of MS patients had recurrent fusion genes, a significantly higher rate than that in AML (21%, other than acute promyelocytic leukemia).^19^ Furthermore, up to 38.1% of MS patients have the *RUNX1‐RUNX1T1* fusion gene, which is higher than 10.7% in AML.^40^ Many previous studies report that MS is closely related to t(8;21) or *RUNX1‐RUNX1T1*.^41^, ^42^, ^43^, ^44^ The underlying mechanism of t(8;21) contributing to MS remains to be elucidated. A recent study demonstrated that a subgroup of AML patients with t(8;21) highly expressed genes associated with cell migration and adhesion (*LGALS1*, *EMP3*, and *ANXA2*).^45^ Comparing the gene profile of AML patients harboring t(8;21) with or without MS would yield interesting findings. Dalland et al.^46^ described 11 MS cases with *CBFβ‐MYH11* fusion and reviewed another 22 in the literature. The authors found that 94% (31/33) of the MS cases with *CBFβ‐MYH11* fusion involved abdominal sites. Thus, MS with *CBFβ‐MYH11* fusion may represent a unique phenomenon. In our study, case 25 showed MS in the small intestine with *CBFβ‐MYH11* fusion, and achieved long‐term survival while undergoing only two cycles of chemotherapy. The correlation between fusion genes and the MS phenotype suggests the potential value of fusion genes as molecular markers for the classification of MS. In addition, we have identified several rare but recurrent gene fusions through RNA‐seq. *FUS‐ERG* and *PICALM‐MLLT10* have been described previously in case reports.^47^, ^48^ *ETV6‐MECOM* and *NPM‐MLF1*, which are recurrent fusion genes in AML,^49^, ^50^ were first reported in MS. Our data broaden current knowledge on fusion genes in MS.

MS is often diagnosed incidentally by surgery or biopsy. When the diagnosis is confirmed, performing an additional biopsy to obtain a fresh sample is unlikely. Thus, FFPE samples are often the only available specimen source. This prevents the use of PCR on detecting fusion genes of isolated MS. Pileri et al.^14^ demonstrated FISH was a reliable way to detect known fusion genes of isolated MS. Similar to AML stratification, identification of known fusion genes is in priority, we suggest FISH should first be performed to detect *RUNX1‐RUNX1T1* fusion since it occurs at high proportion in MS patients and is mutually exclusive with other fusion genes. However, the wide variety of chromosomal anomalies in MS makes FISH for other fusion genes uneconomical and time‐consuming, given the need to prepare rare probes in clinical routine. RNA‐seq has become a ubiquitous tool for detecting fusion genes in many cancers.^51^ Our results suggest that RNA‐seq is reliable in detecting fusion genes in FFPE samples of MS patients, with a wide range of detection capabilities and a high degree of consistency with FISH. In our experience, FFPE samples collected within 6 months are suitable for RNA‐seq. Otherwise, a longer storage time will lead to excessive degradation of RNA and cause failure in sequence library preparation.

**FIGURE 5:**
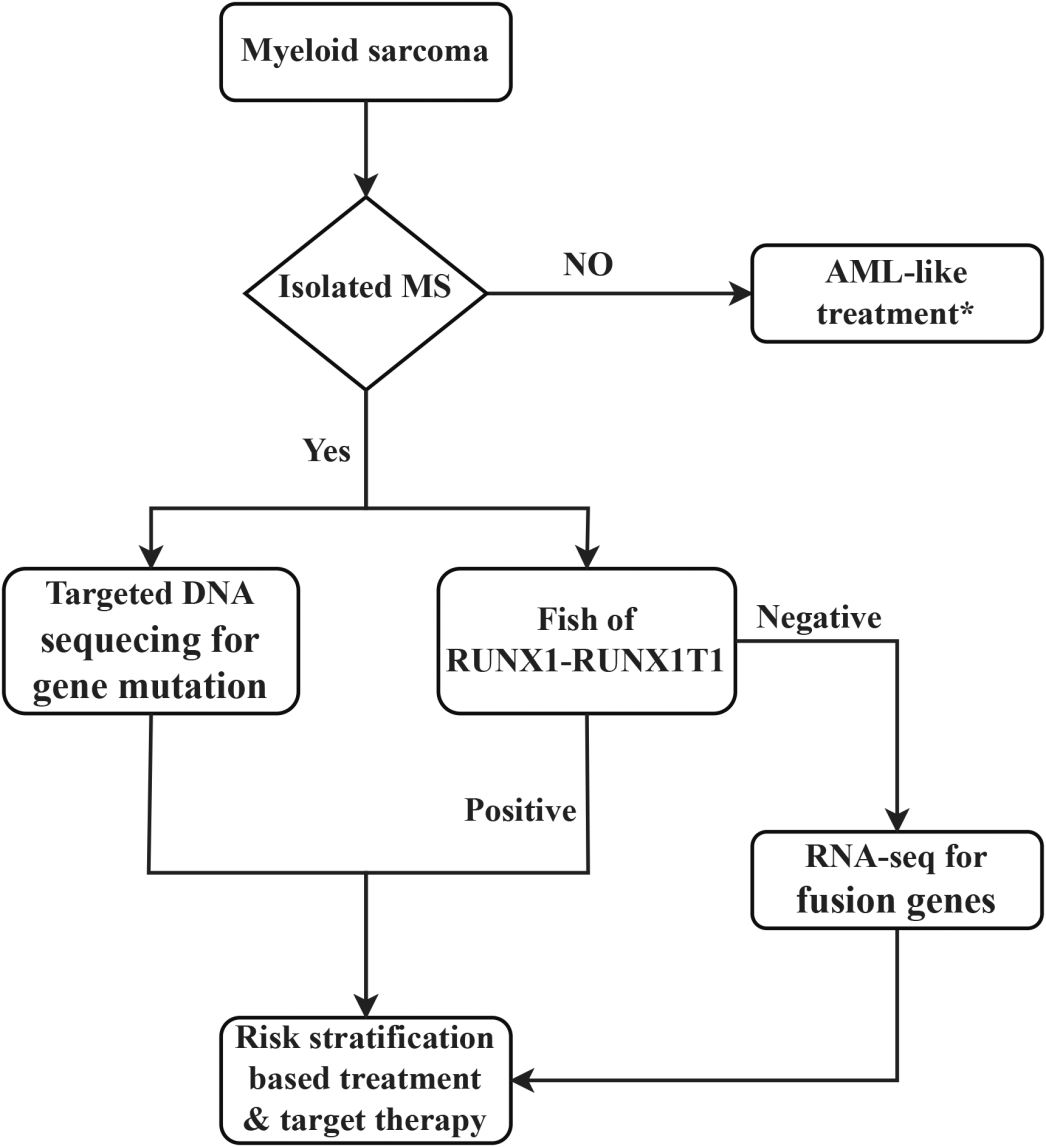
Our diagnosis and treatment algorithm for MS. *AML‐like treatment means systemic therapy, combined with local therapy (radiation therapy or surgery) when necessary.

Like in AML, testing for gene mutations in MS has both diagnostic and therapeutic implications. Previous studies have demonstrated that MS harbors gene mutations that frequently occur in AML, MDS, and myeloproliferative neoplasm.^23^, ^24^, ^25^, ^26^, ^27^, ^28^, ^29^ Together with high‐frequency mutations, we identified rare mutations, including those in *SMC3*, *RAD21*, and *SH2B3*, which were also found in other myeloid neoplasms.^19^, ^52^ These findings support the assumption that MS is a complex, dynamic disease. Therefore, the combination of FISH, RNA‐seq, and targeted DNA sequencing may be a suitable detection procedure in MS patients. Our recommendation on the diagnostic flow of MS is shown in Figure 5.

Data on MS prognosis are conflicting and limited. Several studies have suggested the site of involvement, background disease, and immunohistochemical expression of CXCR4 to be closely related to the prognosis of MS.^5^, ^6^, ^7^, ^10^ A retrospective study showed that an abnormal karyotype was associated with poor prognosis.^22^ The use of gene mutations and fusion genes in MS for risk stratification and treatment planning, as in AML, is a critical question. In this study, we tried to classify MS by using the 2017 European Leukemia Network risk stratification for AML. We found a significant difference in OS between the adverse and favorable/intermediate‐risk groups (*p* = 0.0004), which indicates the possibility of establishing an AML‐like risk stratification system for MS using gene mutations and fusion genes.

Until now, no consensus has been reached on the standard treatment for MS because of the lack of prospective studies.^53^ Some studies indicated HSCT can improve the prognosis of MS.^14^, ^54^ Pileri et al.^14^ analyzed 67 MS patients and found that 6 out of 7 patients who were still alive at the end of follow‐up underwent allo‐HSCT. Thus, they suggested that allo‐HSCT could be the only treatment for MS. In this study, we described 15 MS cases who achieved long‐term survival. Only six of them underwent allo‐HSCT. Moreover, all long‐term survival cases without allo‐HSCT harbored favorable‐prognosis molecular features or a targetable fusion gene. Again, this finding indicates the potential use of gene mutations and fusions as prognostic markers and in determining the most appropriate treatment in MS. In summary, our data suggest that MS is a disease with a very heterogeneous prognosis that does not necessarily require one‐size‐fits‐all allo‐HSCT. The type of MS and prognostic fusions and mutations are helpful in risk stratification‐based therapy.

Owing to the rarity of MS, prospective trials on MS treatment and prognosis are few. Our study had several limitations. First, this was a single‐center, ambidirectional observational study with inclusion bias. Second, this study was spanned over decades, due to which batch to batch experimental variation could not be avoided. Third, MS survival is longer than AML survival, requiring long‐term observation. Further, treatment modality bias existed in our study, especially after the initiation of our study. Biological information of MS diagnosed in recent years is more comprehensive and precise than that available for cases diagnosed previously.

This is the first comprehensive analysis of MS cases using RNA‐seq. We have shown that RNA‐seq can be routinely applied with diagnostic FFPE samples of MS patients. Future sue prospective studies are needed to establish a prognostic stratification system incorporating clinical characteristics and genetic information and to provide conclusive findings that can guide the stratified and precise treatment of MS.

## AUTHOR CONTRIBUTIONS

**Yunfan Yang:** Conceptualization (equal); data curation (lead); formal analysis (lead); funding acquisition (supporting); methodology (equal); project administration (equal); software (equal); supervision (equal); validation (equal); visualization (supporting); writing – original draft (lead); writing – review and editing (lead). **Yang Shu:** Conceptualization (equal); data curation (equal); formal analysis (equal); investigation (equal); methodology (equal); software (lead); supervision (equal); validation (equal); visualization (lead); writing – original draft (equal); writing – review and editing (equal). **Yuan Tang:** Conceptualization (supporting); data curation (supporting); funding acquisition (supporting); investigation (supporting); methodology (equal); resources (lead); writing – review and editing (supporting). **Sha Zhao:** Data curation (supporting); funding acquisition (supporting); investigation (equal); methodology (equal); resources (equal); writing – review and editing (equal). **Yongqian Jia:** Data curation (supporting); resources (equal); writing – review and editing (equal). **Jie Ji:** Data curation (equal); resources (supporting); writing – review and editing (equal). **Hongbing Ma:** Data curation (supporting); resources (supporting); writing – review and editing (supporting). **Ting Lin:** Data curation (equal); software (supporting); writing – original draft (supporting); writing – review and editing (equal). **Ke Zheng:** Data curation (supporting); methodology (supporting); writing – review and editing (supporting). **Heng Xu:** Conceptualization (equal); data curation (equal); formal analysis (equal); funding acquisition (equal); investigation (equal); methodology (equal); software (equal); validation (equal); visualization (equal); writing – original draft (supporting); writing – review and editing (equal). **Yu Wu:** Conceptualization (lead); data curation (equal); formal analysis (equal); funding acquisition (lead); investigation (equal); methodology (equal); project administration (lead); resources (equal); supervision (equal); validation (lead); writing – original draft (equal); writing – review and editing (lead).

## Acknowledgments

We would like to thank the patients for participating in this study.

## FUNDING INFORMATION

This project was supported by the “1·3·5 project for disciplines of excellence–Clinical Research Incubation Project, West China Hospital, Sichuan University.”

## CONFLICT OF INTEREST STATEMENT

The authors declare that they have no competing interests.

## ETHICAL APPROVAL STATEMENT

This study was conducted in accordance with the Helsinki Declaration and approved by the ethics review committee of the West China Hospital.

## Supporting information


Appendix S1.

## Data Availability

The datasets analyzed during the current study are available on https://bigd.big.ac.cn/gsa‐human/. The data number is PRJCA008485.
