# Supplementary material for: RNA sequencing of myeloid sarcoma, shed light on myeloid sarcoma stratification
Source: Cancer Med. 2023 Mar 14;12(8):9156–66. doi: 10.1002/cam4.5654 (PMC10166975; doi:10.1002/cam4.5654)
Supplement: Supplementary file 1 — Appendix S1. [file CAM4-12-9156-s001.docx]

| Table S1: 15 gene muation panel for targeted test | | |
| --- | --- | --- |
| Gene | Gen Bank Accession Number | Exons |
| *ASXL1* | NM_015338 | 13 |
| *CEBPA* | NM_004364 | 1 |
| *DNMT3A* | NM_022552 | 2–23 |
| *EZH2* | NM_004456 | 2-20 |
| *FLT3* | NM_004119 | 14, 15, 20 |
| *IDH1* | NM_005896 | 4 |
| *IDH2* | NM_002168 | 4 |
| *KIT* | NM_000222 | 8-11, 13, 17 |
| *NPM1* | NM_002520 | 11 |
| *NRAS* | NM_002524 | 2, 3 |
| *PHF6* | NM_001015877 | 2-10 |
| *RUNX1* | NM_001001890 | 2-9 |
| *TET2* | NM_001127208 | 3–11 |
| *TP53* | NM_000546 | 4–9 |
| *WT1* | NM_024426 | 1–10 |

| Table S2: 34 gene muation panel for targeted test | | |
| --- | --- | --- |
| Gene | Gen Bank Accession Number | Exons |
| *ASXL1* | NM_015338 | 13 |
| *BCOR* | NM_001123385 | 2-15 |
| *BCORL1* | NM_021946 | 1-12 |
| *CALR* | NM_004343 | 9 |
| *CBL* | NM_005188 | 8, 9 |
| *CEBPA* | NM_004364 | 1 |
| *CSF3R* | NM_000760 | 14-17 |
| *DNMT3A* | NM_022552 | 2–23 |
| *ETV6* | NM_001987 | 3–8 |
| *ETNK1* | NM_018638 | 3 |
| *EZH2* | NM_004456 | 2-20 |
| *FLT3* | NM_004119 | 14, 15, 20 |
| *IDH1* | NM_005896 | 4 |
| *IDH2* | NM_002168 | 4 |
| *JAK2* | NM_004972 | 12-16, 20, 21 |
| *KIT* | NM_000222 | 8-11, 13, 17 |
| *KRAS* | NM_033360 | 2, 3 |
| *MPL* | NM_005373 | 10 |
| *NF1* | NM_001042492 | 1-49 |
| *NPM1* | NM_002520 | 11 |
| *NRAS* | NM_002524 | 2, 3 |
| *PHF6* | NM_001015877 | 2-10 |
| *PIGA* | NM_002641 | 2-6 |
| *PTPN11* | NM_002834 | 3, 4, 12, 13 |
| *RUNX1* | NM_001001890 | 2-9 |
| *SETBP1* | NM_015559 | 4 |
| *SF3B1* | NM_012433 | 12-15 |
| *SRSF2* | NM_003016 | 1 |
| *STAG2* | NM_001042750 | 2-33 |
| *TET2* | NM_001127208 | 3–11 |
| *TP53* | NM_000546 | 4–9 |
| *U2AF1* | NM_001025203 | 2, 6, 8 |
| *WT1* | NM_024426 | 1–10 |
| *ZRSR2* | NM_005089 | 1–11 |

| Table S3: 16 fusion gene panel for targeted test |
| --- |
| fusion genes |
| *BCR-ABL1*( p190) |
| *BCR-ABL1* (p210) |
| *CBFβ-MYH11* |
| *DEK-CAN* |
| *dupMLL* |
| *MLL-AF10* |
| *MLL-AF17* |
| *MLL-AF6* |
| *MLL-AF9* |
| *MLL-ELL* |
| *NPM-MLF1* |
| *NPM-RARα* |
| *PLZF-RARα* |
| *PML-RARα* |
| *RUNX1-RUNX1T1* |
| *TLS-ERG* |

| Table S4: 41 fusion gene panel for targeted test |
| --- |
| fusion genes |
| *BCR-ABL1* |
| *CBFβ-MYH11* |
| *DEK-CAN* |
| *dupMLL* |
| *ETV6-PDGFRα* |
| *ETV6-PDGFRβ* |
| *ETV6-RUNX1* |
| *FIPIL1-PDGFRα* |
| *FIPIL1-RARα* |
| *MLL-AF10* |
| *MLL-AF17* |
| *MLL-AF1P* |
| *MLL-AF1Q* |
| *MLL-AF4* |
| *MLL-AF6* |
| *MLL-AF9* |
| *MLL-AFX* |
| *MLL-ELL* |
| *MLL-ENL* |
| *NPM-ALK* |
| *NPM-MLF1* |
| *NPM-RARα* |
| *NuMA1-RARα* |
| *NuP98-HOxA11* |
| *NuP98-HOxA13* |
| *NuP98-HOxA9* |
| *NuP98-HOxC11* |
| *NuP98-HOxD13* |
| *NuP98-PMX1* |
| *PLZF-RARα* |
| *PML-RARα* |
| *PRKARIA-RARα* |
| *RUNX1-RUNX1T1* |
| *RUNX1-MDS1* |
| *SET-CAN* |
| *SIL-TAL1* |
| *STAT5b-RARα* |
| *TCF3-HLF* |
| *TCF3-PBX1* |
| *TEL-ABL1* |
| *TLS-ERG* |

| Table S5: Targeted Genes Interrogated by OncoHeme Next-Generation Sequencing * | | |
| --- | --- | --- |
| Gene | Gen Bank Accession Number | Exons |
| *ANKRD26* | NM_014915.2 | 1–4, 5’UTRc.-172 |
| *ASXL1* | NM_015338.5 | 10–13 |
| *BCOR* | NM_001123385.1 | 4–15 |
| *CALR* | NM_004343.3 | 9 |
| *CBL* | NM_005188.3 | 8–9, intron 7 100bp before exon 8 and intron 8 |
| *CEBPA* | NM_004364.4 | 1 |
| *CSF3R* | NM_000760.3 | 14 and 17 |
| *DDX41* | NM_016222.2 | 1–17 |
| *DNMT3A* | NM_022552.4 | 8–23 |
| *ELANE* | NM_001972.2 | 1–5 |
| *ETNK1* | NM_018638.4 | 2–5 |
| *ETV6* | NM_001987.4 | 3–8 |
| *EZH2* | NM_004456.4 | 2–20 |
| *FLT3* | NM_004119.2 | 14–20 |
| *GATA1* | NM_002049.3 | 2 and 4 |
| *GATA2* | NM_001145661.1 | 3–7, intron5 c.1017+1 to 1017+730 |
| *IDH1* | NM_005896.3 | 4 |
| *IDH2* | NM_002168.3 | 4 |
| *JAK2* | NM_004972.3 | 12–16 |
| *KDM6A(UTX)* | NM_021140.3 | 1–29 |
| *KIT* | NM_000222.2 | 8–11 and 17 |
| *KRAS* | NM_033360.3 | 2–3 |
| *MPL* | NM_005373.2 | 10–12 |
| *NPM1* | NM_002520.6 | 9–11, intron 1030 bp before exon 11 |
| *NRAS* | NM_002524.4 | 2 and 3 |
| *PHF6* | NM_001015877.1 | 2–10 |
| *PTPN11* | NM_002834.3 | 3–4 and 12–13 |
| *RAD21* | NM_006265.2 | 1, 2, 4–7, 9–11, 13, 14 |
| *RUNX1* | NM_001001890.2 | 1–6, intron 4 c.725–13 T>A and intron 5 c.886+1-4del |
| *SETBP1* | NM_015559.2 | partial exon 4; aminon acids 400-950 |
| *SH2B3(LNK)* | NM_005475.2 | 2–8 |
| *SF3B1* | NM_012433.2 | 13–16 |
| *SRP72* | NM_006947.3 | 6, 10 |
| *SMC3* | NM_005445.3 | 7, 8, 13, 17, 19, 21, 29 |
| *SRSF2* | NM_003016.4 | 1 and 2 |
| *STAG2* | NM_001042750.1 | 4–34 |
| *TERT* | NM_198253.2 | 2–16 |
| *TET2* | NM_001127208.2 | 3–11 |
| *TP53* | NM_000546.4 | 4–9 |
| *U2AF1* | NM_001025203.1 | 2, 6, 8 |
| *WT1* | NM_024426.2 | 1–10 |
| *ZRSR2* | NM_005089.3 | 1–11 |
| * This panel can be found in https://www.mayocliniclabs.com/~/media/it-mmfiles/special-instructions/Targeted_Genes_Interrogated_by_OncoHeme_Next-Generation_Sequencing.pdf | | |

| Table S6: Genetic information of 43 MS patients | | | | | |
| --- | --- | --- | --- | --- | --- |
| Case number | Gene mutations | Fusion genes | Chromosome karyotype | Sample type | Detection method for mutations and fusions |
| 1 | Neg | Neg | NA | BM | Targeted NGS and PCR |
| 2 | Neg | Neg | NA | BM | Targeted NGS and PCR |
| 5 | Neg | *BCR-ABL1* | 46,XY,t(9;22)(q34;q11)[20] | BM | Targeted NGS and PCR |
| 10 | *FLT3*-ITD | Neg | NA | BM | Targeted NGS and PCR |
| 13 | Neg | *RUNX1-RUNX1T1* | NA | BM | Targeted NGS and PCR |
| 14 | Neg | Neg | NA | BM | Targeted NGS and PCR |
| 20 | *ETV6, EZH2, SMC3* | *RUNX1-RUNX1T1* | 46,XX[20] | BM & FFPE | Targeted NGS, PCR and RNA-seq |
| 21 | *FLT3-ITD, IDH1, TET2* | *NPM-MLF1* | 47,XX,t(3;5)(q25;q34),+4[18]/46,xx[2] | BM | Targeted NGS and PCR |
| 22 | Neg | Neg | 46,XX,del(16)(q22)[14]/46,xx[6] | BM | Targeted NGS and PCR |
| 23 | *CEBPA* | *RUNX1-RUNX1T1* | 46,XX,t(8;21)(q22;q22)[20] | BM & FFPE | Targeted NGS, PCR and RNA-seq |
| 24 | *FLT3-*ITD*, ASXL1, IDH2* | Neg | 46,XX[20] | BM & Fresh MS tissue | Targeted NGS, PCR and RNA-seq |
| 25 | Neg | *CBFβ-MYH11* | NA | FFPE | RNA-seq |
| 27 | *EZH2* | *PICALM–MLLT10* | 46,XY[20] | FFPE | RNA-seq |
| 28 | *FlT3-*ITD*, CEBPA* | Neg | 46,XY[20] | BM & Fresh MS tissue | Targeted NGS, PCR and RNA-seq |
| 29 | *CEBPA, CBL* | Neg | 46,XY[20] | BM & Fresh MS tissue | Targeted NGS, PCR and RNA-seq |
| 30 | *ASXL1, TET2, EZH2, TP53* | Neg | Complex karyotype | BM & FFPE | Targeted NGS, PCR and RNA-seq |
| 31 | *SRSF2* | *RUNX1-RUNX1T1* | 45,X,-Y,t(8;21)(q22;q22)[20] | BM & Fresh MS tissue | Targeted NGS and PCR |
| 32 | Neg | Neg | NA | FFPE | RNA-seq |
| 33 | Neg | *BCR-ABL1* | 46,XY,t(9;22)(q34;q11)[20] | BM | Targeted NGS and PCR |
| 34 | *MPL* | *RUNX1-RUNX1T1* | NA | FFPE | RNA-seq |
| 35 | *SH2B3* | *RUNX1-RUNX1T1* | NA | BM & FFPE | Targeted NGS, PCR and RNA-seq |
| 36 | *CALR* | NA | NA | BM | Targeted NGS and PCR |
| 38 | *CEBPA* | Neg | NA | FFPE | RNA-seq |
| 39 | Neg | *RUNX1-RUNX1T1* | 45,x,-X,t(8;21)(q22;q22)[3]/46,XX[4] | BM | Targeted NGS and PCR |
| 40 | Neg | *BCR-ABL1* | 46,XY,t(9;22)(q34;q11)[20] | BM | Targeted NGS and PCR |
| 41 | *ETV6, ASXL1, RUNX1, TET2,* | *ETV6-MECOM* | 46,XY[20] | BM & Fresh MS tissue | Targeted NGS, PCR and RNA-seq |
| 42 | Neg | *RUNX1-RUNX1T1* | 46,XX,t(8;21)(q22;q22)[20] | BM | Targeted NGS and PCR |
| 43 | Neg | *RUNX1-RUNX1T1* | NA | BM | Targeted NGS and PCR |
| 44 | *RUNX1, RAD21* | Neg | 46,XY[20] | BM & Fresh MS tissue | Targeted NGS, PCR and RNA-seq |
| 45 | *NPM1* | *RUNX1-RUNX1T1* | NA | FFPE | RNA-seq |
| 46 | *CEBPA, SRSF2, RUNX1, TET2, ELANE* | Neg | NA | FFPE | RNA-seq |
| 47 | *C-kit* | *RUNX1-RUNX1T1* | NA | FFPE | RNA-seq |
| 48 | *C-kit* | *RUNX1-RUNX1T1* | 46,XY,t(8;21)(q22;q22)[20] | BM | Targeted NGS and PCR |
| 49 | *Neg* | *FUS-ERG* | 46,XY,der(10)t(1; 10)(q25;q26),t(16;21)(p11.2; q22)[10] | BM & FFPE | Targeted NGS, PCR and RNA-seq |
| 51 | *ETV6, NPM1* | Neg | NA | FFPE | RNA-seq |
| 52 | *SRSF2* | Neg | NA | FFPE | RNA-seq |
| 55 | *ETV6* | Neg | NA | FFPE | RNA-seq |
| 56 | *C-kit* | *RUNX1-RUNX1T1* | 46,XX,t(8;21)(q22;q22)[20] | BM | Targeted NGS and PCR |
| 57 | *C-kit* | *RUNX1-RUNX1T1* | 46,XY,t(8;21)(q22;q22)[20] | BM | Targeted NGS and PCR |
| 58 | *DNMT3A, ETV6, NRAS, TP53* | Neg | 46,XX[11] | BM | Targeted NGS and PCR |
| 59 | *C-Kit, TET2, KRAS* | *RUNX1-RUNX1T1* | 47,-Y,t(8; 21)(q22;q22),del(9)(q22q32),+13,+21[20] | BM | Targeted NGS and PCR |
| 60 | *C-kit* | *RUNX1-RUNX1T1* | 46,XX,t(8;21)(q22;q22)[20] | BM | Targeted NGS and PCR |
| 61 | *ETV6, NPM1* | Neg | 46,XX[20] | BM | Targeted NGS and PCR |

| Table S7: Fusion genes detected by RNA-seq in 22 MS patients | | | | |
| --- | --- | --- | --- | --- |
| Case number | Fusion Name | Junction Read Count | Spanning Fragment Count | Splice Type |
| 31 | AC005258.1--ACTB | 2 | 3 | ONLY_REF_SPLICE |
| 31 | AC005258.1--KLF2 | 5 | 0 | ONLY_REF_SPLICE |
| 45 | AC006064.5--PTCH2 | 5 | 0 | INCL_NON_REF_SPLICE |
| 45 | AC006064.5--RNU5A-1 | 9 | 0 | INCL_NON_REF_SPLICE |
| 27 | AC007952.4--RNU5B-1 | 7 | 0 | INCL_NON_REF_SPLICE |
| 34 | AC007952.4--SNHG3 | 6 | 4 | INCL_NON_REF_SPLICE |
| 27 | AC007952.4--SNORA73B | 10 | 0 | INCL_NON_REF_SPLICE |
| 31 | AC020656.1--RN7SL2 | 5 | 44 | INCL_NON_REF_SPLICE |
| 32 | AC025171.2--RN7SL2 | 5 | 0 | INCL_NON_REF_SPLICE |
| 27 | AC067930.1--RN7SKP281 | 11 | 0 | INCL_NON_REF_SPLICE |
| 46 | AC098590.1--AC099789.1 | 6 | 0 | INCL_NON_REF_SPLICE |
| 49 | AC098590.1--AC099789.1 | 4 | 0 | INCL_NON_REF_SPLICE |
| 47 | AC098590.1--AC099789.1 | 5 | 0 | INCL_NON_REF_SPLICE |
| 34 | AC098590.1--AC099789.1 | 10 | 0 | INCL_NON_REF_SPLICE |
| 20 | AC098590.1--AC099789.1 | 4 | 0 | INCL_NON_REF_SPLICE |
| 24 | AC098590.1--AC099789.1 | 14 | 0 | INCL_NON_REF_SPLICE |
| 27 | AC110751.1--AC105383.1 | 6 | 1 | INCL_NON_REF_SPLICE |
| 20 | AC125603.2--AC125603.1 | 10 | 0 | ONLY_REF_SPLICE |
| 25 | AC125603.2--AC125603.1 | 7 | 0 | ONLY_REF_SPLICE |
| 31 | AL139022.1--MTND4P12 | 5 | 0 | INCL_NON_REF_SPLICE |
| 20 | AL139099.4--PLCG2 | 7 | 3 | INCL_NON_REF_SPLICE |
| 20 | AL139099.4--PLCG2 | 6 | 3 | INCL_NON_REF_SPLICE |
| 49 | AL139099.4--RF00100 | 3 | 101 | INCL_NON_REF_SPLICE |
| 49 | AL139099.4--RF00100 | 3 | 101 | INCL_NON_REF_SPLICE |
| 49 | AL139099.4--RF00100 | 3 | 101 | INCL_NON_REF_SPLICE |
| 49 | AL139099.4--RF00100 | 3 | 101 | INCL_NON_REF_SPLICE |
| 34 | AL139099.4--RF00100 | 11 | 165 | INCL_NON_REF_SPLICE |
| 34 | AL139099.4--RF00100 | 8 | 165 | INCL_NON_REF_SPLICE |
| 34 | AL139099.4--RF00100 | 7 | 165 | INCL_NON_REF_SPLICE |
| 34 | AL139099.4--RF00100 | 7 | 165 | INCL_NON_REF_SPLICE |
| 34 | AL139099.4--RF00100 | 7 | 165 | INCL_NON_REF_SPLICE |
| 34 | AL139099.4--RF00100 | 6 | 165 | INCL_NON_REF_SPLICE |
| 34 | AL139099.4--RF00100 | 6 | 165 | INCL_NON_REF_SPLICE |
| 34 | AL139099.4--RF00100 | 6 | 165 | INCL_NON_REF_SPLICE |
| 34 | AL139099.4--RF00100 | 5 | 165 | INCL_NON_REF_SPLICE |
| 34 | AL139099.4--RF00100 | 5 | 165 | INCL_NON_REF_SPLICE |
| 34 | AL139099.4--RF00100 | 5 | 165 | INCL_NON_REF_SPLICE |
| 34 | AL139099.4--RF00100 | 5 | 165 | INCL_NON_REF_SPLICE |
| 34 | AL139099.4--RF00100 | 5 | 165 | INCL_NON_REF_SPLICE |
| 34 | AL139099.4--RF00100 | 5 | 165 | INCL_NON_REF_SPLICE |
| 34 | AL139099.4--RF00100 | 5 | 165 | INCL_NON_REF_SPLICE |
| 34 | AL139099.4--RF00100 | 5 | 165 | INCL_NON_REF_SPLICE |
| 34 | AL139099.4--RF00100 | 4 | 165 | INCL_NON_REF_SPLICE |
| 34 | AL139099.4--RF00100 | 4 | 165 | INCL_NON_REF_SPLICE |
| 34 | AL139099.4--RF00100 | 4 | 165 | INCL_NON_REF_SPLICE |
| 34 | AL139099.4--RF00100 | 4 | 165 | INCL_NON_REF_SPLICE |
| 34 | AL139099.4--RF00100 | 4 | 165 | INCL_NON_REF_SPLICE |
| 34 | AL139099.4--RF00100 | 4 | 165 | INCL_NON_REF_SPLICE |
| 34 | AL139099.4--RF00100 | 4 | 165 | INCL_NON_REF_SPLICE |
| 34 | AL139099.4--RF00100 | 3 | 165 | INCL_NON_REF_SPLICE |
| 34 | AL139099.4--RF00100 | 3 | 165 | INCL_NON_REF_SPLICE |
| 34 | AL139099.4--RF00100 | 3 | 165 | INCL_NON_REF_SPLICE |
| 34 | AL139099.4--RF00100 | 3 | 165 | INCL_NON_REF_SPLICE |
| 34 | AL139099.4--RF00100 | 3 | 165 | INCL_NON_REF_SPLICE |
| 34 | AL139099.4--RF00100 | 3 | 165 | INCL_NON_REF_SPLICE |
| 34 | AL139099.4--RF00100 | 3 | 165 | INCL_NON_REF_SPLICE |
| 34 | AL139099.4--RF00100 | 3 | 165 | INCL_NON_REF_SPLICE |
| 34 | AL139099.4--RF00100 | 3 | 165 | INCL_NON_REF_SPLICE |
| 34 | AL139099.4--RF00100 | 3 | 165 | INCL_NON_REF_SPLICE |
| 34 | AL139099.4--RF00100 | 3 | 165 | INCL_NON_REF_SPLICE |
| 34 | AL139099.4--RF00100 | 3 | 165 | INCL_NON_REF_SPLICE |
| 20 | AL139099.4--RF00100 | 8 | 36 | INCL_NON_REF_SPLICE |
| 20 | AL139099.4--RF00100 | 7 | 36 | INCL_NON_REF_SPLICE |
| 20 | AL139099.4--RF00100 | 6 | 36 | INCL_NON_REF_SPLICE |
| 20 | AL139099.4--RF00100 | 5 | 36 | INCL_NON_REF_SPLICE |
| 20 | AL139099.4--RF00100 | 5 | 36 | INCL_NON_REF_SPLICE |
| 20 | AL139099.4--RF00100 | 5 | 36 | INCL_NON_REF_SPLICE |
| 20 | AL139099.4--RF00100 | 4 | 36 | INCL_NON_REF_SPLICE |
| 20 | AL139099.4--RF00100 | 4 | 36 | INCL_NON_REF_SPLICE |
| 20 | AL139099.4--RF00100 | 4 | 36 | INCL_NON_REF_SPLICE |
| 20 | AL139099.4--RF00100 | 4 | 36 | INCL_NON_REF_SPLICE |
| 20 | AL139099.4--RF00100 | 4 | 36 | INCL_NON_REF_SPLICE |
| 20 | AL139099.4--RF00100 | 4 | 36 | INCL_NON_REF_SPLICE |
| 20 | AL139099.4--RF00100 | 4 | 36 | INCL_NON_REF_SPLICE |
| 20 | AL139099.4--RF00100 | 4 | 36 | INCL_NON_REF_SPLICE |
| 20 | AL139099.4--RF00100 | 3 | 36 | INCL_NON_REF_SPLICE |
| 20 | AL139099.4--RF00100 | 3 | 36 | INCL_NON_REF_SPLICE |
| 20 | AL139099.4--RF00100 | 3 | 36 | INCL_NON_REF_SPLICE |
| 20 | AL139099.4--RF00100 | 3 | 36 | INCL_NON_REF_SPLICE |
| 20 | AL139099.4--RF00100 | 3 | 36 | INCL_NON_REF_SPLICE |
| 20 | AL139099.4--RF00100 | 3 | 36 | INCL_NON_REF_SPLICE |
| 20 | AL139099.4--RF00100 | 3 | 36 | INCL_NON_REF_SPLICE |
| 20 | AL139099.4--RF00100 | 3 | 36 | INCL_NON_REF_SPLICE |
| 30 | AL139099.4--RMRP | 5 | 16 | INCL_NON_REF_SPLICE |
| 30 | AL139099.4--RMRP | 4 | 16 | INCL_NON_REF_SPLICE |
| 30 | AL139099.4--RMRP | 3 | 16 | INCL_NON_REF_SPLICE |
| 30 | AL139099.4--RMRP | 3 | 16 | INCL_NON_REF_SPLICE |
| 30 | AL139099.4--RMRP | 3 | 16 | INCL_NON_REF_SPLICE |
| 30 | AL139099.4--RMRP | 3 | 16 | INCL_NON_REF_SPLICE |
| 30 | AL139099.4--RMRP | 3 | 16 | INCL_NON_REF_SPLICE |
| 20 | AL139099.4--RMRP | 11 | 26 | INCL_NON_REF_SPLICE |
| 20 | AL139099.4--RMRP | 8 | 26 | INCL_NON_REF_SPLICE |
| 20 | AL139099.4--RMRP | 5 | 26 | INCL_NON_REF_SPLICE |
| 20 | AL139099.4--RMRP | 4 | 26 | INCL_NON_REF_SPLICE |
| 20 | AL139099.4--RMRP | 4 | 26 | INCL_NON_REF_SPLICE |
| 20 | AL139099.4--RMRP | 3 | 26 | INCL_NON_REF_SPLICE |
| 20 | AL139099.4--RMRP | 3 | 26 | INCL_NON_REF_SPLICE |
| 20 | AL139099.4--RMRP | 3 | 26 | INCL_NON_REF_SPLICE |
| 20 | AL139099.4--RMRP | 3 | 26 | INCL_NON_REF_SPLICE |
| 20 | AL139099.4--RMRP | 3 | 26 | INCL_NON_REF_SPLICE |
| 20 | AL139099.4--RMRP | 3 | 26 | INCL_NON_REF_SPLICE |
| 20 | AL139099.4--RMRP | 3 | 26 | INCL_NON_REF_SPLICE |
| 49 | AL139099.4--RN7SK | 3 | 101 | INCL_NON_REF_SPLICE |
| 49 | AL139099.4--RN7SK | 3 | 101 | INCL_NON_REF_SPLICE |
| 49 | AL139099.4--RN7SK | 3 | 101 | INCL_NON_REF_SPLICE |
| 49 | AL139099.4--RN7SK | 3 | 101 | INCL_NON_REF_SPLICE |
| 34 | AL139099.4--RN7SK | 11 | 165 | INCL_NON_REF_SPLICE |
| 34 | AL139099.4--RN7SK | 8 | 165 | INCL_NON_REF_SPLICE |
| 34 | AL139099.4--RN7SK | 7 | 165 | INCL_NON_REF_SPLICE |
| 34 | AL139099.4--RN7SK | 7 | 165 | INCL_NON_REF_SPLICE |
| 34 | AL139099.4--RN7SK | 7 | 165 | INCL_NON_REF_SPLICE |
| 34 | AL139099.4--RN7SK | 6 | 165 | INCL_NON_REF_SPLICE |
| 34 | AL139099.4--RN7SK | 6 | 165 | INCL_NON_REF_SPLICE |
| 34 | AL139099.4--RN7SK | 6 | 165 | INCL_NON_REF_SPLICE |
| 34 | AL139099.4--RN7SK | 5 | 165 | INCL_NON_REF_SPLICE |
| 34 | AL139099.4--RN7SK | 5 | 165 | INCL_NON_REF_SPLICE |
| 34 | AL139099.4--RN7SK | 5 | 165 | INCL_NON_REF_SPLICE |
| 34 | AL139099.4--RN7SK | 5 | 165 | INCL_NON_REF_SPLICE |
| 34 | AL139099.4--RN7SK | 5 | 165 | INCL_NON_REF_SPLICE |
| 34 | AL139099.4--RN7SK | 5 | 165 | INCL_NON_REF_SPLICE |
| 34 | AL139099.4--RN7SK | 5 | 165 | INCL_NON_REF_SPLICE |
| 34 | AL139099.4--RN7SK | 5 | 165 | INCL_NON_REF_SPLICE |
| 34 | AL139099.4--RN7SK | 4 | 165 | INCL_NON_REF_SPLICE |
| 34 | AL139099.4--RN7SK | 4 | 165 | INCL_NON_REF_SPLICE |
| 34 | AL139099.4--RN7SK | 4 | 165 | INCL_NON_REF_SPLICE |
| 34 | AL139099.4--RN7SK | 4 | 165 | INCL_NON_REF_SPLICE |
| 34 | AL139099.4--RN7SK | 4 | 165 | INCL_NON_REF_SPLICE |
| 34 | AL139099.4--RN7SK | 4 | 165 | INCL_NON_REF_SPLICE |
| 34 | AL139099.4--RN7SK | 4 | 165 | INCL_NON_REF_SPLICE |
| 34 | AL139099.4--RN7SK | 3 | 165 | INCL_NON_REF_SPLICE |
| 34 | AL139099.4--RN7SK | 3 | 165 | INCL_NON_REF_SPLICE |
| 34 | AL139099.4--RN7SK | 3 | 165 | INCL_NON_REF_SPLICE |
| 34 | AL139099.4--RN7SK | 3 | 165 | INCL_NON_REF_SPLICE |
| 34 | AL139099.4--RN7SK | 3 | 165 | INCL_NON_REF_SPLICE |
| 34 | AL139099.4--RN7SK | 3 | 165 | INCL_NON_REF_SPLICE |
| 34 | AL139099.4--RN7SK | 3 | 165 | INCL_NON_REF_SPLICE |
| 34 | AL139099.4--RN7SK | 3 | 165 | INCL_NON_REF_SPLICE |
| 34 | AL139099.4--RN7SK | 3 | 165 | INCL_NON_REF_SPLICE |
| 34 | AL139099.4--RN7SK | 3 | 165 | INCL_NON_REF_SPLICE |
| 34 | AL139099.4--RN7SK | 3 | 165 | INCL_NON_REF_SPLICE |
| 34 | AL139099.4--RN7SK | 3 | 165 | INCL_NON_REF_SPLICE |
| 20 | AL139099.4--RN7SK | 8 | 36 | INCL_NON_REF_SPLICE |
| 20 | AL139099.4--RN7SK | 7 | 36 | INCL_NON_REF_SPLICE |
| 20 | AL139099.4--RN7SK | 6 | 36 | INCL_NON_REF_SPLICE |
| 20 | AL139099.4--RN7SK | 5 | 36 | INCL_NON_REF_SPLICE |
| 20 | AL139099.4--RN7SK | 5 | 36 | INCL_NON_REF_SPLICE |
| 20 | AL139099.4--RN7SK | 5 | 36 | INCL_NON_REF_SPLICE |
| 20 | AL139099.4--RN7SK | 4 | 36 | INCL_NON_REF_SPLICE |
| 20 | AL139099.4--RN7SK | 4 | 36 | INCL_NON_REF_SPLICE |
| 20 | AL139099.4--RN7SK | 4 | 36 | INCL_NON_REF_SPLICE |
| 20 | AL139099.4--RN7SK | 4 | 36 | INCL_NON_REF_SPLICE |
| 20 | AL139099.4--RN7SK | 4 | 36 | INCL_NON_REF_SPLICE |
| 20 | AL139099.4--RN7SK | 4 | 36 | INCL_NON_REF_SPLICE |
| 20 | AL139099.4--RN7SK | 4 | 36 | INCL_NON_REF_SPLICE |
| 20 | AL139099.4--RN7SK | 4 | 36 | INCL_NON_REF_SPLICE |
| 20 | AL139099.4--RN7SK | 3 | 36 | INCL_NON_REF_SPLICE |
| 20 | AL139099.4--RN7SK | 3 | 36 | INCL_NON_REF_SPLICE |
| 20 | AL139099.4--RN7SK | 3 | 36 | INCL_NON_REF_SPLICE |
| 20 | AL139099.4--RN7SK | 3 | 36 | INCL_NON_REF_SPLICE |
| 20 | AL139099.4--RN7SK | 3 | 36 | INCL_NON_REF_SPLICE |
| 20 | AL139099.4--RN7SK | 3 | 36 | INCL_NON_REF_SPLICE |
| 20 | AL139099.4--RN7SK | 3 | 36 | INCL_NON_REF_SPLICE |
| 20 | AL139099.4--RN7SK | 3 | 36 | INCL_NON_REF_SPLICE |
| 30 | AL139099.4--RNU5B-1 | 9 | 0 | INCL_NON_REF_SPLICE |
| 34 | AL139099.4--SNORA73B | 33 | 39 | INCL_NON_REF_SPLICE |
| 34 | AL139099.4--SNORA73B | 19 | 39 | INCL_NON_REF_SPLICE |
| 34 | AL139099.4--SNORA73B | 17 | 39 | INCL_NON_REF_SPLICE |
| 34 | AL139099.4--SNORA73B | 12 | 39 | INCL_NON_REF_SPLICE |
| 34 | AL139099.4--SNORA73B | 10 | 39 | INCL_NON_REF_SPLICE |
| 34 | AL139099.4--SNORA73B | 10 | 39 | INCL_NON_REF_SPLICE |
| 34 | AL139099.4--SNORA73B | 10 | 39 | INCL_NON_REF_SPLICE |
| 34 | AL139099.4--SNORA73B | 9 | 39 | INCL_NON_REF_SPLICE |
| 34 | AL139099.4--SNORA73B | 9 | 39 | INCL_NON_REF_SPLICE |
| 34 | AL139099.4--SNORA73B | 7 | 39 | INCL_NON_REF_SPLICE |
| 34 | AL355075.4--AC007952.4 | 6 | 0 | INCL_NON_REF_SPLICE |
| 49 | AL355075.4--AL139099.4 | 3 | 72 | INCL_NON_REF_SPLICE |
| 30 | AL355075.4--PLCG2 | 3 | 3 | INCL_NON_REF_SPLICE |
| 34 | AL355075.4--PLCG2 | 4 | 30 | INCL_NON_REF_SPLICE |
| 34 | AL355075.4--PLCG2 | 4 | 30 | INCL_NON_REF_SPLICE |
| 27 | AL355075.4--PLCG2 | 14 | 19 | INCL_NON_REF_SPLICE |
| 30 | AL355075.4--RF00100 | 3 | 10 | INCL_NON_REF_SPLICE |
| 30 | AL355075.4--RF00100 | 3 | 10 | INCL_NON_REF_SPLICE |
| 30 | AL355075.4--RF00100 | 3 | 10 | INCL_NON_REF_SPLICE |
| 34 | AL355075.4--RF00100 | 7 | 70 | INCL_NON_REF_SPLICE |
| 34 | AL355075.4--RF00100 | 5 | 70 | INCL_NON_REF_SPLICE |
| 34 | AL355075.4--RF00100 | 4 | 70 | INCL_NON_REF_SPLICE |
| 34 | AL355075.4--RF00100 | 4 | 70 | INCL_NON_REF_SPLICE |
| 27 | AL355075.4--RF00100 | 15 | 45 | INCL_NON_REF_SPLICE |
| 27 | AL355075.4--RF00100 | 7 | 45 | INCL_NON_REF_SPLICE |
| 27 | AL355075.4--RF00100 | 7 | 45 | INCL_NON_REF_SPLICE |
| 27 | AL355075.4--RF00100 | 6 | 45 | INCL_NON_REF_SPLICE |
| 27 | AL355075.4--RF00100 | 6 | 45 | INCL_NON_REF_SPLICE |
| 27 | AL355075.4--RF00100 | 6 | 45 | INCL_NON_REF_SPLICE |
| 27 | AL355075.4--RF00100 | 4 | 45 | INCL_NON_REF_SPLICE |
| 27 | AL355075.4--RF00100 | 4 | 45 | INCL_NON_REF_SPLICE |
| 27 | AL355075.4--RF00100 | 4 | 45 | INCL_NON_REF_SPLICE |
| 27 | AL355075.4--RF00100 | 4 | 45 | INCL_NON_REF_SPLICE |
| 27 | AL355075.4--RF00100 | 4 | 45 | INCL_NON_REF_SPLICE |
| 27 | AL355075.4--RF00100 | 4 | 45 | INCL_NON_REF_SPLICE |
| 27 | AL355075.4--RF00100 | 3 | 45 | INCL_NON_REF_SPLICE |
| 27 | AL355075.4--RF00100 | 3 | 45 | INCL_NON_REF_SPLICE |
| 27 | AL355075.4--RF00100 | 3 | 45 | INCL_NON_REF_SPLICE |
| 27 | AL355075.4--RF00100 | 3 | 45 | INCL_NON_REF_SPLICE |
| 27 | AL355075.4--RF00100 | 3 | 45 | INCL_NON_REF_SPLICE |
| 27 | AL355075.4--RF00100 | 3 | 45 | INCL_NON_REF_SPLICE |
| 27 | AL355075.4--RF00100 | 3 | 45 | INCL_NON_REF_SPLICE |
| 27 | AL355075.4--RF00100 | 3 | 45 | INCL_NON_REF_SPLICE |
| 27 | AL355075.4--RF00100 | 3 | 45 | INCL_NON_REF_SPLICE |
| 27 | AL355075.4--RF00100 | 3 | 45 | INCL_NON_REF_SPLICE |
| 27 | AL355075.4--RF00100 | 3 | 45 | INCL_NON_REF_SPLICE |
| 30 | AL355075.4--RN7SK | 3 | 10 | INCL_NON_REF_SPLICE |
| 30 | AL355075.4--RN7SK | 3 | 10 | INCL_NON_REF_SPLICE |
| 30 | AL355075.4--RN7SK | 3 | 10 | INCL_NON_REF_SPLICE |
| 34 | AL355075.4--RN7SK | 7 | 70 | INCL_NON_REF_SPLICE |
| 34 | AL355075.4--RN7SK | 5 | 70 | INCL_NON_REF_SPLICE |
| 34 | AL355075.4--RN7SK | 4 | 70 | INCL_NON_REF_SPLICE |
| 34 | AL355075.4--RN7SK | 4 | 70 | INCL_NON_REF_SPLICE |
| 27 | AL355075.4--RN7SK | 15 | 45 | INCL_NON_REF_SPLICE |
| 27 | AL355075.4--RN7SK | 7 | 45 | INCL_NON_REF_SPLICE |
| 27 | AL355075.4--RN7SK | 7 | 45 | INCL_NON_REF_SPLICE |
| 27 | AL355075.4--RN7SK | 6 | 45 | INCL_NON_REF_SPLICE |
| 27 | AL355075.4--RN7SK | 6 | 45 | INCL_NON_REF_SPLICE |
| 27 | AL355075.4--RN7SK | 6 | 45 | INCL_NON_REF_SPLICE |
| 27 | AL355075.4--RN7SK | 4 | 45 | INCL_NON_REF_SPLICE |
| 27 | AL355075.4--RN7SK | 4 | 45 | INCL_NON_REF_SPLICE |
| 27 | AL355075.4--RN7SK | 4 | 45 | INCL_NON_REF_SPLICE |
| 27 | AL355075.4--RN7SK | 4 | 45 | INCL_NON_REF_SPLICE |
| 27 | AL355075.4--RN7SK | 4 | 45 | INCL_NON_REF_SPLICE |
| 27 | AL355075.4--RN7SK | 4 | 45 | INCL_NON_REF_SPLICE |
| 27 | AL355075.4--RN7SK | 3 | 45 | INCL_NON_REF_SPLICE |
| 27 | AL355075.4--RN7SK | 3 | 45 | INCL_NON_REF_SPLICE |
| 27 | AL355075.4--RN7SK | 3 | 45 | INCL_NON_REF_SPLICE |
| 27 | AL355075.4--RN7SK | 3 | 45 | INCL_NON_REF_SPLICE |
| 27 | AL355075.4--RN7SK | 3 | 45 | INCL_NON_REF_SPLICE |
| 27 | AL355075.4--RN7SK | 3 | 45 | INCL_NON_REF_SPLICE |
| 27 | AL355075.4--RN7SK | 3 | 45 | INCL_NON_REF_SPLICE |
| 27 | AL355075.4--RN7SK | 3 | 45 | INCL_NON_REF_SPLICE |
| 27 | AL355075.4--RN7SK | 3 | 45 | INCL_NON_REF_SPLICE |
| 27 | AL355075.4--RN7SK | 3 | 45 | INCL_NON_REF_SPLICE |
| 27 | AL355075.4--RN7SK | 3 | 45 | INCL_NON_REF_SPLICE |
| 49 | AL355075.4--RN7SL1 | 3 | 72 | INCL_NON_REF_SPLICE |
| 34 | AL355075.4--RN7SL2 | 8 | 90 | INCL_NON_REF_SPLICE |
| 34 | AL355075.4--RN7SL2 | 4 | 90 | INCL_NON_REF_SPLICE |
| 34 | AL355075.4--RN7SL2 | 3 | 90 | INCL_NON_REF_SPLICE |
| 34 | AL355075.4--RN7SL2 | 3 | 90 | INCL_NON_REF_SPLICE |
| 34 | AL355075.4--RN7SL2 | 3 | 90 | INCL_NON_REF_SPLICE |
| 34 | AL355075.4--RN7SL2 | 3 | 90 | INCL_NON_REF_SPLICE |
| 30 | AL355075.4--RN7SL3 | 4 | 10 | INCL_NON_REF_SPLICE |
| 30 | AL355075.4--RN7SL3 | 3 | 10 | INCL_NON_REF_SPLICE |
| 30 | AL355075.4--RN7SL3 | 3 | 10 | INCL_NON_REF_SPLICE |
| 27 | AL355075.4--RN7SL3 | 10 | 82 | INCL_NON_REF_SPLICE |
| 27 | AL355075.4--RN7SL3 | 9 | 82 | INCL_NON_REF_SPLICE |
| 27 | AL355075.4--RN7SL3 | 7 | 82 | INCL_NON_REF_SPLICE |
| 27 | AL355075.4--RN7SL3 | 7 | 82 | INCL_NON_REF_SPLICE |
| 27 | AL355075.4--RN7SL3 | 7 | 82 | INCL_NON_REF_SPLICE |
| 27 | AL355075.4--RN7SL3 | 6 | 82 | INCL_NON_REF_SPLICE |
| 27 | AL355075.4--RN7SL3 | 5 | 82 | INCL_NON_REF_SPLICE |
| 27 | AL355075.4--RN7SL3 | 5 | 82 | INCL_NON_REF_SPLICE |
| 27 | AL355075.4--RN7SL3 | 5 | 82 | INCL_NON_REF_SPLICE |
| 27 | AL355075.4--RN7SL3 | 5 | 82 | INCL_NON_REF_SPLICE |
| 27 | AL355075.4--RN7SL3 | 5 | 82 | INCL_NON_REF_SPLICE |
| 27 | AL355075.4--RN7SL3 | 5 | 82 | INCL_NON_REF_SPLICE |
| 27 | AL355075.4--RN7SL3 | 5 | 82 | INCL_NON_REF_SPLICE |
| 27 | AL355075.4--RN7SL3 | 5 | 82 | INCL_NON_REF_SPLICE |
| 27 | AL355075.4--RN7SL3 | 4 | 82 | INCL_NON_REF_SPLICE |
| 27 | AL355075.4--RN7SL3 | 4 | 82 | INCL_NON_REF_SPLICE |
| 27 | AL355075.4--RN7SL3 | 4 | 82 | INCL_NON_REF_SPLICE |
| 27 | AL355075.4--RN7SL3 | 3 | 82 | INCL_NON_REF_SPLICE |
| 27 | AL355075.4--RN7SL3 | 3 | 82 | INCL_NON_REF_SPLICE |
| 27 | AL355075.4--RN7SL3 | 3 | 82 | INCL_NON_REF_SPLICE |
| 27 | AL355075.4--RN7SL3 | 3 | 82 | INCL_NON_REF_SPLICE |
| 27 | AL355075.4--RN7SL3 | 3 | 82 | INCL_NON_REF_SPLICE |
| 27 | AL355075.4--RN7SL3 | 3 | 82 | INCL_NON_REF_SPLICE |
| 27 | AL355075.4--RN7SL3 | 3 | 82 | INCL_NON_REF_SPLICE |
| 27 | AL355075.4--RN7SL3 | 3 | 82 | INCL_NON_REF_SPLICE |
| 27 | AL355075.4--RN7SL3 | 3 | 82 | INCL_NON_REF_SPLICE |
| 27 | AL355075.4--RN7SL3 | 3 | 82 | INCL_NON_REF_SPLICE |
| 27 | AL355075.4--RN7SL3 | 3 | 82 | INCL_NON_REF_SPLICE |
| 27 | AL355075.4--RN7SL3 | 3 | 82 | INCL_NON_REF_SPLICE |
| 27 | AL355075.4--RN7SL3 | 3 | 82 | INCL_NON_REF_SPLICE |
| 27 | AL355075.4--RN7SL3 | 3 | 82 | INCL_NON_REF_SPLICE |
| 27 | AL355075.4--RN7SL3 | 3 | 82 | INCL_NON_REF_SPLICE |
| 27 | AL355075.4--RN7SL3 | 3 | 82 | INCL_NON_REF_SPLICE |
| 27 | AL355075.4--RN7SL3 | 3 | 82 | INCL_NON_REF_SPLICE |
| 32 | AL355075.4--RN7SL4P | 3 | 1 | INCL_NON_REF_SPLICE |
| 34 | AL355075.4--RNU4-1 | 5 | 0 | INCL_NON_REF_SPLICE |
| 31 | AL669831.3--AC008038.1 | 5 | 1 | INCL_NON_REF_SPLICE |
| 25 | AL669831.3--AC008038.1 | 4 | 2 | INCL_NON_REF_SPLICE |
| 31 | AL669831.3--ELANE | 9 | 1 | INCL_NON_REF_SPLICE |
| 31 | AL669831.3--ELANE | 8 | 1 | INCL_NON_REF_SPLICE |
| 31 | AL669831.3--LUC7L3 | 3 | 2 | INCL_NON_REF_SPLICE |
| 24 | AL669831.3--RPS6 | 4 | 1 | INCL_NON_REF_SPLICE |
| 44 | AL669831.3--TXNIP | 3 | 0 | INCL_NON_REF_SPLICE |
| 44 | AL671762.1--UBB | 3 | 2 | ONLY_REF_SPLICE |
| 44 | ALAS2--MTND4P24 | 4 | 0 | INCL_NON_REF_SPLICE |
| 55 | ATP13A1--BANF1 | 6 | 0 | INCL_NON_REF_SPLICE |
| 31 | AZU1--AL139099.4 | 53 | 34 | INCL_NON_REF_SPLICE |
| 31 | AZU1--AL139099.4 | 27 | 34 | INCL_NON_REF_SPLICE |
| 55 | AZU1--CAPN1 | 6 | 0 | INCL_NON_REF_SPLICE |
| 31 | AZU1--RN7SL1 | 53 | 34 | INCL_NON_REF_SPLICE |
| 31 | AZU1--RN7SL1 | 27 | 34 | INCL_NON_REF_SPLICE |
| 28 | AZU1--RN7SL2 | 8 | 2 | INCL_NON_REF_SPLICE |
| 51 | AZU1--RN7SL2 | 8 | 0 | INCL_NON_REF_SPLICE |
| 47 | AZU1--RN7SL2 | 8 | 1 | INCL_NON_REF_SPLICE |
| 47 | AZU1--RN7SL2 | 5 | 1 | INCL_NON_REF_SPLICE |
| 41 | B2M--HBA1 | 38 | 0 | INCL_NON_REF_SPLICE |
| 52 | C12orf23--RIC8B | 8 | 0 | ONLY_REF_SPLICE |
| 41 | C17orf76-AS1--RN7SL2 | 4 | 1 | INCL_NON_REF_SPLICE |
| 32 | CAPN5--RN7SL3 | 5 | 0 | INCL_NON_REF_SPLICE |
| 25 | CBFB--MYH11 | 18 | 0 | ONLY_REF_SPLICE |
| 25 | CBFB--MYH11 | 5 | 0 | ONLY_REF_SPLICE |
| 32 | CD74--DCAF17 | 4 | 0 | INCL_NON_REF_SPLICE |
| 24 | CNNM2--RF02271 | 5 | 0 | INCL_NON_REF_SPLICE |
| 55 | COIL--XXYLT1 | 7 | 0 | INCL_NON_REF_SPLICE |
| 52 | DCTN2--MARS | 38 | 13 | ONLY_REF_SPLICE |
| 52 | DCTN2--MARS | 5 | 13 | ONLY_REF_SPLICE |
| 52 | DCTN2--TSPAN8 | 6 | 0 | ONLY_REF_SPLICE |
| 44 | DCUN1D1--UBB | 4 | 0 | ONLY_REF_SPLICE |
| 28 | DEFA3--SRGN | 4 | 1 | INCL_NON_REF_SPLICE |
| 55 | DNPH1--ARPC2 | 7 | 0 | INCL_NON_REF_SPLICE |
| 48 | E2F4--RPL14 | 11 | 0 | INCL_NON_REF_SPLICE |
| 24 | EEF1A1--MTCO1P12 | 6 | 4 | INCL_NON_REF_SPLICE |
| 24 | EEF1A1--MTCO1P12 | 3 | 4 | INCL_NON_REF_SPLICE |
| 24 | EEF1A1P5--AL138963.3 | 8 | 2 | INCL_NON_REF_SPLICE |
| 41 | EEF2--HBA1 | 13 | 0 | INCL_NON_REF_SPLICE |
| 20 | EIF4A1--RNU1-1 | 8 | 0 | INCL_NON_REF_SPLICE |
| 20 | EIF4A1--RNU1-1 | 5 | 0 | INCL_NON_REF_SPLICE |
| 20 | EIF4A2--RF00003 | 4 | 0 | INCL_NON_REF_SPLICE |
| 27 | EIF4A2--RNU4-1 | 6 | 0 | INCL_NON_REF_SPLICE |
| 31 | ELANE--CTDSP1 | 5 | 0 | ONLY_REF_SPLICE |
| 41 | ETV6--MECOM | 40 | 12 | ONLY_REF_SPLICE |
| 41 | ETV6--MECOM | 20 | 12 | ONLY_REF_SPLICE |
| 55 | FANCA--ACTB | 5 | 0 | INCL_NON_REF_SPLICE |
| 32 | FLII--PTPRC | 4 | 0 | INCL_NON_REF_SPLICE |
| 31 | FUS--AC016876.2 | 5 | 0 | ONLY_REF_SPLICE |
| 31 | FUS--EIF4A1 | 5 | 0 | ONLY_REF_SPLICE |
| 49 | FUS--ERG | 4 | 0 | ONLY_REF_SPLICE |
| 31 | FUS--SENP3-EIF4A1 | 5 | 0 | ONLY_REF_SPLICE |
| 55 | GSN--ING1 | 7 | 0 | INCL_NON_REF_SPLICE |
| 44 | HBM--MTATP8P2 | 6 | 0 | INCL_NON_REF_SPLICE |
| 44 | HBZ--MTATP8P2 | 6 | 0 | INCL_NON_REF_SPLICE |
| 44 | HBZ--MTCO2P12 | 3 | 0 | INCL_NON_REF_SPLICE |
| 38 | HNRNPLL--TEX261 | 5 | 0 | INCL_NON_REF_SPLICE |
| 55 | HNRNPM--TNKS2 | 11 | 0 | INCL_NON_REF_SPLICE |
| 41 | HOXD11--AGAP3 | 6 | 0 | INCL_NON_REF_SPLICE |
| 46 | HTD2--RN7SL2 | 16 | 0 | INCL_NON_REF_SPLICE |
| 25 | IGH@--HBA1 | 4 | 1 | INCL_NON_REF_SPLICE |
| 28 | IGH@--S100A9 | 4 | 1 | INCL_NON_REF_SPLICE |
| 25 | IGHA1--HBA1 | 4 | 1 | INCL_NON_REF_SPLICE |
| 28 | IGHG1--S100A9 | 4 | 1 | INCL_NON_REF_SPLICE |
| 52 | IGKV3D-11--AC096579.7 | 9 | 0 | INCL_NON_REF_SPLICE |
| 52 | IGKV4-1--AC096579.7 | 6 | 7 | INCL_NON_REF_SPLICE |
| 25 | IGL-@--PRAMENP | 4 | 5 | INCL_NON_REF_SPLICE |
| 44 | KCNAB2--PARK7 | 2 | 3 | ONLY_REF_SPLICE |
| 44 | KCNAB2--PARK7 | 1 | 3 | ONLY_REF_SPLICE |
| 44 | KCNAB2--PARK7 | 1 | 3 | ONLY_REF_SPLICE |
| 52 | KCNMB4--LGR5 | 32 | 5 | ONLY_REF_SPLICE |
| 32 | LAMTOR5-AS1--RF00100 | 4 | 0 | INCL_NON_REF_SPLICE |
| 32 | LAMTOR5-AS1--RN7SK | 4 | 0 | INCL_NON_REF_SPLICE |
| 52 | LIN7A--TSPAN8 | 194 | 33 | ONLY_REF_SPLICE |
| 47 | LRRC75A-AS1--RN7SL2 | 4 | 1 | INCL_NON_REF_SPLICE |
| 38 | LY6E--NFATC3 | 11 | 0 | INCL_NON_REF_SPLICE |
| 38 | LY6E--RP11-96D1.10 | 11 | 0 | INCL_NON_REF_SPLICE |
| 45 | LYN--RIPK2 | 6 | 0 | ONLY_REF_SPLICE |
| 38 | MAN1A2--KLHL42 | 6 | 0 | INCL_NON_REF_SPLICE |
| 52 | METTL25--RP11-543H12.1 | 6 | 1 | INCL_NON_REF_SPLICE |
| 25 | MIR663A--MIR3687 | 16 | 0 | INCL_NON_REF_SPLICE |
| 25 | MIR663A--MIR3687 | 10 | 0 | INCL_NON_REF_SPLICE |
| 24 | MIR6723--HBB | 5 | 0 | INCL_NON_REF_SPLICE |
| 24 | MIR6723--RPL7P23 | 6 | 0 | INCL_NON_REF_SPLICE |
| 31 | MIR6723--RPS6 | 5 | 1 | INCL_NON_REF_SPLICE |
| 24 | MIR6723--RPS6 | 5 | 1 | INCL_NON_REF_SPLICE |
| 27 | MLLT10--PICALM | 14 | 0 | ONLY_REF_SPLICE |
| 27 | MLLT10--PICALM | 5 | 0 | ONLY_REF_SPLICE |
| 27 | MLLT10--PICALM | 5 | 0 | ONLY_REF_SPLICE |
| 20 | MPO--AL139099.4 | 3 | 28 | INCL_NON_REF_SPLICE |
| 20 | MPO--RN7SL1 | 3 | 28 | INCL_NON_REF_SPLICE |
| 34 | MPO--RN7SL2 | 6 | 42 | INCL_NON_REF_SPLICE |
| 34 | MTCO1P12--RNA5-8SN2 | 3 | 4 | INCL_NON_REF_SPLICE |
| 25 | MTCO3P12--RPL29 | 8 | 6 | INCL_NON_REF_SPLICE |
| 24 | MTCO3P12--RPL5 | 6 | 1 | INCL_NON_REF_SPLICE |
| 24 | MTND2P5--S100A4 | 5 | 0 | INCL_NON_REF_SPLICE |
| 52 | MYO10--RP11-272B17.2 | 49 | 11 | INCL_NON_REF_SPLICE |
| 52 | MYO10--TTC23L | 18 | 4 | ONLY_REF_SPLICE |
| 52 | MYO10--TTC23L | 5 | 4 | ONLY_REF_SPLICE |
| 41 | NRIP1--AF127936.7 | 17 | 3 | ONLY_REF_SPLICE |
| 25 | NRIP1--AF127936.7 | 17 | 0 | ONLY_REF_SPLICE |
| 55 | NVL--ADAMTS3 | 3 | 2 | INCL_NON_REF_SPLICE |
| 31 | OAZ1--ACTB | 2 | 3 | ONLY_REF_SPLICE |
| 52 | OXCT1--FGF10 | 10 | 0 | ONLY_REF_SPLICE |
| 24 | PFN1--HBA2 | 3 | 3 | INCL_NON_REF_SPLICE |
| 27 | PICALM--MLLT10 | 16 | 0 | ONLY_REF_SPLICE |
| 20 | PLCG2--AL139099.4 | 5 | 8 | INCL_NON_REF_SPLICE |
| 34 | PLCG2--AL355075.4 | 4 | 40 | INCL_NON_REF_SPLICE |
| 27 | PLCG2--AL355075.4 | 7 | 39 | INCL_NON_REF_SPLICE |
| 27 | PLCG2--AL355075.4 | 4 | 39 | INCL_NON_REF_SPLICE |
| 27 | PLCG2--AL355075.4 | 4 | 39 | INCL_NON_REF_SPLICE |
| 27 | PLCG2--AL355075.4 | 3 | 39 | INCL_NON_REF_SPLICE |
| 27 | PLCG2--AL355075.4 | 3 | 39 | INCL_NON_REF_SPLICE |
| 27 | PLCG2--AL355075.4 | 3 | 39 | INCL_NON_REF_SPLICE |
| 34 | PLCG2--RMRP | 9 | 119 | INCL_NON_REF_SPLICE |
| 34 | PLCG2--RMRP | 8 | 119 | INCL_NON_REF_SPLICE |
| 34 | PLCG2--RMRP | 6 | 119 | INCL_NON_REF_SPLICE |
| 34 | PLCG2--RMRP | 4 | 119 | INCL_NON_REF_SPLICE |
| 34 | PLCG2--RMRP | 3 | 119 | INCL_NON_REF_SPLICE |
| 34 | PLCG2--RMRP | 3 | 119 | INCL_NON_REF_SPLICE |
| 34 | PLCG2--RMRP | 3 | 119 | INCL_NON_REF_SPLICE |
| 34 | PLCG2--RMRP | 3 | 119 | INCL_NON_REF_SPLICE |
| 27 | PLCG2--RMRP | 9 | 11 | INCL_NON_REF_SPLICE |
| 49 | PLCG2--RN7SKP227 | 3 | 48 | INCL_NON_REF_SPLICE |
| 45 | PLCG2--RN7SKP267 | 5 | 0 | INCL_NON_REF_SPLICE |
| 30 | PLCG2--RN7SKP48 | 57 | 0 | INCL_NON_REF_SPLICE |
| 46 | PLCG2--RN7SKP48 | 8 | 8 | INCL_NON_REF_SPLICE |
| 51 | PLCG2--RN7SKP48 | 10 | 4 | INCL_NON_REF_SPLICE |
| 47 | PLCG2--RN7SKP48 | 12 | 10 | INCL_NON_REF_SPLICE |
| 27 | PLCG2--RN7SKP48 | 100 | 0 | INCL_NON_REF_SPLICE |
| 48 | PLCG2--RN7SKP48 | 9 | 10 | INCL_NON_REF_SPLICE |
| 20 | PLCG2--RN7SKP48 | 22 | 0 | INCL_NON_REF_SPLICE |
| 45 | PLCG2--RN7SKP48 | 18 | 13 | INCL_NON_REF_SPLICE |
| 20 | PLCG2--RN7SL1 | 5 | 8 | INCL_NON_REF_SPLICE |
| 34 | PLCG2--RPPH1 | 4 | 40 | INCL_NON_REF_SPLICE |
| 27 | PLCG2--RPPH1 | 7 | 39 | INCL_NON_REF_SPLICE |
| 27 | PLCG2--RPPH1 | 4 | 39 | INCL_NON_REF_SPLICE |
| 27 | PLCG2--RPPH1 | 4 | 39 | INCL_NON_REF_SPLICE |
| 27 | PLCG2--RPPH1 | 3 | 39 | INCL_NON_REF_SPLICE |
| 27 | PLCG2--RPPH1 | 3 | 39 | INCL_NON_REF_SPLICE |
| 27 | PLCG2--RPPH1 | 3 | 39 | INCL_NON_REF_SPLICE |
| 34 | PLCG2--SNHG3 | 14 | 36 | INCL_NON_REF_SPLICE |
| 34 | PLCG2--SNHG3 | 14 | 36 | INCL_NON_REF_SPLICE |
| 34 | PLCG2--SNHG3 | 8 | 36 | INCL_NON_REF_SPLICE |
| 34 | PLCG2--SNHG3 | 5 | 36 | INCL_NON_REF_SPLICE |
| 34 | PLCG2--SNHG3 | 4 | 36 | INCL_NON_REF_SPLICE |
| 34 | PLCG2--SNHG3 | 4 | 36 | INCL_NON_REF_SPLICE |
| 34 | PLCG2--SNORA73B | 18 | 21 | INCL_NON_REF_SPLICE |
| 34 | PLCG2--SNORA73B | 7 | 21 | INCL_NON_REF_SPLICE |
| 34 | PLCG2--SNORA73B | 6 | 21 | INCL_NON_REF_SPLICE |
| 52 | POLR2J2--RN7SL5P | 3 | 2 | INCL_NON_REF_SPLICE |
| 52 | PTPN11--DDIT3 | 4 | 4 | ONLY_REF_SPLICE |
| 52 | R3HDM2--SHMT2 | 4 | 1 | INCL_NON_REF_SPLICE |
| 55 | RBM3--ZNF25 | 5 | 0 | INCL_NON_REF_SPLICE |
| 34 | RF00003--AL355075.4 | 6 | 2 | INCL_NON_REF_SPLICE |
| 34 | RF00003--AL355075.4 | 3 | 2 | INCL_NON_REF_SPLICE |
| 27 | RF00003--AL355075.4 | 53 | 0 | INCL_NON_REF_SPLICE |
| 27 | RF00003--AL355075.4 | 36 | 0 | INCL_NON_REF_SPLICE |
| 27 | RF00003--EIF4A1 | 24 | 0 | INCL_NON_REF_SPLICE |
| 27 | RF00003--EIF4A1 | 18 | 0 | INCL_NON_REF_SPLICE |
| 20 | RF00003--EIF4A1 | 6 | 0 | INCL_NON_REF_SPLICE |
| 20 | RF00003--EIF4A1 | 5 | 0 | INCL_NON_REF_SPLICE |
| 20 | RF00003--EIF4A1 | 4 | 0 | INCL_NON_REF_SPLICE |
| 30 | RF00003--RNU5A-1 | 5 | 0 | INCL_NON_REF_SPLICE |
| 27 | RF00003--RNU5A-1 | 25 | 0 | INCL_NON_REF_SPLICE |
| 27 | RF00003--RNU5A-1 | 15 | 0 | INCL_NON_REF_SPLICE |
| 34 | RF00003--RPPH1 | 6 | 2 | INCL_NON_REF_SPLICE |
| 34 | RF00003--RPPH1 | 3 | 2 | INCL_NON_REF_SPLICE |
| 27 | RF00003--RPPH1 | 53 | 0 | INCL_NON_REF_SPLICE |
| 27 | RF00003--RPPH1 | 36 | 0 | INCL_NON_REF_SPLICE |
| 34 | RF00003--SNHG3 | 13 | 3 | INCL_NON_REF_SPLICE |
| 34 | RF00003--SNHG3 | 7 | 3 | INCL_NON_REF_SPLICE |
| 34 | RF00003--SNHG3 | 3 | 3 | INCL_NON_REF_SPLICE |
| 27 | RF00003--SNORA48 | 24 | 0 | INCL_NON_REF_SPLICE |
| 27 | RF00003--SNORA48 | 18 | 0 | INCL_NON_REF_SPLICE |
| 20 | RF00003--SNORA48 | 6 | 0 | INCL_NON_REF_SPLICE |
| 20 | RF00003--SNORA48 | 5 | 0 | INCL_NON_REF_SPLICE |
| 20 | RF00003--SNORA73A | 4 | 0 | INCL_NON_REF_SPLICE |
| 20 | RF00003--SNORA73A | 4 | 0 | INCL_NON_REF_SPLICE |
| 20 | RF00003--SNORA73A | 4 | 0 | INCL_NON_REF_SPLICE |
| 20 | RF00003--SNORA73A | 4 | 0 | INCL_NON_REF_SPLICE |
| 34 | RF00003--SNORA73B | 13 | 5 | INCL_NON_REF_SPLICE |
| 34 | RF00003--SNORA73B | 7 | 3 | INCL_NON_REF_SPLICE |
| 34 | RF00003--SNORA73B | 4 | 5 | INCL_NON_REF_SPLICE |
| 34 | RF00003--SNORA73B | 5 | 0 | INCL_NON_REF_SPLICE |
| 27 | RF00003--SNORA73B | 39 | 0 | INCL_NON_REF_SPLICE |
| 27 | RF00003--SNORA73B | 27 | 0 | INCL_NON_REF_SPLICE |
| 27 | RF00003--SNORA73B | 16 | 0 | INCL_NON_REF_SPLICE |
| 20 | RF00003--SNORD10 | 4 | 0 | INCL_NON_REF_SPLICE |
| 34 | RF00100--AL139099.4 | 15 | 102 | INCL_NON_REF_SPLICE |
| 34 | RF00100--AL139099.4 | 11 | 102 | INCL_NON_REF_SPLICE |
| 34 | RF00100--AL139099.4 | 11 | 102 | INCL_NON_REF_SPLICE |
| 34 | RF00100--AL139099.4 | 9 | 102 | INCL_NON_REF_SPLICE |
| 34 | RF00100--AL139099.4 | 9 | 102 | INCL_NON_REF_SPLICE |
| 34 | RF00100--AL139099.4 | 8 | 102 | INCL_NON_REF_SPLICE |
| 34 | RF00100--AL139099.4 | 8 | 102 | INCL_NON_REF_SPLICE |
| 34 | RF00100--AL139099.4 | 7 | 102 | INCL_NON_REF_SPLICE |
| 34 | RF00100--AL139099.4 | 6 | 102 | INCL_NON_REF_SPLICE |
| 34 | RF00100--AL139099.4 | 6 | 102 | INCL_NON_REF_SPLICE |
| 34 | RF00100--AL139099.4 | 5 | 102 | INCL_NON_REF_SPLICE |
| 34 | RF00100--AL139099.4 | 5 | 102 | INCL_NON_REF_SPLICE |
| 34 | RF00100--AL139099.4 | 4 | 102 | INCL_NON_REF_SPLICE |
| 34 | RF00100--AL139099.4 | 4 | 102 | INCL_NON_REF_SPLICE |
| 34 | RF00100--AL139099.4 | 4 | 102 | INCL_NON_REF_SPLICE |
| 34 | RF00100--AL139099.4 | 3 | 102 | INCL_NON_REF_SPLICE |
| 34 | RF00100--AL139099.4 | 3 | 102 | INCL_NON_REF_SPLICE |
| 34 | RF00100--AL139099.4 | 3 | 102 | INCL_NON_REF_SPLICE |
| 34 | RF00100--AL139099.4 | 3 | 102 | INCL_NON_REF_SPLICE |
| 34 | RF00100--AL139099.4 | 3 | 102 | INCL_NON_REF_SPLICE |
| 34 | RF00100--AL139099.4 | 3 | 102 | INCL_NON_REF_SPLICE |
| 34 | RF00100--AL139099.4 | 3 | 102 | INCL_NON_REF_SPLICE |
| 34 | RF00100--AL139099.4 | 3 | 102 | INCL_NON_REF_SPLICE |
| 20 | RF00100--AL139099.4 | 12 | 31 | INCL_NON_REF_SPLICE |
| 20 | RF00100--AL139099.4 | 7 | 31 | INCL_NON_REF_SPLICE |
| 20 | RF00100--AL139099.4 | 5 | 31 | INCL_NON_REF_SPLICE |
| 20 | RF00100--AL139099.4 | 4 | 31 | INCL_NON_REF_SPLICE |
| 20 | RF00100--AL139099.4 | 4 | 31 | INCL_NON_REF_SPLICE |
| 20 | RF00100--AL139099.4 | 4 | 31 | INCL_NON_REF_SPLICE |
| 20 | RF00100--AL139099.4 | 4 | 31 | INCL_NON_REF_SPLICE |
| 20 | RF00100--AL139099.4 | 3 | 31 | INCL_NON_REF_SPLICE |
| 20 | RF00100--AL139099.4 | 3 | 31 | INCL_NON_REF_SPLICE |
| 20 | RF00100--AL139099.4 | 3 | 31 | INCL_NON_REF_SPLICE |
| 20 | RF00100--AL139099.4 | 3 | 31 | INCL_NON_REF_SPLICE |
| 20 | RF00100--AL139099.4 | 3 | 31 | INCL_NON_REF_SPLICE |
| 20 | RF00100--AL139099.4 | 3 | 31 | INCL_NON_REF_SPLICE |
| 20 | RF00100--AL139099.4 | 3 | 31 | INCL_NON_REF_SPLICE |
| 20 | RF00100--AL139099.4 | 3 | 31 | INCL_NON_REF_SPLICE |
| 34 | RF00100--AL355075.4 | 6 | 64 | INCL_NON_REF_SPLICE |
| 34 | RF00100--AL355075.4 | 6 | 64 | INCL_NON_REF_SPLICE |
| 34 | RF00100--AL355075.4 | 3 | 64 | INCL_NON_REF_SPLICE |
| 34 | RF00100--AL355075.4 | 3 | 64 | INCL_NON_REF_SPLICE |
| 27 | RF00100--AL355075.4 | 13 | 52 | INCL_NON_REF_SPLICE |
| 27 | RF00100--AL355075.4 | 8 | 52 | INCL_NON_REF_SPLICE |
| 27 | RF00100--AL355075.4 | 7 | 52 | INCL_NON_REF_SPLICE |
| 27 | RF00100--AL355075.4 | 6 | 52 | INCL_NON_REF_SPLICE |
| 27 | RF00100--AL355075.4 | 6 | 52 | INCL_NON_REF_SPLICE |
| 27 | RF00100--AL355075.4 | 5 | 52 | INCL_NON_REF_SPLICE |
| 27 | RF00100--AL355075.4 | 5 | 52 | INCL_NON_REF_SPLICE |
| 27 | RF00100--AL355075.4 | 5 | 52 | INCL_NON_REF_SPLICE |
| 27 | RF00100--AL355075.4 | 4 | 52 | INCL_NON_REF_SPLICE |
| 27 | RF00100--AL355075.4 | 4 | 52 | INCL_NON_REF_SPLICE |
| 27 | RF00100--AL355075.4 | 4 | 52 | INCL_NON_REF_SPLICE |
| 27 | RF00100--AL355075.4 | 4 | 52 | INCL_NON_REF_SPLICE |
| 27 | RF00100--AL355075.4 | 4 | 52 | INCL_NON_REF_SPLICE |
| 27 | RF00100--AL355075.4 | 4 | 52 | INCL_NON_REF_SPLICE |
| 27 | RF00100--AL355075.4 | 4 | 52 | INCL_NON_REF_SPLICE |
| 27 | RF00100--AL355075.4 | 3 | 52 | INCL_NON_REF_SPLICE |
| 27 | RF00100--AL355075.4 | 3 | 52 | INCL_NON_REF_SPLICE |
| 27 | RF00100--AL355075.4 | 3 | 52 | INCL_NON_REF_SPLICE |
| 27 | RF00100--AL355075.4 | 3 | 52 | INCL_NON_REF_SPLICE |
| 27 | RF00100--AL355075.4 | 3 | 52 | INCL_NON_REF_SPLICE |
| 27 | RF00100--AL355075.4 | 3 | 52 | INCL_NON_REF_SPLICE |
| 27 | RF00100--AL355075.4 | 3 | 52 | INCL_NON_REF_SPLICE |
| 27 | RF00100--AL355075.4 | 3 | 52 | INCL_NON_REF_SPLICE |
| 27 | RF00100--AL355075.4 | 3 | 52 | INCL_NON_REF_SPLICE |
| 27 | RF00100--AL355075.4 | 3 | 52 | INCL_NON_REF_SPLICE |
| 27 | RF00100--AL355075.4 | 3 | 52 | INCL_NON_REF_SPLICE |
| 27 | RF00100--AL355075.4 | 3 | 52 | INCL_NON_REF_SPLICE |
| 49 | RF00100--CPNE3 | 4 | 0 | INCL_NON_REF_SPLICE |
| 49 | RF00100--CPNE3 | 4 | 0 | INCL_NON_REF_SPLICE |
| 20 | RF00100--RF00003 | 10 | 0 | INCL_NON_REF_SPLICE |
| 34 | RF00100--RMRP | 18 | 184 | INCL_NON_REF_SPLICE |
| 34 | RF00100--RMRP | 14 | 184 | INCL_NON_REF_SPLICE |
| 34 | RF00100--RMRP | 11 | 184 | INCL_NON_REF_SPLICE |
| 34 | RF00100--RMRP | 9 | 184 | INCL_NON_REF_SPLICE |
| 34 | RF00100--RMRP | 7 | 184 | INCL_NON_REF_SPLICE |
| 34 | RF00100--RMRP | 6 | 184 | INCL_NON_REF_SPLICE |
| 34 | RF00100--RMRP | 6 | 184 | INCL_NON_REF_SPLICE |
| 34 | RF00100--RMRP | 5 | 184 | INCL_NON_REF_SPLICE |
| 34 | RF00100--RMRP | 5 | 184 | INCL_NON_REF_SPLICE |
| 34 | RF00100--RMRP | 5 | 184 | INCL_NON_REF_SPLICE |
| 34 | RF00100--RMRP | 4 | 184 | INCL_NON_REF_SPLICE |
| 34 | RF00100--RMRP | 4 | 184 | INCL_NON_REF_SPLICE |
| 34 | RF00100--RMRP | 4 | 184 | INCL_NON_REF_SPLICE |
| 34 | RF00100--RMRP | 4 | 184 | INCL_NON_REF_SPLICE |
| 34 | RF00100--RMRP | 4 | 184 | INCL_NON_REF_SPLICE |
| 34 | RF00100--RMRP | 4 | 184 | INCL_NON_REF_SPLICE |
| 34 | RF00100--RMRP | 4 | 184 | INCL_NON_REF_SPLICE |
| 34 | RF00100--RMRP | 4 | 184 | INCL_NON_REF_SPLICE |
| 34 | RF00100--RMRP | 4 | 184 | INCL_NON_REF_SPLICE |
| 34 | RF00100--RMRP | 4 | 184 | INCL_NON_REF_SPLICE |
| 34 | RF00100--RMRP | 4 | 184 | INCL_NON_REF_SPLICE |
| 34 | RF00100--RMRP | 3 | 184 | INCL_NON_REF_SPLICE |
| 34 | RF00100--RMRP | 3 | 184 | INCL_NON_REF_SPLICE |
| 34 | RF00100--RMRP | 3 | 184 | INCL_NON_REF_SPLICE |
| 34 | RF00100--RMRP | 3 | 184 | INCL_NON_REF_SPLICE |
| 34 | RF00100--RMRP | 3 | 184 | INCL_NON_REF_SPLICE |
| 34 | RF00100--RMRP | 3 | 184 | INCL_NON_REF_SPLICE |
| 34 | RF00100--RMRP | 3 | 184 | INCL_NON_REF_SPLICE |
| 34 | RF00100--RMRP | 3 | 184 | INCL_NON_REF_SPLICE |
| 34 | RF00100--RMRP | 3 | 184 | INCL_NON_REF_SPLICE |
| 34 | RF00100--RMRP | 3 | 184 | INCL_NON_REF_SPLICE |
| 34 | RF00100--RMRP | 3 | 184 | INCL_NON_REF_SPLICE |
| 34 | RF00100--RMRP | 3 | 184 | INCL_NON_REF_SPLICE |
| 27 | RF00100--RMRP | 15 | 24 | INCL_NON_REF_SPLICE |
| 27 | RF00100--RMRP | 12 | 24 | INCL_NON_REF_SPLICE |
| 27 | RF00100--RMRP | 11 | 24 | INCL_NON_REF_SPLICE |
| 27 | RF00100--RMRP | 10 | 24 | INCL_NON_REF_SPLICE |
| 27 | RF00100--RMRP | 10 | 24 | INCL_NON_REF_SPLICE |
| 27 | RF00100--RMRP | 6 | 24 | INCL_NON_REF_SPLICE |
| 27 | RF00100--RMRP | 5 | 24 | INCL_NON_REF_SPLICE |
| 27 | RF00100--RMRP | 5 | 24 | INCL_NON_REF_SPLICE |
| 27 | RF00100--RMRP | 5 | 24 | INCL_NON_REF_SPLICE |
| 27 | RF00100--RMRP | 5 | 24 | INCL_NON_REF_SPLICE |
| 27 | RF00100--RMRP | 4 | 24 | INCL_NON_REF_SPLICE |
| 27 | RF00100--RMRP | 4 | 24 | INCL_NON_REF_SPLICE |
| 27 | RF00100--RMRP | 4 | 24 | INCL_NON_REF_SPLICE |
| 27 | RF00100--RMRP | 4 | 24 | INCL_NON_REF_SPLICE |
| 27 | RF00100--RMRP | 4 | 24 | INCL_NON_REF_SPLICE |
| 27 | RF00100--RMRP | 4 | 24 | INCL_NON_REF_SPLICE |
| 27 | RF00100--RMRP | 4 | 24 | INCL_NON_REF_SPLICE |
| 27 | RF00100--RMRP | 4 | 24 | INCL_NON_REF_SPLICE |
| 27 | RF00100--RMRP | 4 | 24 | INCL_NON_REF_SPLICE |
| 27 | RF00100--RMRP | 4 | 24 | INCL_NON_REF_SPLICE |
| 27 | RF00100--RMRP | 4 | 24 | INCL_NON_REF_SPLICE |
| 27 | RF00100--RMRP | 3 | 24 | INCL_NON_REF_SPLICE |
| 27 | RF00100--RMRP | 3 | 24 | INCL_NON_REF_SPLICE |
| 27 | RF00100--RMRP | 3 | 24 | INCL_NON_REF_SPLICE |
| 27 | RF00100--RMRP | 3 | 24 | INCL_NON_REF_SPLICE |
| 27 | RF00100--RMRP | 3 | 24 | INCL_NON_REF_SPLICE |
| 27 | RF00100--RMRP | 3 | 24 | INCL_NON_REF_SPLICE |
| 27 | RF00100--RMRP | 3 | 24 | INCL_NON_REF_SPLICE |
| 27 | RF00100--RMRP | 3 | 24 | INCL_NON_REF_SPLICE |
| 27 | RF00100--RMRP | 3 | 24 | INCL_NON_REF_SPLICE |
| 45 | RF00100--RN7SKP267 | 8 | 0 | INCL_NON_REF_SPLICE |
| 34 | RF00100--RN7SL1 | 15 | 102 | INCL_NON_REF_SPLICE |
| 34 | RF00100--RN7SL1 | 11 | 102 | INCL_NON_REF_SPLICE |
| 34 | RF00100--RN7SL1 | 11 | 102 | INCL_NON_REF_SPLICE |
| 34 | RF00100--RN7SL1 | 9 | 102 | INCL_NON_REF_SPLICE |
| 34 | RF00100--RN7SL1 | 9 | 102 | INCL_NON_REF_SPLICE |
| 34 | RF00100--RN7SL1 | 8 | 102 | INCL_NON_REF_SPLICE |
| 34 | RF00100--RN7SL1 | 8 | 102 | INCL_NON_REF_SPLICE |
| 34 | RF00100--RN7SL1 | 7 | 102 | INCL_NON_REF_SPLICE |
| 34 | RF00100--RN7SL1 | 6 | 102 | INCL_NON_REF_SPLICE |
| 34 | RF00100--RN7SL1 | 6 | 102 | INCL_NON_REF_SPLICE |
| 34 | RF00100--RN7SL1 | 5 | 102 | INCL_NON_REF_SPLICE |
| 34 | RF00100--RN7SL1 | 5 | 102 | INCL_NON_REF_SPLICE |
| 34 | RF00100--RN7SL1 | 4 | 102 | INCL_NON_REF_SPLICE |
| 34 | RF00100--RN7SL1 | 4 | 102 | INCL_NON_REF_SPLICE |
| 34 | RF00100--RN7SL1 | 4 | 102 | INCL_NON_REF_SPLICE |
| 34 | RF00100--RN7SL1 | 3 | 102 | INCL_NON_REF_SPLICE |
| 34 | RF00100--RN7SL1 | 3 | 102 | INCL_NON_REF_SPLICE |
| 34 | RF00100--RN7SL1 | 3 | 102 | INCL_NON_REF_SPLICE |
| 34 | RF00100--RN7SL1 | 3 | 102 | INCL_NON_REF_SPLICE |
| 34 | RF00100--RN7SL1 | 3 | 102 | INCL_NON_REF_SPLICE |
| 34 | RF00100--RN7SL1 | 3 | 102 | INCL_NON_REF_SPLICE |
| 34 | RF00100--RN7SL1 | 3 | 102 | INCL_NON_REF_SPLICE |
| 34 | RF00100--RN7SL1 | 3 | 102 | INCL_NON_REF_SPLICE |
| 20 | RF00100--RN7SL1 | 12 | 31 | INCL_NON_REF_SPLICE |
| 20 | RF00100--RN7SL1 | 7 | 31 | INCL_NON_REF_SPLICE |
| 20 | RF00100--RN7SL1 | 5 | 31 | INCL_NON_REF_SPLICE |
| 20 | RF00100--RN7SL1 | 4 | 31 | INCL_NON_REF_SPLICE |
| 20 | RF00100--RN7SL1 | 4 | 31 | INCL_NON_REF_SPLICE |
| 20 | RF00100--RN7SL1 | 4 | 31 | INCL_NON_REF_SPLICE |
| 20 | RF00100--RN7SL1 | 4 | 31 | INCL_NON_REF_SPLICE |
| 20 | RF00100--RN7SL1 | 3 | 31 | INCL_NON_REF_SPLICE |
| 20 | RF00100--RN7SL1 | 3 | 31 | INCL_NON_REF_SPLICE |
| 20 | RF00100--RN7SL1 | 3 | 31 | INCL_NON_REF_SPLICE |
| 20 | RF00100--RN7SL1 | 3 | 31 | INCL_NON_REF_SPLICE |
| 20 | RF00100--RN7SL1 | 3 | 31 | INCL_NON_REF_SPLICE |
| 20 | RF00100--RN7SL1 | 3 | 31 | INCL_NON_REF_SPLICE |
| 20 | RF00100--RN7SL1 | 3 | 31 | INCL_NON_REF_SPLICE |
| 20 | RF00100--RN7SL1 | 3 | 31 | INCL_NON_REF_SPLICE |
| 30 | RF00100--RN7SL2 | 13 | 20 | INCL_NON_REF_SPLICE |
| 30 | RF00100--RN7SL2 | 12 | 20 | INCL_NON_REF_SPLICE |
| 30 | RF00100--RN7SL2 | 11 | 20 | INCL_NON_REF_SPLICE |
| 30 | RF00100--RN7SL2 | 10 | 20 | INCL_NON_REF_SPLICE |
| 30 | RF00100--RN7SL2 | 8 | 20 | INCL_NON_REF_SPLICE |
| 30 | RF00100--RN7SL2 | 8 | 20 | INCL_NON_REF_SPLICE |
| 30 | RF00100--RN7SL2 | 7 | 20 | INCL_NON_REF_SPLICE |
| 30 | RF00100--RN7SL2 | 6 | 20 | INCL_NON_REF_SPLICE |
| 30 | RF00100--RN7SL2 | 5 | 20 | INCL_NON_REF_SPLICE |
| 30 | RF00100--RN7SL2 | 5 | 20 | INCL_NON_REF_SPLICE |
| 30 | RF00100--RN7SL2 | 5 | 20 | INCL_NON_REF_SPLICE |
| 30 | RF00100--RN7SL2 | 5 | 20 | INCL_NON_REF_SPLICE |
| 30 | RF00100--RN7SL2 | 4 | 20 | INCL_NON_REF_SPLICE |
| 30 | RF00100--RN7SL2 | 3 | 20 | INCL_NON_REF_SPLICE |
| 30 | RF00100--RN7SL2 | 3 | 20 | INCL_NON_REF_SPLICE |
| 30 | RF00100--RN7SL2 | 3 | 20 | INCL_NON_REF_SPLICE |
| 30 | RF00100--RN7SL2 | 3 | 20 | INCL_NON_REF_SPLICE |
| 30 | RF00100--RN7SL2 | 3 | 20 | INCL_NON_REF_SPLICE |
| 30 | RF00100--RN7SL2 | 3 | 20 | INCL_NON_REF_SPLICE |
| 30 | RF00100--RN7SL2 | 3 | 20 | INCL_NON_REF_SPLICE |
| 30 | RF00100--RN7SL2 | 3 | 20 | INCL_NON_REF_SPLICE |
| 30 | RF00100--RN7SL2 | 3 | 20 | INCL_NON_REF_SPLICE |
| 49 | RF00100--RN7SL2 | 7 | 75 | INCL_NON_REF_SPLICE |
| 49 | RF00100--RN7SL2 | 6 | 75 | INCL_NON_REF_SPLICE |
| 49 | RF00100--RN7SL2 | 4 | 75 | INCL_NON_REF_SPLICE |
| 49 | RF00100--RN7SL2 | 4 | 75 | INCL_NON_REF_SPLICE |
| 49 | RF00100--RN7SL2 | 4 | 75 | INCL_NON_REF_SPLICE |
| 49 | RF00100--RN7SL2 | 4 | 75 | INCL_NON_REF_SPLICE |
| 49 | RF00100--RN7SL2 | 3 | 75 | INCL_NON_REF_SPLICE |
| 49 | RF00100--RN7SL2 | 3 | 75 | INCL_NON_REF_SPLICE |
| 49 | RF00100--RN7SL2 | 3 | 75 | INCL_NON_REF_SPLICE |
| 49 | RF00100--RN7SL2 | 3 | 75 | INCL_NON_REF_SPLICE |
| 49 | RF00100--RN7SL2 | 3 | 75 | INCL_NON_REF_SPLICE |
| 27 | RF00100--RN7SL2 | 23 | 44 | INCL_NON_REF_SPLICE |
| 27 | RF00100--RN7SL2 | 17 | 44 | INCL_NON_REF_SPLICE |
| 27 | RF00100--RN7SL2 | 15 | 44 | INCL_NON_REF_SPLICE |
| 27 | RF00100--RN7SL2 | 15 | 44 | INCL_NON_REF_SPLICE |
| 27 | RF00100--RN7SL2 | 13 | 44 | INCL_NON_REF_SPLICE |
| 27 | RF00100--RN7SL2 | 13 | 44 | INCL_NON_REF_SPLICE |
| 27 | RF00100--RN7SL2 | 11 | 44 | INCL_NON_REF_SPLICE |
| 27 | RF00100--RN7SL2 | 10 | 44 | INCL_NON_REF_SPLICE |
| 27 | RF00100--RN7SL2 | 9 | 44 | INCL_NON_REF_SPLICE |
| 27 | RF00100--RN7SL2 | 9 | 44 | INCL_NON_REF_SPLICE |
| 27 | RF00100--RN7SL2 | 8 | 44 | INCL_NON_REF_SPLICE |
| 27 | RF00100--RN7SL2 | 8 | 44 | INCL_NON_REF_SPLICE |
| 27 | RF00100--RN7SL2 | 8 | 44 | INCL_NON_REF_SPLICE |
| 27 | RF00100--RN7SL2 | 8 | 44 | INCL_NON_REF_SPLICE |
| 27 | RF00100--RN7SL2 | 8 | 44 | INCL_NON_REF_SPLICE |
| 27 | RF00100--RN7SL2 | 7 | 44 | INCL_NON_REF_SPLICE |
| 27 | RF00100--RN7SL2 | 7 | 44 | INCL_NON_REF_SPLICE |
| 27 | RF00100--RN7SL2 | 7 | 44 | INCL_NON_REF_SPLICE |
| 27 | RF00100--RN7SL2 | 6 | 44 | INCL_NON_REF_SPLICE |
| 27 | RF00100--RN7SL2 | 6 | 44 | INCL_NON_REF_SPLICE |
| 27 | RF00100--RN7SL2 | 6 | 44 | INCL_NON_REF_SPLICE |
| 27 | RF00100--RN7SL2 | 6 | 44 | INCL_NON_REF_SPLICE |
| 27 | RF00100--RN7SL2 | 6 | 44 | INCL_NON_REF_SPLICE |
| 27 | RF00100--RN7SL2 | 5 | 44 | INCL_NON_REF_SPLICE |
| 27 | RF00100--RN7SL2 | 5 | 44 | INCL_NON_REF_SPLICE |
| 27 | RF00100--RN7SL2 | 5 | 44 | INCL_NON_REF_SPLICE |
| 27 | RF00100--RN7SL2 | 5 | 44 | INCL_NON_REF_SPLICE |
| 27 | RF00100--RN7SL2 | 5 | 44 | INCL_NON_REF_SPLICE |
| 27 | RF00100--RN7SL2 | 5 | 44 | INCL_NON_REF_SPLICE |
| 27 | RF00100--RN7SL2 | 5 | 44 | INCL_NON_REF_SPLICE |
| 27 | RF00100--RN7SL2 | 4 | 44 | INCL_NON_REF_SPLICE |
| 27 | RF00100--RN7SL2 | 4 | 44 | INCL_NON_REF_SPLICE |
| 27 | RF00100--RN7SL2 | 4 | 44 | INCL_NON_REF_SPLICE |
| 27 | RF00100--RN7SL2 | 4 | 44 | INCL_NON_REF_SPLICE |
| 27 | RF00100--RN7SL2 | 4 | 44 | INCL_NON_REF_SPLICE |
| 27 | RF00100--RN7SL2 | 4 | 44 | INCL_NON_REF_SPLICE |
| 27 | RF00100--RN7SL2 | 4 | 44 | INCL_NON_REF_SPLICE |
| 27 | RF00100--RN7SL2 | 4 | 44 | INCL_NON_REF_SPLICE |
| 27 | RF00100--RN7SL2 | 4 | 44 | INCL_NON_REF_SPLICE |
| 27 | RF00100--RN7SL2 | 4 | 44 | INCL_NON_REF_SPLICE |
| 27 | RF00100--RN7SL2 | 4 | 44 | INCL_NON_REF_SPLICE |
| 27 | RF00100--RN7SL2 | 4 | 44 | INCL_NON_REF_SPLICE |
| 27 | RF00100--RN7SL2 | 3 | 44 | INCL_NON_REF_SPLICE |
| 27 | RF00100--RN7SL2 | 3 | 44 | INCL_NON_REF_SPLICE |
| 27 | RF00100--RN7SL2 | 3 | 44 | INCL_NON_REF_SPLICE |
| 27 | RF00100--RN7SL2 | 3 | 44 | INCL_NON_REF_SPLICE |
| 27 | RF00100--RN7SL2 | 3 | 44 | INCL_NON_REF_SPLICE |
| 27 | RF00100--RN7SL2 | 3 | 44 | INCL_NON_REF_SPLICE |
| 27 | RF00100--RN7SL2 | 3 | 44 | INCL_NON_REF_SPLICE |
| 27 | RF00100--RN7SL2 | 3 | 44 | INCL_NON_REF_SPLICE |
| 27 | RF00100--RN7SL2 | 3 | 44 | INCL_NON_REF_SPLICE |
| 27 | RF00100--RN7SL2 | 3 | 44 | INCL_NON_REF_SPLICE |
| 27 | RF00100--RN7SL2 | 3 | 44 | INCL_NON_REF_SPLICE |
| 27 | RF00100--RN7SL2 | 3 | 44 | INCL_NON_REF_SPLICE |
| 27 | RF00100--RN7SL2 | 3 | 44 | INCL_NON_REF_SPLICE |
| 27 | RF00100--RN7SL2 | 3 | 44 | INCL_NON_REF_SPLICE |
| 27 | RF00100--RN7SL2 | 3 | 44 | INCL_NON_REF_SPLICE |
| 27 | RF00100--RN7SL2 | 3 | 44 | INCL_NON_REF_SPLICE |
| 27 | RF00100--RN7SL2 | 3 | 44 | INCL_NON_REF_SPLICE |
| 27 | RF00100--RN7SL2 | 3 | 44 | INCL_NON_REF_SPLICE |
| 27 | RF00100--RN7SL2 | 3 | 44 | INCL_NON_REF_SPLICE |
| 27 | RF00100--RN7SL2 | 3 | 44 | INCL_NON_REF_SPLICE |
| 27 | RF00100--RN7SL2 | 3 | 44 | INCL_NON_REF_SPLICE |
| 27 | RF00100--RN7SL2 | 3 | 44 | INCL_NON_REF_SPLICE |
| 27 | RF00100--RN7SL2 | 3 | 44 | INCL_NON_REF_SPLICE |
| 27 | RF00100--RN7SL2 | 3 | 44 | INCL_NON_REF_SPLICE |
| 27 | RF00100--RN7SL2 | 3 | 44 | INCL_NON_REF_SPLICE |
| 27 | RF00100--RN7SL2 | 3 | 44 | INCL_NON_REF_SPLICE |
| 27 | RF00100--RN7SL2 | 3 | 44 | INCL_NON_REF_SPLICE |
| 49 | RF00100--RNA5-8SN2 | 4 | 1 | INCL_NON_REF_SPLICE |
| 49 | RF00100--RNA5-8SN2 | 4 | 1 | INCL_NON_REF_SPLICE |
| 49 | RF00100--RNA5-8SN2 | 4 | 1 | INCL_NON_REF_SPLICE |
| 49 | RF00100--RNA5-8SN2 | 4 | 1 | INCL_NON_REF_SPLICE |
| 27 | RF00100--RNU1-27P | 47 | 0 | INCL_NON_REF_SPLICE |
| 27 | RF00100--RNU1-27P | 31 | 0 | INCL_NON_REF_SPLICE |
| 27 | RF00100--RNU4-2 | 31 | 0 | INCL_NON_REF_SPLICE |
| 34 | RF00100--RPPH1 | 6 | 64 | INCL_NON_REF_SPLICE |
| 34 | RF00100--RPPH1 | 6 | 64 | INCL_NON_REF_SPLICE |
| 34 | RF00100--RPPH1 | 3 | 64 | INCL_NON_REF_SPLICE |
| 34 | RF00100--RPPH1 | 3 | 64 | INCL_NON_REF_SPLICE |
| 27 | RF00100--RPPH1 | 13 | 52 | INCL_NON_REF_SPLICE |
| 27 | RF00100--RPPH1 | 8 | 52 | INCL_NON_REF_SPLICE |
| 27 | RF00100--RPPH1 | 7 | 52 | INCL_NON_REF_SPLICE |
| 27 | RF00100--RPPH1 | 6 | 52 | INCL_NON_REF_SPLICE |
| 27 | RF00100--RPPH1 | 6 | 52 | INCL_NON_REF_SPLICE |
| 27 | RF00100--RPPH1 | 5 | 52 | INCL_NON_REF_SPLICE |
| 27 | RF00100--RPPH1 | 5 | 52 | INCL_NON_REF_SPLICE |
| 27 | RF00100--RPPH1 | 5 | 52 | INCL_NON_REF_SPLICE |
| 27 | RF00100--RPPH1 | 4 | 52 | INCL_NON_REF_SPLICE |
| 27 | RF00100--RPPH1 | 4 | 52 | INCL_NON_REF_SPLICE |
| 27 | RF00100--RPPH1 | 4 | 52 | INCL_NON_REF_SPLICE |
| 27 | RF00100--RPPH1 | 4 | 52 | INCL_NON_REF_SPLICE |
| 27 | RF00100--RPPH1 | 4 | 52 | INCL_NON_REF_SPLICE |
| 27 | RF00100--RPPH1 | 4 | 52 | INCL_NON_REF_SPLICE |
| 27 | RF00100--RPPH1 | 4 | 52 | INCL_NON_REF_SPLICE |
| 27 | RF00100--RPPH1 | 3 | 52 | INCL_NON_REF_SPLICE |
| 27 | RF00100--RPPH1 | 3 | 52 | INCL_NON_REF_SPLICE |
| 27 | RF00100--RPPH1 | 3 | 52 | INCL_NON_REF_SPLICE |
| 27 | RF00100--RPPH1 | 3 | 52 | INCL_NON_REF_SPLICE |
| 27 | RF00100--RPPH1 | 3 | 52 | INCL_NON_REF_SPLICE |
| 27 | RF00100--RPPH1 | 3 | 52 | INCL_NON_REF_SPLICE |
| 27 | RF00100--RPPH1 | 3 | 52 | INCL_NON_REF_SPLICE |
| 27 | RF00100--RPPH1 | 3 | 52 | INCL_NON_REF_SPLICE |
| 27 | RF00100--RPPH1 | 3 | 52 | INCL_NON_REF_SPLICE |
| 27 | RF00100--RPPH1 | 3 | 52 | INCL_NON_REF_SPLICE |
| 27 | RF00100--RPPH1 | 3 | 52 | INCL_NON_REF_SPLICE |
| 27 | RF00100--RPPH1 | 3 | 52 | INCL_NON_REF_SPLICE |
| 34 | RF00100--SNORA73B | 30 | 38 | INCL_NON_REF_SPLICE |
| 34 | RF00100--SNORA73B | 26 | 38 | INCL_NON_REF_SPLICE |
| 34 | RF00100--SNORA73B | 17 | 38 | INCL_NON_REF_SPLICE |
| 34 | RF00100--SNORA73B | 11 | 38 | INCL_NON_REF_SPLICE |
| 34 | RF00100--SNORA73B | 7 | 38 | INCL_NON_REF_SPLICE |
| 34 | RF00100--SNORA73B | 6 | 38 | INCL_NON_REF_SPLICE |
| 34 | RF00100--SNORA73B | 6 | 38 | INCL_NON_REF_SPLICE |
| 34 | RF00100--SNORA73B | 5 | 38 | INCL_NON_REF_SPLICE |
| 34 | RF00100--SNORA73B | 4 | 38 | INCL_NON_REF_SPLICE |
| 34 | RF00100--SNORA73B | 4 | 38 | INCL_NON_REF_SPLICE |
| 34 | RF00100--SNORA73B | 4 | 38 | INCL_NON_REF_SPLICE |
| 34 | RF00100--SNORA73B | 3 | 38 | INCL_NON_REF_SPLICE |
| 27 | RF00100--SNORD3A | 25 | 1 | INCL_NON_REF_SPLICE |
| 55 | RFWD3--ACTR2 | 8 | 0 | INCL_NON_REF_SPLICE |
| 49 | RMRP--AL139099.4 | 3 | 85 | INCL_NON_REF_SPLICE |
| 20 | RMRP--AL139099.4 | 5 | 25 | INCL_NON_REF_SPLICE |
| 20 | RMRP--AL139099.4 | 4 | 25 | INCL_NON_REF_SPLICE |
| 20 | RMRP--AL139099.4 | 4 | 25 | INCL_NON_REF_SPLICE |
| 20 | RMRP--AL139099.4 | 4 | 25 | INCL_NON_REF_SPLICE |
| 20 | RMRP--AL139099.4 | 3 | 25 | INCL_NON_REF_SPLICE |
| 20 | RMRP--AL139099.4 | 3 | 25 | INCL_NON_REF_SPLICE |
| 20 | RMRP--AL139099.4 | 3 | 25 | INCL_NON_REF_SPLICE |
| 20 | RMRP--AL139099.4 | 3 | 25 | INCL_NON_REF_SPLICE |
| 20 | RMRP--AL139099.4 | 3 | 25 | INCL_NON_REF_SPLICE |
| 20 | RMRP--AL139099.4 | 3 | 25 | INCL_NON_REF_SPLICE |
| 20 | RMRP--AL139099.4 | 3 | 25 | INCL_NON_REF_SPLICE |
| 34 | RMRP--PLCG2 | 7 | 46 | INCL_NON_REF_SPLICE |
| 34 | RMRP--PLCG2 | 7 | 46 | INCL_NON_REF_SPLICE |
| 34 | RMRP--PLCG2 | 6 | 46 | INCL_NON_REF_SPLICE |
| 34 | RMRP--PLCG2 | 4 | 46 | INCL_NON_REF_SPLICE |
| 34 | RMRP--PLCG2 | 4 | 46 | INCL_NON_REF_SPLICE |
| 34 | RMRP--PLCG2 | 3 | 46 | INCL_NON_REF_SPLICE |
| 34 | RMRP--PLCG2 | 3 | 46 | INCL_NON_REF_SPLICE |
| 34 | RMRP--PLCG2 | 3 | 46 | INCL_NON_REF_SPLICE |
| 34 | RMRP--PLCG2 | 3 | 46 | INCL_NON_REF_SPLICE |
| 27 | RMRP--PLCG2 | 14 | 21 | INCL_NON_REF_SPLICE |
| 27 | RMRP--PLCG2 | 6 | 21 | INCL_NON_REF_SPLICE |
| 27 | RMRP--PLCG2 | 6 | 21 | INCL_NON_REF_SPLICE |
| 27 | RMRP--PLCG2 | 5 | 21 | INCL_NON_REF_SPLICE |
| 27 | RMRP--PLCG2 | 5 | 21 | INCL_NON_REF_SPLICE |
| 27 | RMRP--PLCG2 | 5 | 21 | INCL_NON_REF_SPLICE |
| 27 | RMRP--PLCG2 | 4 | 21 | INCL_NON_REF_SPLICE |
| 27 | RMRP--PLCG2 | 4 | 21 | INCL_NON_REF_SPLICE |
| 27 | RMRP--PLCG2 | 3 | 21 | INCL_NON_REF_SPLICE |
| 27 | RMRP--PLCG2 | 3 | 21 | INCL_NON_REF_SPLICE |
| 27 | RMRP--PLCG2 | 3 | 21 | INCL_NON_REF_SPLICE |
| 27 | RMRP--PLCG2 | 3 | 21 | INCL_NON_REF_SPLICE |
| 27 | RMRP--PLCG2 | 3 | 21 | INCL_NON_REF_SPLICE |
| 27 | RMRP--PLCG2 | 3 | 21 | INCL_NON_REF_SPLICE |
| 27 | RMRP--PLCG2 | 3 | 21 | INCL_NON_REF_SPLICE |
| 27 | RMRP--PLCG2 | 3 | 21 | INCL_NON_REF_SPLICE |
| 27 | RMRP--PLCG2 | 3 | 21 | INCL_NON_REF_SPLICE |
| 34 | RMRP--RF00100 | 10 | 109 | INCL_NON_REF_SPLICE |
| 34 | RMRP--RF00100 | 9 | 109 | INCL_NON_REF_SPLICE |
| 34 | RMRP--RF00100 | 8 | 109 | INCL_NON_REF_SPLICE |
| 34 | RMRP--RF00100 | 7 | 109 | INCL_NON_REF_SPLICE |
| 34 | RMRP--RF00100 | 6 | 109 | INCL_NON_REF_SPLICE |
| 34 | RMRP--RF00100 | 6 | 109 | INCL_NON_REF_SPLICE |
| 34 | RMRP--RF00100 | 5 | 109 | INCL_NON_REF_SPLICE |
| 34 | RMRP--RF00100 | 4 | 109 | INCL_NON_REF_SPLICE |
| 34 | RMRP--RF00100 | 4 | 109 | INCL_NON_REF_SPLICE |
| 34 | RMRP--RF00100 | 4 | 109 | INCL_NON_REF_SPLICE |
| 34 | RMRP--RF00100 | 4 | 109 | INCL_NON_REF_SPLICE |
| 34 | RMRP--RF00100 | 3 | 109 | INCL_NON_REF_SPLICE |
| 34 | RMRP--RF00100 | 3 | 109 | INCL_NON_REF_SPLICE |
| 34 | RMRP--RF00100 | 3 | 109 | INCL_NON_REF_SPLICE |
| 34 | RMRP--RF00100 | 3 | 109 | INCL_NON_REF_SPLICE |
| 34 | RMRP--RF00100 | 3 | 109 | INCL_NON_REF_SPLICE |
| 34 | RMRP--RF00100 | 3 | 109 | INCL_NON_REF_SPLICE |
| 34 | RMRP--RF00100 | 3 | 109 | INCL_NON_REF_SPLICE |
| 34 | RMRP--RF00100 | 3 | 109 | INCL_NON_REF_SPLICE |
| 34 | RMRP--RF00100 | 3 | 109 | INCL_NON_REF_SPLICE |
| 34 | RMRP--RF00100 | 3 | 109 | INCL_NON_REF_SPLICE |
| 34 | RMRP--RF00100 | 3 | 109 | INCL_NON_REF_SPLICE |
| 34 | RMRP--RF00100 | 3 | 109 | INCL_NON_REF_SPLICE |
| 27 | RMRP--RF00100 | 15 | 37 | INCL_NON_REF_SPLICE |
| 27 | RMRP--RF00100 | 7 | 37 | INCL_NON_REF_SPLICE |
| 27 | RMRP--RF00100 | 6 | 37 | INCL_NON_REF_SPLICE |
| 27 | RMRP--RF00100 | 6 | 37 | INCL_NON_REF_SPLICE |
| 27 | RMRP--RF00100 | 6 | 37 | INCL_NON_REF_SPLICE |
| 27 | RMRP--RF00100 | 6 | 37 | INCL_NON_REF_SPLICE |
| 27 | RMRP--RF00100 | 6 | 37 | INCL_NON_REF_SPLICE |
| 27 | RMRP--RF00100 | 6 | 37 | INCL_NON_REF_SPLICE |
| 27 | RMRP--RF00100 | 5 | 37 | INCL_NON_REF_SPLICE |
| 27 | RMRP--RF00100 | 5 | 37 | INCL_NON_REF_SPLICE |
| 27 | RMRP--RF00100 | 5 | 37 | INCL_NON_REF_SPLICE |
| 27 | RMRP--RF00100 | 5 | 37 | INCL_NON_REF_SPLICE |
| 27 | RMRP--RF00100 | 5 | 37 | INCL_NON_REF_SPLICE |
| 27 | RMRP--RF00100 | 5 | 37 | INCL_NON_REF_SPLICE |
| 27 | RMRP--RF00100 | 5 | 37 | INCL_NON_REF_SPLICE |
| 27 | RMRP--RF00100 | 4 | 37 | INCL_NON_REF_SPLICE |
| 27 | RMRP--RF00100 | 4 | 37 | INCL_NON_REF_SPLICE |
| 27 | RMRP--RF00100 | 4 | 37 | INCL_NON_REF_SPLICE |
| 27 | RMRP--RF00100 | 4 | 37 | INCL_NON_REF_SPLICE |
| 27 | RMRP--RF00100 | 4 | 37 | INCL_NON_REF_SPLICE |
| 27 | RMRP--RF00100 | 4 | 37 | INCL_NON_REF_SPLICE |
| 27 | RMRP--RF00100 | 4 | 37 | INCL_NON_REF_SPLICE |
| 27 | RMRP--RF00100 | 4 | 37 | INCL_NON_REF_SPLICE |
| 27 | RMRP--RF00100 | 4 | 37 | INCL_NON_REF_SPLICE |
| 27 | RMRP--RF00100 | 4 | 37 | INCL_NON_REF_SPLICE |
| 27 | RMRP--RF00100 | 3 | 37 | INCL_NON_REF_SPLICE |
| 27 | RMRP--RF00100 | 3 | 37 | INCL_NON_REF_SPLICE |
| 27 | RMRP--RF00100 | 3 | 37 | INCL_NON_REF_SPLICE |
| 27 | RMRP--RF00100 | 3 | 37 | INCL_NON_REF_SPLICE |
| 27 | RMRP--RF00100 | 3 | 37 | INCL_NON_REF_SPLICE |
| 27 | RMRP--RF00100 | 3 | 37 | INCL_NON_REF_SPLICE |
| 27 | RMRP--RF00100 | 3 | 37 | INCL_NON_REF_SPLICE |
| 27 | RMRP--RF00100 | 3 | 37 | INCL_NON_REF_SPLICE |
| 27 | RMRP--RF00100 | 3 | 37 | INCL_NON_REF_SPLICE |
| 27 | RMRP--RF00100 | 3 | 37 | INCL_NON_REF_SPLICE |
| 27 | RMRP--RF00100 | 3 | 37 | INCL_NON_REF_SPLICE |
| 27 | RMRP--RF00100 | 3 | 37 | INCL_NON_REF_SPLICE |
| 34 | RMRP--RN7SK | 10 | 109 | INCL_NON_REF_SPLICE |
| 34 | RMRP--RN7SK | 9 | 109 | INCL_NON_REF_SPLICE |
| 34 | RMRP--RN7SK | 8 | 109 | INCL_NON_REF_SPLICE |
| 34 | RMRP--RN7SK | 7 | 109 | INCL_NON_REF_SPLICE |
| 34 | RMRP--RN7SK | 6 | 109 | INCL_NON_REF_SPLICE |
| 34 | RMRP--RN7SK | 6 | 109 | INCL_NON_REF_SPLICE |
| 34 | RMRP--RN7SK | 5 | 109 | INCL_NON_REF_SPLICE |
| 34 | RMRP--RN7SK | 4 | 109 | INCL_NON_REF_SPLICE |
| 34 | RMRP--RN7SK | 4 | 109 | INCL_NON_REF_SPLICE |
| 34 | RMRP--RN7SK | 4 | 109 | INCL_NON_REF_SPLICE |
| 34 | RMRP--RN7SK | 4 | 109 | INCL_NON_REF_SPLICE |
| 34 | RMRP--RN7SK | 3 | 109 | INCL_NON_REF_SPLICE |
| 34 | RMRP--RN7SK | 3 | 109 | INCL_NON_REF_SPLICE |
| 34 | RMRP--RN7SK | 3 | 109 | INCL_NON_REF_SPLICE |
| 34 | RMRP--RN7SK | 3 | 109 | INCL_NON_REF_SPLICE |
| 34 | RMRP--RN7SK | 3 | 109 | INCL_NON_REF_SPLICE |
| 34 | RMRP--RN7SK | 3 | 109 | INCL_NON_REF_SPLICE |
| 34 | RMRP--RN7SK | 3 | 109 | INCL_NON_REF_SPLICE |
| 34 | RMRP--RN7SK | 3 | 109 | INCL_NON_REF_SPLICE |
| 34 | RMRP--RN7SK | 3 | 109 | INCL_NON_REF_SPLICE |
| 34 | RMRP--RN7SK | 3 | 109 | INCL_NON_REF_SPLICE |
| 34 | RMRP--RN7SK | 3 | 109 | INCL_NON_REF_SPLICE |
| 34 | RMRP--RN7SK | 3 | 109 | INCL_NON_REF_SPLICE |
| 27 | RMRP--RN7SK | 15 | 37 | INCL_NON_REF_SPLICE |
| 27 | RMRP--RN7SK | 7 | 37 | INCL_NON_REF_SPLICE |
| 27 | RMRP--RN7SK | 6 | 37 | INCL_NON_REF_SPLICE |
| 27 | RMRP--RN7SK | 6 | 37 | INCL_NON_REF_SPLICE |
| 27 | RMRP--RN7SK | 6 | 37 | INCL_NON_REF_SPLICE |
| 27 | RMRP--RN7SK | 6 | 37 | INCL_NON_REF_SPLICE |
| 27 | RMRP--RN7SK | 6 | 37 | INCL_NON_REF_SPLICE |
| 27 | RMRP--RN7SK | 6 | 37 | INCL_NON_REF_SPLICE |
| 27 | RMRP--RN7SK | 5 | 37 | INCL_NON_REF_SPLICE |
| 27 | RMRP--RN7SK | 5 | 37 | INCL_NON_REF_SPLICE |
| 27 | RMRP--RN7SK | 5 | 37 | INCL_NON_REF_SPLICE |
| 27 | RMRP--RN7SK | 5 | 37 | INCL_NON_REF_SPLICE |
| 27 | RMRP--RN7SK | 5 | 37 | INCL_NON_REF_SPLICE |
| 27 | RMRP--RN7SK | 5 | 37 | INCL_NON_REF_SPLICE |
| 27 | RMRP--RN7SK | 5 | 37 | INCL_NON_REF_SPLICE |
| 27 | RMRP--RN7SK | 4 | 37 | INCL_NON_REF_SPLICE |
| 27 | RMRP--RN7SK | 4 | 37 | INCL_NON_REF_SPLICE |
| 27 | RMRP--RN7SK | 4 | 37 | INCL_NON_REF_SPLICE |
| 27 | RMRP--RN7SK | 4 | 37 | INCL_NON_REF_SPLICE |
| 27 | RMRP--RN7SK | 4 | 37 | INCL_NON_REF_SPLICE |
| 27 | RMRP--RN7SK | 4 | 37 | INCL_NON_REF_SPLICE |
| 27 | RMRP--RN7SK | 4 | 37 | INCL_NON_REF_SPLICE |
| 27 | RMRP--RN7SK | 4 | 37 | INCL_NON_REF_SPLICE |
| 27 | RMRP--RN7SK | 4 | 37 | INCL_NON_REF_SPLICE |
| 27 | RMRP--RN7SK | 4 | 37 | INCL_NON_REF_SPLICE |
| 27 | RMRP--RN7SK | 3 | 37 | INCL_NON_REF_SPLICE |
| 27 | RMRP--RN7SK | 3 | 37 | INCL_NON_REF_SPLICE |
| 27 | RMRP--RN7SK | 3 | 37 | INCL_NON_REF_SPLICE |
| 27 | RMRP--RN7SK | 3 | 37 | INCL_NON_REF_SPLICE |
| 27 | RMRP--RN7SK | 3 | 37 | INCL_NON_REF_SPLICE |
| 27 | RMRP--RN7SK | 3 | 37 | INCL_NON_REF_SPLICE |
| 27 | RMRP--RN7SK | 3 | 37 | INCL_NON_REF_SPLICE |
| 27 | RMRP--RN7SK | 3 | 37 | INCL_NON_REF_SPLICE |
| 27 | RMRP--RN7SK | 3 | 37 | INCL_NON_REF_SPLICE |
| 27 | RMRP--RN7SK | 3 | 37 | INCL_NON_REF_SPLICE |
| 27 | RMRP--RN7SK | 3 | 37 | INCL_NON_REF_SPLICE |
| 27 | RMRP--RN7SK | 3 | 37 | INCL_NON_REF_SPLICE |
| 49 | RMRP--RN7SL1 | 3 | 85 | INCL_NON_REF_SPLICE |
| 20 | RMRP--RN7SL1 | 5 | 25 | INCL_NON_REF_SPLICE |
| 20 | RMRP--RN7SL1 | 4 | 25 | INCL_NON_REF_SPLICE |
| 20 | RMRP--RN7SL1 | 4 | 25 | INCL_NON_REF_SPLICE |
| 20 | RMRP--RN7SL1 | 4 | 25 | INCL_NON_REF_SPLICE |
| 20 | RMRP--RN7SL1 | 3 | 25 | INCL_NON_REF_SPLICE |
| 20 | RMRP--RN7SL1 | 3 | 25 | INCL_NON_REF_SPLICE |
| 20 | RMRP--RN7SL1 | 3 | 25 | INCL_NON_REF_SPLICE |
| 20 | RMRP--RN7SL1 | 3 | 25 | INCL_NON_REF_SPLICE |
| 20 | RMRP--RN7SL1 | 3 | 25 | INCL_NON_REF_SPLICE |
| 20 | RMRP--RN7SL1 | 3 | 25 | INCL_NON_REF_SPLICE |
| 20 | RMRP--RN7SL1 | 3 | 25 | INCL_NON_REF_SPLICE |
| 30 | RMRP--RN7SL2 | 6 | 10 | INCL_NON_REF_SPLICE |
| 30 | RMRP--RN7SL2 | 3 | 10 | INCL_NON_REF_SPLICE |
| 34 | RMRP--RN7SL2 | 10 | 172 | INCL_NON_REF_SPLICE |
| 34 | RMRP--RN7SL2 | 8 | 171 | INCL_NON_REF_SPLICE |
| 34 | RMRP--RN7SL2 | 7 | 171 | INCL_NON_REF_SPLICE |
| 34 | RMRP--RN7SL2 | 6 | 171 | INCL_NON_REF_SPLICE |
| 34 | RMRP--RN7SL2 | 4 | 171 | INCL_NON_REF_SPLICE |
| 34 | RMRP--RN7SL2 | 3 | 171 | INCL_NON_REF_SPLICE |
| 34 | RMRP--RN7SL2 | 5 | 155 | INCL_NON_REF_SPLICE |
| 34 | RMRP--RN7SL2 | 8 | 39 | INCL_NON_REF_SPLICE |
| 34 | RMRP--RN7SL2 | 8 | 39 | INCL_NON_REF_SPLICE |
| 34 | RMRP--RN7SL2 | 8 | 39 | INCL_NON_REF_SPLICE |
| 34 | RMRP--RN7SL2 | 7 | 39 | INCL_NON_REF_SPLICE |
| 27 | RMRP--RN7SL2 | 15 | 55 | INCL_NON_REF_SPLICE |
| 27 | RMRP--RN7SL2 | 14 | 55 | INCL_NON_REF_SPLICE |
| 27 | RMRP--RN7SL2 | 10 | 55 | INCL_NON_REF_SPLICE |
| 27 | RMRP--RN7SL2 | 8 | 55 | INCL_NON_REF_SPLICE |
| 27 | RMRP--RN7SL2 | 8 | 55 | INCL_NON_REF_SPLICE |
| 27 | RMRP--RN7SL2 | 7 | 55 | INCL_NON_REF_SPLICE |
| 27 | RMRP--RN7SL2 | 7 | 55 | INCL_NON_REF_SPLICE |
| 27 | RMRP--RN7SL2 | 7 | 55 | INCL_NON_REF_SPLICE |
| 27 | RMRP--RN7SL2 | 6 | 55 | INCL_NON_REF_SPLICE |
| 27 | RMRP--RN7SL2 | 6 | 55 | INCL_NON_REF_SPLICE |
| 27 | RMRP--RN7SL2 | 5 | 55 | INCL_NON_REF_SPLICE |
| 27 | RMRP--RN7SL2 | 5 | 55 | INCL_NON_REF_SPLICE |
| 27 | RMRP--RN7SL2 | 5 | 55 | INCL_NON_REF_SPLICE |
| 27 | RMRP--RN7SL2 | 5 | 55 | INCL_NON_REF_SPLICE |
| 27 | RMRP--RN7SL2 | 4 | 55 | INCL_NON_REF_SPLICE |
| 27 | RMRP--RN7SL2 | 4 | 55 | INCL_NON_REF_SPLICE |
| 27 | RMRP--RN7SL2 | 4 | 55 | INCL_NON_REF_SPLICE |
| 27 | RMRP--RN7SL2 | 4 | 55 | INCL_NON_REF_SPLICE |
| 27 | RMRP--RN7SL2 | 4 | 55 | INCL_NON_REF_SPLICE |
| 27 | RMRP--RN7SL2 | 4 | 55 | INCL_NON_REF_SPLICE |
| 27 | RMRP--RN7SL2 | 4 | 55 | INCL_NON_REF_SPLICE |
| 27 | RMRP--RN7SL2 | 4 | 55 | INCL_NON_REF_SPLICE |
| 27 | RMRP--RN7SL2 | 6 | 46 | INCL_NON_REF_SPLICE |
| 27 | RMRP--RN7SL2 | 3 | 55 | INCL_NON_REF_SPLICE |
| 27 | RMRP--RN7SL2 | 3 | 55 | INCL_NON_REF_SPLICE |
| 27 | RMRP--RN7SL2 | 3 | 55 | INCL_NON_REF_SPLICE |
| 27 | RMRP--RN7SL2 | 3 | 55 | INCL_NON_REF_SPLICE |
| 27 | RMRP--RN7SL2 | 3 | 55 | INCL_NON_REF_SPLICE |
| 27 | RMRP--RN7SL2 | 3 | 55 | INCL_NON_REF_SPLICE |
| 27 | RMRP--RN7SL2 | 3 | 55 | INCL_NON_REF_SPLICE |
| 27 | RMRP--RN7SL2 | 3 | 55 | INCL_NON_REF_SPLICE |
| 27 | RMRP--RN7SL2 | 3 | 55 | INCL_NON_REF_SPLICE |
| 27 | RMRP--RN7SL2 | 3 | 55 | INCL_NON_REF_SPLICE |
| 27 | RMRP--RN7SL2 | 3 | 46 | INCL_NON_REF_SPLICE |
| 49 | RMRP--RNA5-8SN2 | 3 | 7 | INCL_NON_REF_SPLICE |
| 49 | RMRP--RNA5-8SN2 | 3 | 2 | INCL_NON_REF_SPLICE |
| 27 | RMRP--RNU1-28P | 34 | 0 | INCL_NON_REF_SPLICE |
| 34 | RN7SK--AL139099.4 | 15 | 102 | INCL_NON_REF_SPLICE |
| 34 | RN7SK--AL139099.4 | 11 | 102 | INCL_NON_REF_SPLICE |
| 34 | RN7SK--AL139099.4 | 11 | 102 | INCL_NON_REF_SPLICE |
| 34 | RN7SK--AL139099.4 | 9 | 102 | INCL_NON_REF_SPLICE |
| 34 | RN7SK--AL139099.4 | 9 | 102 | INCL_NON_REF_SPLICE |
| 34 | RN7SK--AL139099.4 | 8 | 102 | INCL_NON_REF_SPLICE |
| 34 | RN7SK--AL139099.4 | 8 | 102 | INCL_NON_REF_SPLICE |
| 34 | RN7SK--AL139099.4 | 7 | 102 | INCL_NON_REF_SPLICE |
| 34 | RN7SK--AL139099.4 | 6 | 102 | INCL_NON_REF_SPLICE |
| 34 | RN7SK--AL139099.4 | 6 | 102 | INCL_NON_REF_SPLICE |
| 34 | RN7SK--AL139099.4 | 5 | 102 | INCL_NON_REF_SPLICE |
| 34 | RN7SK--AL139099.4 | 5 | 102 | INCL_NON_REF_SPLICE |
| 34 | RN7SK--AL139099.4 | 4 | 102 | INCL_NON_REF_SPLICE |
| 34 | RN7SK--AL139099.4 | 4 | 102 | INCL_NON_REF_SPLICE |
| 34 | RN7SK--AL139099.4 | 4 | 102 | INCL_NON_REF_SPLICE |
| 34 | RN7SK--AL139099.4 | 3 | 102 | INCL_NON_REF_SPLICE |
| 34 | RN7SK--AL139099.4 | 3 | 102 | INCL_NON_REF_SPLICE |
| 34 | RN7SK--AL139099.4 | 3 | 102 | INCL_NON_REF_SPLICE |
| 34 | RN7SK--AL139099.4 | 3 | 102 | INCL_NON_REF_SPLICE |
| 34 | RN7SK--AL139099.4 | 3 | 102 | INCL_NON_REF_SPLICE |
| 34 | RN7SK--AL139099.4 | 3 | 102 | INCL_NON_REF_SPLICE |
| 34 | RN7SK--AL139099.4 | 3 | 102 | INCL_NON_REF_SPLICE |
| 34 | RN7SK--AL139099.4 | 3 | 102 | INCL_NON_REF_SPLICE |
| 20 | RN7SK--AL139099.4 | 12 | 31 | INCL_NON_REF_SPLICE |
| 20 | RN7SK--AL139099.4 | 7 | 31 | INCL_NON_REF_SPLICE |
| 20 | RN7SK--AL139099.4 | 5 | 31 | INCL_NON_REF_SPLICE |
| 20 | RN7SK--AL139099.4 | 4 | 31 | INCL_NON_REF_SPLICE |
| 20 | RN7SK--AL139099.4 | 4 | 31 | INCL_NON_REF_SPLICE |
| 20 | RN7SK--AL139099.4 | 4 | 31 | INCL_NON_REF_SPLICE |
| 20 | RN7SK--AL139099.4 | 4 | 31 | INCL_NON_REF_SPLICE |
| 20 | RN7SK--AL139099.4 | 3 | 31 | INCL_NON_REF_SPLICE |
| 20 | RN7SK--AL139099.4 | 3 | 31 | INCL_NON_REF_SPLICE |
| 20 | RN7SK--AL139099.4 | 3 | 31 | INCL_NON_REF_SPLICE |
| 20 | RN7SK--AL139099.4 | 3 | 31 | INCL_NON_REF_SPLICE |
| 20 | RN7SK--AL139099.4 | 3 | 31 | INCL_NON_REF_SPLICE |
| 20 | RN7SK--AL139099.4 | 3 | 31 | INCL_NON_REF_SPLICE |
| 20 | RN7SK--AL139099.4 | 3 | 31 | INCL_NON_REF_SPLICE |
| 20 | RN7SK--AL139099.4 | 3 | 31 | INCL_NON_REF_SPLICE |
| 34 | RN7SK--AL355075.4 | 6 | 64 | INCL_NON_REF_SPLICE |
| 34 | RN7SK--AL355075.4 | 6 | 64 | INCL_NON_REF_SPLICE |
| 34 | RN7SK--AL355075.4 | 3 | 64 | INCL_NON_REF_SPLICE |
| 34 | RN7SK--AL355075.4 | 3 | 64 | INCL_NON_REF_SPLICE |
| 27 | RN7SK--AL355075.4 | 13 | 52 | INCL_NON_REF_SPLICE |
| 27 | RN7SK--AL355075.4 | 8 | 52 | INCL_NON_REF_SPLICE |
| 27 | RN7SK--AL355075.4 | 7 | 52 | INCL_NON_REF_SPLICE |
| 27 | RN7SK--AL355075.4 | 6 | 52 | INCL_NON_REF_SPLICE |
| 27 | RN7SK--AL355075.4 | 6 | 52 | INCL_NON_REF_SPLICE |
| 27 | RN7SK--AL355075.4 | 5 | 52 | INCL_NON_REF_SPLICE |
| 27 | RN7SK--AL355075.4 | 5 | 52 | INCL_NON_REF_SPLICE |
| 27 | RN7SK--AL355075.4 | 5 | 52 | INCL_NON_REF_SPLICE |
| 27 | RN7SK--AL355075.4 | 4 | 52 | INCL_NON_REF_SPLICE |
| 27 | RN7SK--AL355075.4 | 4 | 52 | INCL_NON_REF_SPLICE |
| 27 | RN7SK--AL355075.4 | 4 | 52 | INCL_NON_REF_SPLICE |
| 27 | RN7SK--AL355075.4 | 4 | 52 | INCL_NON_REF_SPLICE |
| 27 | RN7SK--AL355075.4 | 4 | 52 | INCL_NON_REF_SPLICE |
| 27 | RN7SK--AL355075.4 | 4 | 52 | INCL_NON_REF_SPLICE |
| 27 | RN7SK--AL355075.4 | 4 | 52 | INCL_NON_REF_SPLICE |
| 27 | RN7SK--AL355075.4 | 3 | 52 | INCL_NON_REF_SPLICE |
| 27 | RN7SK--AL355075.4 | 3 | 52 | INCL_NON_REF_SPLICE |
| 27 | RN7SK--AL355075.4 | 3 | 52 | INCL_NON_REF_SPLICE |
| 27 | RN7SK--AL355075.4 | 3 | 52 | INCL_NON_REF_SPLICE |
| 27 | RN7SK--AL355075.4 | 3 | 52 | INCL_NON_REF_SPLICE |
| 27 | RN7SK--AL355075.4 | 3 | 52 | INCL_NON_REF_SPLICE |
| 27 | RN7SK--AL355075.4 | 3 | 52 | INCL_NON_REF_SPLICE |
| 27 | RN7SK--AL355075.4 | 3 | 52 | INCL_NON_REF_SPLICE |
| 27 | RN7SK--AL355075.4 | 3 | 52 | INCL_NON_REF_SPLICE |
| 27 | RN7SK--AL355075.4 | 3 | 52 | INCL_NON_REF_SPLICE |
| 27 | RN7SK--AL355075.4 | 3 | 52 | INCL_NON_REF_SPLICE |
| 27 | RN7SK--AL355075.4 | 3 | 52 | INCL_NON_REF_SPLICE |
| 49 | RN7SK--CPNE3 | 4 | 0 | INCL_NON_REF_SPLICE |
| 27 | RN7SKP175--RN7SKP281 | 11 | 0 | INCL_NON_REF_SPLICE |
| 34 | RN7SKP203--RN7SKP174 | 8 | 0 | INCL_NON_REF_SPLICE |
| 30 | RN7SKP22--RN7SKP48 | 5 | 0 | INCL_NON_REF_SPLICE |
| 27 | RN7SKP22--RN7SKP48 | 11 | 0 | INCL_NON_REF_SPLICE |
| 30 | RN7SKP239--RN7SKP203 | 5 | 0 | INCL_NON_REF_SPLICE |
| 27 | RN7SKP239--RN7SKP203 | 15 | 0 | INCL_NON_REF_SPLICE |
| 27 | RN7SKP255--RN7SKP107 | 8 | 0 | INCL_NON_REF_SPLICE |
| 20 | RN7SKP255--RN7SKP156 | 6 | 0 | INCL_NON_REF_SPLICE |
| 27 | RN7SKP255--SNHG3 | 14 | 4 | INCL_NON_REF_SPLICE |
| 27 | RN7SKP255--SNHG3 | 7 | 4 | INCL_NON_REF_SPLICE |
| 27 | RN7SKP255--SNHG3 | 4 | 4 | INCL_NON_REF_SPLICE |
| 27 | RN7SKP255--SNHG3 | 3 | 4 | INCL_NON_REF_SPLICE |
| 27 | RN7SKP255--SNHG3 | 3 | 4 | INCL_NON_REF_SPLICE |
| 27 | RN7SKP255--SNORA73A | 14 | 4 | INCL_NON_REF_SPLICE |
| 27 | RN7SKP255--SNORA73A | 7 | 4 | INCL_NON_REF_SPLICE |
| 27 | RN7SKP255--SNORA73A | 4 | 4 | INCL_NON_REF_SPLICE |
| 27 | RN7SKP255--SNORA73A | 3 | 4 | INCL_NON_REF_SPLICE |
| 27 | RN7SKP255--SNORA73A | 3 | 4 | INCL_NON_REF_SPLICE |
| 27 | RN7SKP261--RN7SKP255 | 6 | 0 | INCL_NON_REF_SPLICE |
| 49 | RN7SKP290--RF00100 | 3 | 1 | INCL_NON_REF_SPLICE |
| 49 | RN7SKP290--RN7SK | 3 | 1 | INCL_NON_REF_SPLICE |
| 27 | RN7SKP45--RN7SKP80 | 25 | 0 | INCL_NON_REF_SPLICE |
| 20 | RN7SKP45--RN7SKP80 | 6 | 0 | INCL_NON_REF_SPLICE |
| 27 | RN7SKP65--RN7SKP80 | 29 | 0 | INCL_NON_REF_SPLICE |
| 20 | RN7SKP65--RN7SKP80 | 6 | 0 | INCL_NON_REF_SPLICE |
| 45 | RN7SKP71--RN7SKP156 | 5 | 0 | INCL_NON_REF_SPLICE |
| 20 | RN7SKP71--RN7SKP185 | 4 | 0 | INCL_NON_REF_SPLICE |
| 30 | RN7SKP71--RN7SKP48 | 189 | 0 | INCL_NON_REF_SPLICE |
| 46 | RN7SKP71--RN7SKP48 | 53 | 9 | INCL_NON_REF_SPLICE |
| 49 | RN7SKP71--RN7SKP48 | 41 | 37 | INCL_NON_REF_SPLICE |
| 51 | RN7SKP71--RN7SKP48 | 29 | 4 | INCL_NON_REF_SPLICE |
| 47 | RN7SKP71--RN7SKP48 | 60 | 14 | INCL_NON_REF_SPLICE |
| 34 | RN7SKP71--RN7SKP48 | 91 | 0 | INCL_NON_REF_SPLICE |
| 34 | RN7SKP71--RN7SKP48 | 10 | 0 | INCL_NON_REF_SPLICE |
| 27 | RN7SKP71--RN7SKP48 | 395 | 0 | INCL_NON_REF_SPLICE |
| 48 | RN7SKP71--RN7SKP48 | 51 | 13 | INCL_NON_REF_SPLICE |
| 20 | RN7SKP71--RN7SKP48 | 79 | 0 | INCL_NON_REF_SPLICE |
| 45 | RN7SKP71--RN7SKP48 | 99 | 20 | INCL_NON_REF_SPLICE |
| 30 | RN7SKP80--RN7SKP118 | 151 | 0 | INCL_NON_REF_SPLICE |
| 34 | RN7SKP80--RN7SKP118 | 68 | 0 | INCL_NON_REF_SPLICE |
| 20 | RN7SKP80--RN7SKP118 | 74 | 0 | INCL_NON_REF_SPLICE |
| 30 | RN7SKP80--RN7SKP62 | 8 | 0 | INCL_NON_REF_SPLICE |
| 34 | RN7SKP80--RN7SKP62 | 9 | 0 | INCL_NON_REF_SPLICE |
| 27 | RN7SKP80--RN7SKP62 | 14 | 0 | INCL_NON_REF_SPLICE |
| 20 | RN7SKP80--RN7SKP62 | 6 | 0 | INCL_NON_REF_SPLICE |
| 27 | RN7SKP80--ZNF277 | 12 | 0 | INCL_NON_REF_SPLICE |
| 30 | RN7SKP90--RN7SKP281 | 7 | 0 | INCL_NON_REF_SPLICE |
| 30 | RN7SKP91--RN7SKP80 | 12 | 0 | INCL_NON_REF_SPLICE |
| 49 | RN7SKP91--RN7SKP80 | 4 | 0 | INCL_NON_REF_SPLICE |
| 51 | RN7SKP91--RN7SKP80 | 7 | 0 | INCL_NON_REF_SPLICE |
| 34 | RN7SKP91--RN7SKP80 | 7 | 0 | INCL_NON_REF_SPLICE |
| 45 | RN7SKP91--RN7SKP80 | 5 | 0 | INCL_NON_REF_SPLICE |
| 20 | RN7SK--RF00003 | 10 | 0 | INCL_NON_REF_SPLICE |
| 34 | RN7SK--RMRP | 18 | 184 | INCL_NON_REF_SPLICE |
| 34 | RN7SK--RMRP | 14 | 184 | INCL_NON_REF_SPLICE |
| 34 | RN7SK--RMRP | 11 | 184 | INCL_NON_REF_SPLICE |
| 34 | RN7SK--RMRP | 9 | 184 | INCL_NON_REF_SPLICE |
| 34 | RN7SK--RMRP | 7 | 184 | INCL_NON_REF_SPLICE |
| 34 | RN7SK--RMRP | 6 | 184 | INCL_NON_REF_SPLICE |
| 34 | RN7SK--RMRP | 6 | 184 | INCL_NON_REF_SPLICE |
| 34 | RN7SK--RMRP | 5 | 184 | INCL_NON_REF_SPLICE |
| 34 | RN7SK--RMRP | 5 | 184 | INCL_NON_REF_SPLICE |
| 34 | RN7SK--RMRP | 5 | 184 | INCL_NON_REF_SPLICE |
| 34 | RN7SK--RMRP | 4 | 184 | INCL_NON_REF_SPLICE |
| 34 | RN7SK--RMRP | 4 | 184 | INCL_NON_REF_SPLICE |
| 34 | RN7SK--RMRP | 4 | 184 | INCL_NON_REF_SPLICE |
| 34 | RN7SK--RMRP | 4 | 184 | INCL_NON_REF_SPLICE |
| 34 | RN7SK--RMRP | 4 | 184 | INCL_NON_REF_SPLICE |
| 34 | RN7SK--RMRP | 4 | 184 | INCL_NON_REF_SPLICE |
| 34 | RN7SK--RMRP | 4 | 184 | INCL_NON_REF_SPLICE |
| 34 | RN7SK--RMRP | 4 | 184 | INCL_NON_REF_SPLICE |
| 34 | RN7SK--RMRP | 4 | 184 | INCL_NON_REF_SPLICE |
| 34 | RN7SK--RMRP | 4 | 184 | INCL_NON_REF_SPLICE |
| 34 | RN7SK--RMRP | 4 | 184 | INCL_NON_REF_SPLICE |
| 34 | RN7SK--RMRP | 3 | 184 | INCL_NON_REF_SPLICE |
| 34 | RN7SK--RMRP | 3 | 184 | INCL_NON_REF_SPLICE |
| 34 | RN7SK--RMRP | 3 | 184 | INCL_NON_REF_SPLICE |
| 34 | RN7SK--RMRP | 3 | 184 | INCL_NON_REF_SPLICE |
| 34 | RN7SK--RMRP | 3 | 184 | INCL_NON_REF_SPLICE |
| 34 | RN7SK--RMRP | 3 | 184 | INCL_NON_REF_SPLICE |
| 34 | RN7SK--RMRP | 3 | 184 | INCL_NON_REF_SPLICE |
| 34 | RN7SK--RMRP | 3 | 184 | INCL_NON_REF_SPLICE |
| 34 | RN7SK--RMRP | 3 | 184 | INCL_NON_REF_SPLICE |
| 34 | RN7SK--RMRP | 3 | 184 | INCL_NON_REF_SPLICE |
| 34 | RN7SK--RMRP | 3 | 184 | INCL_NON_REF_SPLICE |
| 34 | RN7SK--RMRP | 3 | 184 | INCL_NON_REF_SPLICE |
| 27 | RN7SK--RMRP | 15 | 24 | INCL_NON_REF_SPLICE |
| 27 | RN7SK--RMRP | 12 | 24 | INCL_NON_REF_SPLICE |
| 27 | RN7SK--RMRP | 11 | 24 | INCL_NON_REF_SPLICE |
| 27 | RN7SK--RMRP | 10 | 24 | INCL_NON_REF_SPLICE |
| 27 | RN7SK--RMRP | 10 | 24 | INCL_NON_REF_SPLICE |
| 27 | RN7SK--RMRP | 6 | 24 | INCL_NON_REF_SPLICE |
| 27 | RN7SK--RMRP | 5 | 24 | INCL_NON_REF_SPLICE |
| 27 | RN7SK--RMRP | 5 | 24 | INCL_NON_REF_SPLICE |
| 27 | RN7SK--RMRP | 5 | 24 | INCL_NON_REF_SPLICE |
| 27 | RN7SK--RMRP | 5 | 24 | INCL_NON_REF_SPLICE |
| 27 | RN7SK--RMRP | 4 | 24 | INCL_NON_REF_SPLICE |
| 27 | RN7SK--RMRP | 4 | 24 | INCL_NON_REF_SPLICE |
| 27 | RN7SK--RMRP | 4 | 24 | INCL_NON_REF_SPLICE |
| 27 | RN7SK--RMRP | 4 | 24 | INCL_NON_REF_SPLICE |
| 27 | RN7SK--RMRP | 4 | 24 | INCL_NON_REF_SPLICE |
| 27 | RN7SK--RMRP | 4 | 24 | INCL_NON_REF_SPLICE |
| 27 | RN7SK--RMRP | 4 | 24 | INCL_NON_REF_SPLICE |
| 27 | RN7SK--RMRP | 4 | 24 | INCL_NON_REF_SPLICE |
| 27 | RN7SK--RMRP | 4 | 24 | INCL_NON_REF_SPLICE |
| 27 | RN7SK--RMRP | 4 | 24 | INCL_NON_REF_SPLICE |
| 27 | RN7SK--RMRP | 4 | 24 | INCL_NON_REF_SPLICE |
| 27 | RN7SK--RMRP | 3 | 24 | INCL_NON_REF_SPLICE |
| 27 | RN7SK--RMRP | 3 | 24 | INCL_NON_REF_SPLICE |
| 27 | RN7SK--RMRP | 3 | 24 | INCL_NON_REF_SPLICE |
| 27 | RN7SK--RMRP | 3 | 24 | INCL_NON_REF_SPLICE |
| 27 | RN7SK--RMRP | 3 | 24 | INCL_NON_REF_SPLICE |
| 27 | RN7SK--RMRP | 3 | 24 | INCL_NON_REF_SPLICE |
| 27 | RN7SK--RMRP | 3 | 24 | INCL_NON_REF_SPLICE |
| 27 | RN7SK--RMRP | 3 | 24 | INCL_NON_REF_SPLICE |
| 27 | RN7SK--RMRP | 3 | 24 | INCL_NON_REF_SPLICE |
| 45 | RN7SK--RN7SKP267 | 8 | 0 | INCL_NON_REF_SPLICE |
| 34 | RN7SK--RN7SL1 | 15 | 102 | INCL_NON_REF_SPLICE |
| 34 | RN7SK--RN7SL1 | 11 | 102 | INCL_NON_REF_SPLICE |
| 34 | RN7SK--RN7SL1 | 11 | 102 | INCL_NON_REF_SPLICE |
| 34 | RN7SK--RN7SL1 | 9 | 102 | INCL_NON_REF_SPLICE |
| 34 | RN7SK--RN7SL1 | 9 | 102 | INCL_NON_REF_SPLICE |
| 34 | RN7SK--RN7SL1 | 8 | 102 | INCL_NON_REF_SPLICE |
| 34 | RN7SK--RN7SL1 | 8 | 102 | INCL_NON_REF_SPLICE |
| 34 | RN7SK--RN7SL1 | 7 | 102 | INCL_NON_REF_SPLICE |
| 34 | RN7SK--RN7SL1 | 6 | 102 | INCL_NON_REF_SPLICE |
| 34 | RN7SK--RN7SL1 | 6 | 102 | INCL_NON_REF_SPLICE |
| 34 | RN7SK--RN7SL1 | 5 | 102 | INCL_NON_REF_SPLICE |
| 34 | RN7SK--RN7SL1 | 5 | 102 | INCL_NON_REF_SPLICE |
| 34 | RN7SK--RN7SL1 | 4 | 102 | INCL_NON_REF_SPLICE |
| 34 | RN7SK--RN7SL1 | 4 | 102 | INCL_NON_REF_SPLICE |
| 34 | RN7SK--RN7SL1 | 4 | 102 | INCL_NON_REF_SPLICE |
| 34 | RN7SK--RN7SL1 | 3 | 102 | INCL_NON_REF_SPLICE |
| 34 | RN7SK--RN7SL1 | 3 | 102 | INCL_NON_REF_SPLICE |
| 34 | RN7SK--RN7SL1 | 3 | 102 | INCL_NON_REF_SPLICE |
| 34 | RN7SK--RN7SL1 | 3 | 102 | INCL_NON_REF_SPLICE |
| 34 | RN7SK--RN7SL1 | 3 | 102 | INCL_NON_REF_SPLICE |
| 34 | RN7SK--RN7SL1 | 3 | 102 | INCL_NON_REF_SPLICE |
| 34 | RN7SK--RN7SL1 | 3 | 102 | INCL_NON_REF_SPLICE |
| 34 | RN7SK--RN7SL1 | 3 | 102 | INCL_NON_REF_SPLICE |
| 20 | RN7SK--RN7SL1 | 12 | 31 | INCL_NON_REF_SPLICE |
| 20 | RN7SK--RN7SL1 | 7 | 31 | INCL_NON_REF_SPLICE |
| 20 | RN7SK--RN7SL1 | 5 | 31 | INCL_NON_REF_SPLICE |
| 20 | RN7SK--RN7SL1 | 4 | 31 | INCL_NON_REF_SPLICE |
| 20 | RN7SK--RN7SL1 | 4 | 31 | INCL_NON_REF_SPLICE |
| 20 | RN7SK--RN7SL1 | 4 | 31 | INCL_NON_REF_SPLICE |
| 20 | RN7SK--RN7SL1 | 4 | 31 | INCL_NON_REF_SPLICE |
| 20 | RN7SK--RN7SL1 | 3 | 31 | INCL_NON_REF_SPLICE |
| 20 | RN7SK--RN7SL1 | 3 | 31 | INCL_NON_REF_SPLICE |
| 20 | RN7SK--RN7SL1 | 3 | 31 | INCL_NON_REF_SPLICE |
| 20 | RN7SK--RN7SL1 | 3 | 31 | INCL_NON_REF_SPLICE |
| 20 | RN7SK--RN7SL1 | 3 | 31 | INCL_NON_REF_SPLICE |
| 20 | RN7SK--RN7SL1 | 3 | 31 | INCL_NON_REF_SPLICE |
| 20 | RN7SK--RN7SL1 | 3 | 31 | INCL_NON_REF_SPLICE |
| 20 | RN7SK--RN7SL1 | 3 | 31 | INCL_NON_REF_SPLICE |
| 30 | RN7SK--RN7SL2 | 13 | 20 | INCL_NON_REF_SPLICE |
| 30 | RN7SK--RN7SL2 | 12 | 20 | INCL_NON_REF_SPLICE |
| 30 | RN7SK--RN7SL2 | 11 | 20 | INCL_NON_REF_SPLICE |
| 30 | RN7SK--RN7SL2 | 10 | 20 | INCL_NON_REF_SPLICE |
| 30 | RN7SK--RN7SL2 | 8 | 20 | INCL_NON_REF_SPLICE |
| 30 | RN7SK--RN7SL2 | 8 | 20 | INCL_NON_REF_SPLICE |
| 30 | RN7SK--RN7SL2 | 7 | 20 | INCL_NON_REF_SPLICE |
| 30 | RN7SK--RN7SL2 | 6 | 20 | INCL_NON_REF_SPLICE |
| 30 | RN7SK--RN7SL2 | 5 | 20 | INCL_NON_REF_SPLICE |
| 30 | RN7SK--RN7SL2 | 5 | 20 | INCL_NON_REF_SPLICE |
| 30 | RN7SK--RN7SL2 | 5 | 20 | INCL_NON_REF_SPLICE |
| 30 | RN7SK--RN7SL2 | 5 | 20 | INCL_NON_REF_SPLICE |
| 30 | RN7SK--RN7SL2 | 4 | 20 | INCL_NON_REF_SPLICE |
| 30 | RN7SK--RN7SL2 | 3 | 20 | INCL_NON_REF_SPLICE |
| 30 | RN7SK--RN7SL2 | 3 | 20 | INCL_NON_REF_SPLICE |
| 30 | RN7SK--RN7SL2 | 3 | 20 | INCL_NON_REF_SPLICE |
| 30 | RN7SK--RN7SL2 | 3 | 20 | INCL_NON_REF_SPLICE |
| 30 | RN7SK--RN7SL2 | 3 | 20 | INCL_NON_REF_SPLICE |
| 30 | RN7SK--RN7SL2 | 3 | 20 | INCL_NON_REF_SPLICE |
| 30 | RN7SK--RN7SL2 | 3 | 20 | INCL_NON_REF_SPLICE |
| 30 | RN7SK--RN7SL2 | 3 | 20 | INCL_NON_REF_SPLICE |
| 30 | RN7SK--RN7SL2 | 3 | 20 | INCL_NON_REF_SPLICE |
| 49 | RN7SK--RN7SL2 | 7 | 75 | INCL_NON_REF_SPLICE |
| 49 | RN7SK--RN7SL2 | 6 | 75 | INCL_NON_REF_SPLICE |
| 49 | RN7SK--RN7SL2 | 4 | 75 | INCL_NON_REF_SPLICE |
| 49 | RN7SK--RN7SL2 | 4 | 75 | INCL_NON_REF_SPLICE |
| 49 | RN7SK--RN7SL2 | 4 | 75 | INCL_NON_REF_SPLICE |
| 49 | RN7SK--RN7SL2 | 4 | 75 | INCL_NON_REF_SPLICE |
| 49 | RN7SK--RN7SL2 | 3 | 75 | INCL_NON_REF_SPLICE |
| 49 | RN7SK--RN7SL2 | 3 | 75 | INCL_NON_REF_SPLICE |
| 49 | RN7SK--RN7SL2 | 3 | 75 | INCL_NON_REF_SPLICE |
| 49 | RN7SK--RN7SL2 | 3 | 75 | INCL_NON_REF_SPLICE |
| 49 | RN7SK--RN7SL2 | 3 | 75 | INCL_NON_REF_SPLICE |
| 27 | RN7SK--RN7SL2 | 23 | 44 | INCL_NON_REF_SPLICE |
| 27 | RN7SK--RN7SL2 | 17 | 44 | INCL_NON_REF_SPLICE |
| 27 | RN7SK--RN7SL2 | 15 | 44 | INCL_NON_REF_SPLICE |
| 27 | RN7SK--RN7SL2 | 15 | 44 | INCL_NON_REF_SPLICE |
| 27 | RN7SK--RN7SL2 | 13 | 44 | INCL_NON_REF_SPLICE |
| 27 | RN7SK--RN7SL2 | 13 | 44 | INCL_NON_REF_SPLICE |
| 27 | RN7SK--RN7SL2 | 11 | 44 | INCL_NON_REF_SPLICE |
| 27 | RN7SK--RN7SL2 | 10 | 44 | INCL_NON_REF_SPLICE |
| 27 | RN7SK--RN7SL2 | 9 | 44 | INCL_NON_REF_SPLICE |
| 27 | RN7SK--RN7SL2 | 9 | 44 | INCL_NON_REF_SPLICE |
| 27 | RN7SK--RN7SL2 | 8 | 44 | INCL_NON_REF_SPLICE |
| 27 | RN7SK--RN7SL2 | 8 | 44 | INCL_NON_REF_SPLICE |
| 27 | RN7SK--RN7SL2 | 8 | 44 | INCL_NON_REF_SPLICE |
| 27 | RN7SK--RN7SL2 | 8 | 44 | INCL_NON_REF_SPLICE |
| 27 | RN7SK--RN7SL2 | 8 | 44 | INCL_NON_REF_SPLICE |
| 27 | RN7SK--RN7SL2 | 7 | 44 | INCL_NON_REF_SPLICE |
| 27 | RN7SK--RN7SL2 | 7 | 44 | INCL_NON_REF_SPLICE |
| 27 | RN7SK--RN7SL2 | 7 | 44 | INCL_NON_REF_SPLICE |
| 27 | RN7SK--RN7SL2 | 6 | 44 | INCL_NON_REF_SPLICE |
| 27 | RN7SK--RN7SL2 | 6 | 44 | INCL_NON_REF_SPLICE |
| 27 | RN7SK--RN7SL2 | 6 | 44 | INCL_NON_REF_SPLICE |
| 27 | RN7SK--RN7SL2 | 6 | 44 | INCL_NON_REF_SPLICE |
| 27 | RN7SK--RN7SL2 | 6 | 44 | INCL_NON_REF_SPLICE |
| 27 | RN7SK--RN7SL2 | 5 | 44 | INCL_NON_REF_SPLICE |
| 27 | RN7SK--RN7SL2 | 5 | 44 | INCL_NON_REF_SPLICE |
| 27 | RN7SK--RN7SL2 | 5 | 44 | INCL_NON_REF_SPLICE |
| 27 | RN7SK--RN7SL2 | 5 | 44 | INCL_NON_REF_SPLICE |
| 27 | RN7SK--RN7SL2 | 5 | 44 | INCL_NON_REF_SPLICE |
| 27 | RN7SK--RN7SL2 | 5 | 44 | INCL_NON_REF_SPLICE |
| 27 | RN7SK--RN7SL2 | 5 | 44 | INCL_NON_REF_SPLICE |
| 27 | RN7SK--RN7SL2 | 4 | 44 | INCL_NON_REF_SPLICE |
| 27 | RN7SK--RN7SL2 | 4 | 44 | INCL_NON_REF_SPLICE |
| 27 | RN7SK--RN7SL2 | 4 | 44 | INCL_NON_REF_SPLICE |
| 27 | RN7SK--RN7SL2 | 4 | 44 | INCL_NON_REF_SPLICE |
| 27 | RN7SK--RN7SL2 | 4 | 44 | INCL_NON_REF_SPLICE |
| 27 | RN7SK--RN7SL2 | 4 | 44 | INCL_NON_REF_SPLICE |
| 27 | RN7SK--RN7SL2 | 4 | 44 | INCL_NON_REF_SPLICE |
| 27 | RN7SK--RN7SL2 | 4 | 44 | INCL_NON_REF_SPLICE |
| 27 | RN7SK--RN7SL2 | 4 | 44 | INCL_NON_REF_SPLICE |
| 27 | RN7SK--RN7SL2 | 4 | 44 | INCL_NON_REF_SPLICE |
| 27 | RN7SK--RN7SL2 | 4 | 44 | INCL_NON_REF_SPLICE |
| 27 | RN7SK--RN7SL2 | 4 | 44 | INCL_NON_REF_SPLICE |
| 27 | RN7SK--RN7SL2 | 3 | 44 | INCL_NON_REF_SPLICE |
| 27 | RN7SK--RN7SL2 | 3 | 44 | INCL_NON_REF_SPLICE |
| 27 | RN7SK--RN7SL2 | 3 | 44 | INCL_NON_REF_SPLICE |
| 27 | RN7SK--RN7SL2 | 3 | 44 | INCL_NON_REF_SPLICE |
| 27 | RN7SK--RN7SL2 | 3 | 44 | INCL_NON_REF_SPLICE |
| 27 | RN7SK--RN7SL2 | 3 | 44 | INCL_NON_REF_SPLICE |
| 27 | RN7SK--RN7SL2 | 3 | 44 | INCL_NON_REF_SPLICE |
| 27 | RN7SK--RN7SL2 | 3 | 44 | INCL_NON_REF_SPLICE |
| 27 | RN7SK--RN7SL2 | 3 | 44 | INCL_NON_REF_SPLICE |
| 27 | RN7SK--RN7SL2 | 3 | 44 | INCL_NON_REF_SPLICE |
| 27 | RN7SK--RN7SL2 | 3 | 44 | INCL_NON_REF_SPLICE |
| 27 | RN7SK--RN7SL2 | 3 | 44 | INCL_NON_REF_SPLICE |
| 27 | RN7SK--RN7SL2 | 3 | 44 | INCL_NON_REF_SPLICE |
| 27 | RN7SK--RN7SL2 | 3 | 44 | INCL_NON_REF_SPLICE |
| 27 | RN7SK--RN7SL2 | 3 | 44 | INCL_NON_REF_SPLICE |
| 27 | RN7SK--RN7SL2 | 3 | 44 | INCL_NON_REF_SPLICE |
| 27 | RN7SK--RN7SL2 | 3 | 44 | INCL_NON_REF_SPLICE |
| 27 | RN7SK--RN7SL2 | 3 | 44 | INCL_NON_REF_SPLICE |
| 27 | RN7SK--RN7SL2 | 3 | 44 | INCL_NON_REF_SPLICE |
| 27 | RN7SK--RN7SL2 | 3 | 44 | INCL_NON_REF_SPLICE |
| 27 | RN7SK--RN7SL2 | 3 | 44 | INCL_NON_REF_SPLICE |
| 27 | RN7SK--RN7SL2 | 3 | 44 | INCL_NON_REF_SPLICE |
| 27 | RN7SK--RN7SL2 | 3 | 44 | INCL_NON_REF_SPLICE |
| 27 | RN7SK--RN7SL2 | 3 | 44 | INCL_NON_REF_SPLICE |
| 27 | RN7SK--RN7SL2 | 3 | 44 | INCL_NON_REF_SPLICE |
| 27 | RN7SK--RN7SL2 | 3 | 44 | INCL_NON_REF_SPLICE |
| 27 | RN7SK--RN7SL2 | 3 | 44 | INCL_NON_REF_SPLICE |
| 49 | RN7SK--RNA5-8SN2 | 4 | 1 | INCL_NON_REF_SPLICE |
| 49 | RN7SK--RNA5-8SN2 | 4 | 1 | INCL_NON_REF_SPLICE |
| 49 | RN7SK--RNA5-8SN2 | 4 | 1 | INCL_NON_REF_SPLICE |
| 49 | RN7SK--RNA5-8SN2 | 4 | 1 | INCL_NON_REF_SPLICE |
| 27 | RN7SK--RNU1-27P | 47 | 0 | INCL_NON_REF_SPLICE |
| 27 | RN7SK--RNU1-27P | 31 | 0 | INCL_NON_REF_SPLICE |
| 34 | RN7SK--RPPH1 | 6 | 64 | INCL_NON_REF_SPLICE |
| 34 | RN7SK--RPPH1 | 6 | 64 | INCL_NON_REF_SPLICE |
| 34 | RN7SK--RPPH1 | 3 | 64 | INCL_NON_REF_SPLICE |
| 34 | RN7SK--RPPH1 | 3 | 64 | INCL_NON_REF_SPLICE |
| 27 | RN7SK--RPPH1 | 13 | 52 | INCL_NON_REF_SPLICE |
| 27 | RN7SK--RPPH1 | 8 | 52 | INCL_NON_REF_SPLICE |
| 27 | RN7SK--RPPH1 | 7 | 52 | INCL_NON_REF_SPLICE |
| 27 | RN7SK--RPPH1 | 6 | 52 | INCL_NON_REF_SPLICE |
| 27 | RN7SK--RPPH1 | 6 | 52 | INCL_NON_REF_SPLICE |
| 27 | RN7SK--RPPH1 | 5 | 52 | INCL_NON_REF_SPLICE |
| 27 | RN7SK--RPPH1 | 5 | 52 | INCL_NON_REF_SPLICE |
| 27 | RN7SK--RPPH1 | 5 | 52 | INCL_NON_REF_SPLICE |
| 27 | RN7SK--RPPH1 | 4 | 52 | INCL_NON_REF_SPLICE |
| 27 | RN7SK--RPPH1 | 4 | 52 | INCL_NON_REF_SPLICE |
| 27 | RN7SK--RPPH1 | 4 | 52 | INCL_NON_REF_SPLICE |
| 27 | RN7SK--RPPH1 | 4 | 52 | INCL_NON_REF_SPLICE |
| 27 | RN7SK--RPPH1 | 4 | 52 | INCL_NON_REF_SPLICE |
| 27 | RN7SK--RPPH1 | 4 | 52 | INCL_NON_REF_SPLICE |
| 27 | RN7SK--RPPH1 | 4 | 52 | INCL_NON_REF_SPLICE |
| 27 | RN7SK--RPPH1 | 3 | 52 | INCL_NON_REF_SPLICE |
| 27 | RN7SK--RPPH1 | 3 | 52 | INCL_NON_REF_SPLICE |
| 27 | RN7SK--RPPH1 | 3 | 52 | INCL_NON_REF_SPLICE |
| 27 | RN7SK--RPPH1 | 3 | 52 | INCL_NON_REF_SPLICE |
| 27 | RN7SK--RPPH1 | 3 | 52 | INCL_NON_REF_SPLICE |
| 27 | RN7SK--RPPH1 | 3 | 52 | INCL_NON_REF_SPLICE |
| 27 | RN7SK--RPPH1 | 3 | 52 | INCL_NON_REF_SPLICE |
| 27 | RN7SK--RPPH1 | 3 | 52 | INCL_NON_REF_SPLICE |
| 27 | RN7SK--RPPH1 | 3 | 52 | INCL_NON_REF_SPLICE |
| 27 | RN7SK--RPPH1 | 3 | 52 | INCL_NON_REF_SPLICE |
| 27 | RN7SK--RPPH1 | 3 | 52 | INCL_NON_REF_SPLICE |
| 27 | RN7SK--RPPH1 | 3 | 52 | INCL_NON_REF_SPLICE |
| 34 | RN7SK--SNORA73B | 30 | 38 | INCL_NON_REF_SPLICE |
| 34 | RN7SK--SNORA73B | 26 | 38 | INCL_NON_REF_SPLICE |
| 34 | RN7SK--SNORA73B | 17 | 38 | INCL_NON_REF_SPLICE |
| 34 | RN7SK--SNORA73B | 11 | 38 | INCL_NON_REF_SPLICE |
| 34 | RN7SK--SNORA73B | 7 | 38 | INCL_NON_REF_SPLICE |
| 34 | RN7SK--SNORA73B | 6 | 38 | INCL_NON_REF_SPLICE |
| 34 | RN7SK--SNORA73B | 6 | 38 | INCL_NON_REF_SPLICE |
| 34 | RN7SK--SNORA73B | 5 | 38 | INCL_NON_REF_SPLICE |
| 34 | RN7SK--SNORA73B | 4 | 38 | INCL_NON_REF_SPLICE |
| 34 | RN7SK--SNORA73B | 4 | 38 | INCL_NON_REF_SPLICE |
| 34 | RN7SK--SNORA73B | 4 | 38 | INCL_NON_REF_SPLICE |
| 34 | RN7SK--SNORA73B | 3 | 38 | INCL_NON_REF_SPLICE |
| 27 | RN7SK--SNORD3A | 25 | 1 | INCL_NON_REF_SPLICE |
| 20 | RN7SL116P--AL139099.4 | 15 | 0 | INCL_NON_REF_SPLICE |
| 41 | RN7SL1--IFI6 | 64 | 10 | INCL_NON_REF_SPLICE |
| 41 | RN7SL1--IFI6 | 13 | 10 | INCL_NON_REF_SPLICE |
| 20 | RN7SL1--PLCG2 | 7 | 3 | INCL_NON_REF_SPLICE |
| 20 | RN7SL1--PLCG2 | 6 | 3 | INCL_NON_REF_SPLICE |
| 49 | RN7SL1--RF00100 | 3 | 101 | INCL_NON_REF_SPLICE |
| 49 | RN7SL1--RF00100 | 3 | 101 | INCL_NON_REF_SPLICE |
| 49 | RN7SL1--RF00100 | 3 | 101 | INCL_NON_REF_SPLICE |
| 49 | RN7SL1--RF00100 | 3 | 101 | INCL_NON_REF_SPLICE |
| 34 | RN7SL1--RF00100 | 11 | 165 | INCL_NON_REF_SPLICE |
| 34 | RN7SL1--RF00100 | 8 | 165 | INCL_NON_REF_SPLICE |
| 34 | RN7SL1--RF00100 | 7 | 165 | INCL_NON_REF_SPLICE |
| 34 | RN7SL1--RF00100 | 7 | 165 | INCL_NON_REF_SPLICE |
| 34 | RN7SL1--RF00100 | 7 | 165 | INCL_NON_REF_SPLICE |
| 34 | RN7SL1--RF00100 | 6 | 165 | INCL_NON_REF_SPLICE |
| 34 | RN7SL1--RF00100 | 6 | 165 | INCL_NON_REF_SPLICE |
| 34 | RN7SL1--RF00100 | 6 | 165 | INCL_NON_REF_SPLICE |
| 34 | RN7SL1--RF00100 | 5 | 165 | INCL_NON_REF_SPLICE |
| 34 | RN7SL1--RF00100 | 5 | 165 | INCL_NON_REF_SPLICE |
| 34 | RN7SL1--RF00100 | 5 | 165 | INCL_NON_REF_SPLICE |
| 34 | RN7SL1--RF00100 | 5 | 165 | INCL_NON_REF_SPLICE |
| 34 | RN7SL1--RF00100 | 5 | 165 | INCL_NON_REF_SPLICE |
| 34 | RN7SL1--RF00100 | 5 | 165 | INCL_NON_REF_SPLICE |
| 34 | RN7SL1--RF00100 | 5 | 165 | INCL_NON_REF_SPLICE |
| 34 | RN7SL1--RF00100 | 5 | 165 | INCL_NON_REF_SPLICE |
| 34 | RN7SL1--RF00100 | 4 | 165 | INCL_NON_REF_SPLICE |
| 34 | RN7SL1--RF00100 | 4 | 165 | INCL_NON_REF_SPLICE |
| 34 | RN7SL1--RF00100 | 4 | 165 | INCL_NON_REF_SPLICE |
| 34 | RN7SL1--RF00100 | 4 | 165 | INCL_NON_REF_SPLICE |
| 34 | RN7SL1--RF00100 | 4 | 165 | INCL_NON_REF_SPLICE |
| 34 | RN7SL1--RF00100 | 4 | 165 | INCL_NON_REF_SPLICE |
| 34 | RN7SL1--RF00100 | 4 | 165 | INCL_NON_REF_SPLICE |
| 34 | RN7SL1--RF00100 | 3 | 165 | INCL_NON_REF_SPLICE |
| 34 | RN7SL1--RF00100 | 3 | 165 | INCL_NON_REF_SPLICE |
| 34 | RN7SL1--RF00100 | 3 | 165 | INCL_NON_REF_SPLICE |
| 34 | RN7SL1--RF00100 | 3 | 165 | INCL_NON_REF_SPLICE |
| 34 | RN7SL1--RF00100 | 3 | 165 | INCL_NON_REF_SPLICE |
| 34 | RN7SL1--RF00100 | 3 | 165 | INCL_NON_REF_SPLICE |
| 34 | RN7SL1--RF00100 | 3 | 165 | INCL_NON_REF_SPLICE |
| 34 | RN7SL1--RF00100 | 3 | 165 | INCL_NON_REF_SPLICE |
| 34 | RN7SL1--RF00100 | 3 | 165 | INCL_NON_REF_SPLICE |
| 34 | RN7SL1--RF00100 | 3 | 165 | INCL_NON_REF_SPLICE |
| 34 | RN7SL1--RF00100 | 3 | 165 | INCL_NON_REF_SPLICE |
| 34 | RN7SL1--RF00100 | 3 | 165 | INCL_NON_REF_SPLICE |
| 20 | RN7SL1--RF00100 | 8 | 36 | INCL_NON_REF_SPLICE |
| 20 | RN7SL1--RF00100 | 7 | 36 | INCL_NON_REF_SPLICE |
| 20 | RN7SL1--RF00100 | 6 | 36 | INCL_NON_REF_SPLICE |
| 20 | RN7SL1--RF00100 | 5 | 36 | INCL_NON_REF_SPLICE |
| 20 | RN7SL1--RF00100 | 5 | 36 | INCL_NON_REF_SPLICE |
| 20 | RN7SL1--RF00100 | 5 | 36 | INCL_NON_REF_SPLICE |
| 20 | RN7SL1--RF00100 | 4 | 36 | INCL_NON_REF_SPLICE |
| 20 | RN7SL1--RF00100 | 4 | 36 | INCL_NON_REF_SPLICE |
| 20 | RN7SL1--RF00100 | 4 | 36 | INCL_NON_REF_SPLICE |
| 20 | RN7SL1--RF00100 | 4 | 36 | INCL_NON_REF_SPLICE |
| 20 | RN7SL1--RF00100 | 4 | 36 | INCL_NON_REF_SPLICE |
| 20 | RN7SL1--RF00100 | 4 | 36 | INCL_NON_REF_SPLICE |
| 20 | RN7SL1--RF00100 | 4 | 36 | INCL_NON_REF_SPLICE |
| 20 | RN7SL1--RF00100 | 4 | 36 | INCL_NON_REF_SPLICE |
| 20 | RN7SL1--RF00100 | 3 | 36 | INCL_NON_REF_SPLICE |
| 20 | RN7SL1--RF00100 | 3 | 36 | INCL_NON_REF_SPLICE |
| 20 | RN7SL1--RF00100 | 3 | 36 | INCL_NON_REF_SPLICE |
| 20 | RN7SL1--RF00100 | 3 | 36 | INCL_NON_REF_SPLICE |
| 20 | RN7SL1--RF00100 | 3 | 36 | INCL_NON_REF_SPLICE |
| 20 | RN7SL1--RF00100 | 3 | 36 | INCL_NON_REF_SPLICE |
| 20 | RN7SL1--RF00100 | 3 | 36 | INCL_NON_REF_SPLICE |
| 20 | RN7SL1--RF00100 | 3 | 36 | INCL_NON_REF_SPLICE |
| 30 | RN7SL1--RMRP | 5 | 16 | INCL_NON_REF_SPLICE |
| 30 | RN7SL1--RMRP | 4 | 16 | INCL_NON_REF_SPLICE |
| 30 | RN7SL1--RMRP | 3 | 16 | INCL_NON_REF_SPLICE |
| 30 | RN7SL1--RMRP | 3 | 16 | INCL_NON_REF_SPLICE |
| 30 | RN7SL1--RMRP | 3 | 16 | INCL_NON_REF_SPLICE |
| 30 | RN7SL1--RMRP | 3 | 16 | INCL_NON_REF_SPLICE |
| 30 | RN7SL1--RMRP | 3 | 16 | INCL_NON_REF_SPLICE |
| 20 | RN7SL1--RMRP | 11 | 26 | INCL_NON_REF_SPLICE |
| 20 | RN7SL1--RMRP | 8 | 26 | INCL_NON_REF_SPLICE |
| 20 | RN7SL1--RMRP | 5 | 26 | INCL_NON_REF_SPLICE |
| 20 | RN7SL1--RMRP | 4 | 26 | INCL_NON_REF_SPLICE |
| 20 | RN7SL1--RMRP | 4 | 26 | INCL_NON_REF_SPLICE |
| 20 | RN7SL1--RMRP | 3 | 26 | INCL_NON_REF_SPLICE |
| 20 | RN7SL1--RMRP | 3 | 26 | INCL_NON_REF_SPLICE |
| 20 | RN7SL1--RMRP | 3 | 26 | INCL_NON_REF_SPLICE |
| 20 | RN7SL1--RMRP | 3 | 26 | INCL_NON_REF_SPLICE |
| 20 | RN7SL1--RMRP | 3 | 26 | INCL_NON_REF_SPLICE |
| 20 | RN7SL1--RMRP | 3 | 26 | INCL_NON_REF_SPLICE |
| 20 | RN7SL1--RMRP | 3 | 26 | INCL_NON_REF_SPLICE |
| 49 | RN7SL1--RN7SK | 3 | 101 | INCL_NON_REF_SPLICE |
| 49 | RN7SL1--RN7SK | 3 | 101 | INCL_NON_REF_SPLICE |
| 49 | RN7SL1--RN7SK | 3 | 101 | INCL_NON_REF_SPLICE |
| 49 | RN7SL1--RN7SK | 3 | 101 | INCL_NON_REF_SPLICE |
| 34 | RN7SL1--RN7SK | 11 | 165 | INCL_NON_REF_SPLICE |
| 34 | RN7SL1--RN7SK | 8 | 165 | INCL_NON_REF_SPLICE |
| 34 | RN7SL1--RN7SK | 7 | 165 | INCL_NON_REF_SPLICE |
| 34 | RN7SL1--RN7SK | 7 | 165 | INCL_NON_REF_SPLICE |
| 34 | RN7SL1--RN7SK | 7 | 165 | INCL_NON_REF_SPLICE |
| 34 | RN7SL1--RN7SK | 6 | 165 | INCL_NON_REF_SPLICE |
| 34 | RN7SL1--RN7SK | 6 | 165 | INCL_NON_REF_SPLICE |
| 34 | RN7SL1--RN7SK | 6 | 165 | INCL_NON_REF_SPLICE |
| 34 | RN7SL1--RN7SK | 5 | 165 | INCL_NON_REF_SPLICE |
| 34 | RN7SL1--RN7SK | 5 | 165 | INCL_NON_REF_SPLICE |
| 34 | RN7SL1--RN7SK | 5 | 165 | INCL_NON_REF_SPLICE |
| 34 | RN7SL1--RN7SK | 5 | 165 | INCL_NON_REF_SPLICE |
| 34 | RN7SL1--RN7SK | 5 | 165 | INCL_NON_REF_SPLICE |
| 34 | RN7SL1--RN7SK | 5 | 165 | INCL_NON_REF_SPLICE |
| 34 | RN7SL1--RN7SK | 5 | 165 | INCL_NON_REF_SPLICE |
| 34 | RN7SL1--RN7SK | 5 | 165 | INCL_NON_REF_SPLICE |
| 34 | RN7SL1--RN7SK | 4 | 165 | INCL_NON_REF_SPLICE |
| 34 | RN7SL1--RN7SK | 4 | 165 | INCL_NON_REF_SPLICE |
| 34 | RN7SL1--RN7SK | 4 | 165 | INCL_NON_REF_SPLICE |
| 34 | RN7SL1--RN7SK | 4 | 165 | INCL_NON_REF_SPLICE |
| 34 | RN7SL1--RN7SK | 4 | 165 | INCL_NON_REF_SPLICE |
| 34 | RN7SL1--RN7SK | 4 | 165 | INCL_NON_REF_SPLICE |
| 34 | RN7SL1--RN7SK | 4 | 165 | INCL_NON_REF_SPLICE |
| 34 | RN7SL1--RN7SK | 3 | 165 | INCL_NON_REF_SPLICE |
| 34 | RN7SL1--RN7SK | 3 | 165 | INCL_NON_REF_SPLICE |
| 34 | RN7SL1--RN7SK | 3 | 165 | INCL_NON_REF_SPLICE |
| 34 | RN7SL1--RN7SK | 3 | 165 | INCL_NON_REF_SPLICE |
| 34 | RN7SL1--RN7SK | 3 | 165 | INCL_NON_REF_SPLICE |
| 34 | RN7SL1--RN7SK | 3 | 165 | INCL_NON_REF_SPLICE |
| 34 | RN7SL1--RN7SK | 3 | 165 | INCL_NON_REF_SPLICE |
| 34 | RN7SL1--RN7SK | 3 | 165 | INCL_NON_REF_SPLICE |
| 34 | RN7SL1--RN7SK | 3 | 165 | INCL_NON_REF_SPLICE |
| 34 | RN7SL1--RN7SK | 3 | 165 | INCL_NON_REF_SPLICE |
| 34 | RN7SL1--RN7SK | 3 | 165 | INCL_NON_REF_SPLICE |
| 34 | RN7SL1--RN7SK | 3 | 165 | INCL_NON_REF_SPLICE |
| 20 | RN7SL1--RN7SK | 8 | 36 | INCL_NON_REF_SPLICE |
| 20 | RN7SL1--RN7SK | 7 | 36 | INCL_NON_REF_SPLICE |
| 20 | RN7SL1--RN7SK | 6 | 36 | INCL_NON_REF_SPLICE |
| 20 | RN7SL1--RN7SK | 5 | 36 | INCL_NON_REF_SPLICE |
| 20 | RN7SL1--RN7SK | 5 | 36 | INCL_NON_REF_SPLICE |
| 20 | RN7SL1--RN7SK | 5 | 36 | INCL_NON_REF_SPLICE |
| 20 | RN7SL1--RN7SK | 4 | 36 | INCL_NON_REF_SPLICE |
| 20 | RN7SL1--RN7SK | 4 | 36 | INCL_NON_REF_SPLICE |
| 20 | RN7SL1--RN7SK | 4 | 36 | INCL_NON_REF_SPLICE |
| 20 | RN7SL1--RN7SK | 4 | 36 | INCL_NON_REF_SPLICE |
| 20 | RN7SL1--RN7SK | 4 | 36 | INCL_NON_REF_SPLICE |
| 20 | RN7SL1--RN7SK | 4 | 36 | INCL_NON_REF_SPLICE |
| 20 | RN7SL1--RN7SK | 4 | 36 | INCL_NON_REF_SPLICE |
| 20 | RN7SL1--RN7SK | 4 | 36 | INCL_NON_REF_SPLICE |
| 20 | RN7SL1--RN7SK | 3 | 36 | INCL_NON_REF_SPLICE |
| 20 | RN7SL1--RN7SK | 3 | 36 | INCL_NON_REF_SPLICE |
| 20 | RN7SL1--RN7SK | 3 | 36 | INCL_NON_REF_SPLICE |
| 20 | RN7SL1--RN7SK | 3 | 36 | INCL_NON_REF_SPLICE |
| 20 | RN7SL1--RN7SK | 3 | 36 | INCL_NON_REF_SPLICE |
| 20 | RN7SL1--RN7SK | 3 | 36 | INCL_NON_REF_SPLICE |
| 20 | RN7SL1--RN7SK | 3 | 36 | INCL_NON_REF_SPLICE |
| 20 | RN7SL1--RN7SK | 3 | 36 | INCL_NON_REF_SPLICE |
| 30 | RN7SL1--RNU5B-1 | 9 | 0 | INCL_NON_REF_SPLICE |
| 34 | RN7SL1--SNORA73B | 33 | 39 | INCL_NON_REF_SPLICE |
| 34 | RN7SL1--SNORA73B | 19 | 39 | INCL_NON_REF_SPLICE |
| 34 | RN7SL1--SNORA73B | 17 | 39 | INCL_NON_REF_SPLICE |
| 34 | RN7SL1--SNORA73B | 12 | 39 | INCL_NON_REF_SPLICE |
| 34 | RN7SL1--SNORA73B | 10 | 39 | INCL_NON_REF_SPLICE |
| 34 | RN7SL1--SNORA73B | 10 | 39 | INCL_NON_REF_SPLICE |
| 34 | RN7SL1--SNORA73B | 10 | 39 | INCL_NON_REF_SPLICE |
| 34 | RN7SL1--SNORA73B | 9 | 39 | INCL_NON_REF_SPLICE |
| 34 | RN7SL1--SNORA73B | 9 | 39 | INCL_NON_REF_SPLICE |
| 34 | RN7SL1--SNORA73B | 7 | 39 | INCL_NON_REF_SPLICE |
| 30 | RN7SL2--AL355075.4 | 13 | 18 | INCL_NON_REF_SPLICE |
| 30 | RN7SL2--AL355075.4 | 4 | 18 | INCL_NON_REF_SPLICE |
| 30 | RN7SL2--AL355075.4 | 4 | 18 | INCL_NON_REF_SPLICE |
| 30 | RN7SL2--AL355075.4 | 3 | 18 | INCL_NON_REF_SPLICE |
| 30 | RN7SL2--AL355075.4 | 3 | 18 | INCL_NON_REF_SPLICE |
| 30 | RN7SL2--AL355075.4 | 3 | 18 | INCL_NON_REF_SPLICE |
| 30 | RN7SL2--AL355075.4 | 3 | 18 | INCL_NON_REF_SPLICE |
| 30 | RN7SL2--AL355075.4 | 3 | 18 | INCL_NON_REF_SPLICE |
| 30 | RN7SL2--AL355075.4 | 3 | 18 | INCL_NON_REF_SPLICE |
| 49 | RN7SL2--AL355075.4 | 4 | 84 | INCL_NON_REF_SPLICE |
| 34 | RN7SL2--AL355075.4 | 16 | 90 | INCL_NON_REF_SPLICE |
| 34 | RN7SL2--AL355075.4 | 8 | 90 | INCL_NON_REF_SPLICE |
| 34 | RN7SL2--AL355075.4 | 4 | 90 | INCL_NON_REF_SPLICE |
| 34 | RN7SL2--AL355075.4 | 4 | 90 | INCL_NON_REF_SPLICE |
| 34 | RN7SL2--AL355075.4 | 3 | 90 | INCL_NON_REF_SPLICE |
| 34 | RN7SL2--AL355075.4 | 3 | 90 | INCL_NON_REF_SPLICE |
| 34 | RN7SL2--AL355075.4 | 3 | 90 | INCL_NON_REF_SPLICE |
| 34 | RN7SL2--AL355075.4 | 3 | 90 | INCL_NON_REF_SPLICE |
| 34 | RN7SL2--AL355075.4 | 3 | 90 | INCL_NON_REF_SPLICE |
| 34 | RN7SL2--AL355075.4 | 3 | 90 | INCL_NON_REF_SPLICE |
| 34 | RN7SL2--AL355075.4 | 3 | 90 | INCL_NON_REF_SPLICE |
| 34 | RN7SL2--AL355075.4 | 3 | 90 | INCL_NON_REF_SPLICE |
| 27 | RN7SL2--AL355075.4 | 54 | 82 | INCL_NON_REF_SPLICE |
| 27 | RN7SL2--AL355075.4 | 20 | 82 | INCL_NON_REF_SPLICE |
| 27 | RN7SL2--AL355075.4 | 17 | 82 | INCL_NON_REF_SPLICE |
| 27 | RN7SL2--AL355075.4 | 15 | 82 | INCL_NON_REF_SPLICE |
| 27 | RN7SL2--AL355075.4 | 14 | 82 | INCL_NON_REF_SPLICE |
| 27 | RN7SL2--AL355075.4 | 13 | 82 | INCL_NON_REF_SPLICE |
| 27 | RN7SL2--AL355075.4 | 10 | 82 | INCL_NON_REF_SPLICE |
| 27 | RN7SL2--AL355075.4 | 10 | 82 | INCL_NON_REF_SPLICE |
| 27 | RN7SL2--AL355075.4 | 9 | 82 | INCL_NON_REF_SPLICE |
| 27 | RN7SL2--AL355075.4 | 9 | 82 | INCL_NON_REF_SPLICE |
| 27 | RN7SL2--AL355075.4 | 9 | 82 | INCL_NON_REF_SPLICE |
| 27 | RN7SL2--AL355075.4 | 9 | 82 | INCL_NON_REF_SPLICE |
| 27 | RN7SL2--AL355075.4 | 7 | 82 | INCL_NON_REF_SPLICE |
| 27 | RN7SL2--AL355075.4 | 7 | 82 | INCL_NON_REF_SPLICE |
| 27 | RN7SL2--AL355075.4 | 7 | 82 | INCL_NON_REF_SPLICE |
| 30 | RN7SL2--PLCG2 | 13 | 0 | INCL_NON_REF_SPLICE |
| 27 | RN7SL2--PLCG2 | 26 | 4 | INCL_NON_REF_SPLICE |
| 30 | RN7SL2--RF00100 | 29 | 35 | INCL_NON_REF_SPLICE |
| 30 | RN7SL2--RF00100 | 14 | 35 | INCL_NON_REF_SPLICE |
| 30 | RN7SL2--RF00100 | 10 | 35 | INCL_NON_REF_SPLICE |
| 30 | RN7SL2--RF00100 | 9 | 35 | INCL_NON_REF_SPLICE |
| 30 | RN7SL2--RF00100 | 7 | 35 | INCL_NON_REF_SPLICE |
| 30 | RN7SL2--RF00100 | 7 | 35 | INCL_NON_REF_SPLICE |
| 30 | RN7SL2--RF00100 | 6 | 35 | INCL_NON_REF_SPLICE |
| 30 | RN7SL2--RF00100 | 6 | 35 | INCL_NON_REF_SPLICE |
| 30 | RN7SL2--RF00100 | 6 | 35 | INCL_NON_REF_SPLICE |
| 30 | RN7SL2--RF00100 | 5 | 35 | INCL_NON_REF_SPLICE |
| 30 | RN7SL2--RF00100 | 5 | 35 | INCL_NON_REF_SPLICE |
| 30 | RN7SL2--RF00100 | 5 | 35 | INCL_NON_REF_SPLICE |
| 30 | RN7SL2--RF00100 | 5 | 35 | INCL_NON_REF_SPLICE |
| 30 | RN7SL2--RF00100 | 4 | 35 | INCL_NON_REF_SPLICE |
| 30 | RN7SL2--RF00100 | 4 | 35 | INCL_NON_REF_SPLICE |
| 30 | RN7SL2--RF00100 | 3 | 35 | INCL_NON_REF_SPLICE |
| 30 | RN7SL2--RF00100 | 3 | 35 | INCL_NON_REF_SPLICE |
| 30 | RN7SL2--RF00100 | 3 | 35 | INCL_NON_REF_SPLICE |
| 30 | RN7SL2--RF00100 | 3 | 35 | INCL_NON_REF_SPLICE |
| 30 | RN7SL2--RF00100 | 3 | 35 | INCL_NON_REF_SPLICE |
| 30 | RN7SL2--RF00100 | 3 | 35 | INCL_NON_REF_SPLICE |
| 30 | RN7SL2--RF00100 | 3 | 35 | INCL_NON_REF_SPLICE |
| 30 | RN7SL2--RF00100 | 3 | 35 | INCL_NON_REF_SPLICE |
| 30 | RN7SL2--RF00100 | 3 | 35 | INCL_NON_REF_SPLICE |
| 30 | RN7SL2--RF00100 | 3 | 35 | INCL_NON_REF_SPLICE |
| 30 | RN7SL2--RF00100 | 3 | 35 | INCL_NON_REF_SPLICE |
| 30 | RN7SL2--RF00100 | 3 | 35 | INCL_NON_REF_SPLICE |
| 30 | RN7SL2--RF00100 | 3 | 35 | INCL_NON_REF_SPLICE |
| 30 | RN7SL2--RF00100 | 3 | 35 | INCL_NON_REF_SPLICE |
| 27 | RN7SL2--RF00100 | 56 | 71 | INCL_NON_REF_SPLICE |
| 27 | RN7SL2--RF00100 | 28 | 71 | INCL_NON_REF_SPLICE |
| 27 | RN7SL2--RF00100 | 25 | 71 | INCL_NON_REF_SPLICE |
| 27 | RN7SL2--RF00100 | 22 | 71 | INCL_NON_REF_SPLICE |
| 27 | RN7SL2--RF00100 | 21 | 71 | INCL_NON_REF_SPLICE |
| 27 | RN7SL2--RF00100 | 18 | 71 | INCL_NON_REF_SPLICE |
| 27 | RN7SL2--RF00100 | 18 | 71 | INCL_NON_REF_SPLICE |
| 27 | RN7SL2--RF00100 | 14 | 71 | INCL_NON_REF_SPLICE |
| 27 | RN7SL2--RF00100 | 13 | 71 | INCL_NON_REF_SPLICE |
| 27 | RN7SL2--RF00100 | 11 | 71 | INCL_NON_REF_SPLICE |
| 27 | RN7SL2--RF00100 | 11 | 71 | INCL_NON_REF_SPLICE |
| 27 | RN7SL2--RF00100 | 11 | 71 | INCL_NON_REF_SPLICE |
| 27 | RN7SL2--RF00100 | 11 | 71 | INCL_NON_REF_SPLICE |
| 27 | RN7SL2--RF00100 | 11 | 71 | INCL_NON_REF_SPLICE |
| 27 | RN7SL2--RF00100 | 11 | 71 | INCL_NON_REF_SPLICE |
| 27 | RN7SL2--RF00100 | 9 | 71 | INCL_NON_REF_SPLICE |
| 27 | RN7SL2--RF00100 | 8 | 71 | INCL_NON_REF_SPLICE |
| 27 | RN7SL2--RF00100 | 8 | 71 | INCL_NON_REF_SPLICE |
| 27 | RN7SL2--RF00100 | 8 | 71 | INCL_NON_REF_SPLICE |
| 27 | RN7SL2--RF00100 | 8 | 71 | INCL_NON_REF_SPLICE |
| 27 | RN7SL2--RF00100 | 8 | 71 | INCL_NON_REF_SPLICE |
| 27 | RN7SL2--RF00100 | 7 | 71 | INCL_NON_REF_SPLICE |
| 27 | RN7SL2--RF00100 | 7 | 71 | INCL_NON_REF_SPLICE |
| 27 | RN7SL2--RF00100 | 7 | 71 | INCL_NON_REF_SPLICE |
| 27 | RN7SL2--RF00100 | 7 | 71 | INCL_NON_REF_SPLICE |
| 27 | RN7SL2--RF00100 | 7 | 71 | INCL_NON_REF_SPLICE |
| 27 | RN7SL2--RF00100 | 7 | 71 | INCL_NON_REF_SPLICE |
| 27 | RN7SL2--RF00100 | 7 | 71 | INCL_NON_REF_SPLICE |
| 27 | RN7SL2--RF00100 | 7 | 71 | INCL_NON_REF_SPLICE |
| 27 | RN7SL2--RF00100 | 7 | 71 | INCL_NON_REF_SPLICE |
| 27 | RN7SL2--RF00100 | 6 | 71 | INCL_NON_REF_SPLICE |
| 27 | RN7SL2--RF00100 | 6 | 71 | INCL_NON_REF_SPLICE |
| 27 | RN7SL2--RF00100 | 6 | 71 | INCL_NON_REF_SPLICE |
| 27 | RN7SL2--RF00100 | 6 | 71 | INCL_NON_REF_SPLICE |
| 27 | RN7SL2--RF00100 | 6 | 71 | INCL_NON_REF_SPLICE |
| 27 | RN7SL2--RF00100 | 6 | 71 | INCL_NON_REF_SPLICE |
| 27 | RN7SL2--RF00100 | 6 | 71 | INCL_NON_REF_SPLICE |
| 27 | RN7SL2--RF00100 | 6 | 71 | INCL_NON_REF_SPLICE |
| 27 | RN7SL2--RF00100 | 6 | 71 | INCL_NON_REF_SPLICE |
| 49 | RN7SL2--RMRP | 4 | 81 | INCL_NON_REF_SPLICE |
| 49 | RN7SL2--RMRP | 3 | 81 | INCL_NON_REF_SPLICE |
| 49 | RN7SL2--RMRP | 3 | 18 | INCL_NON_REF_SPLICE |
| 49 | RN7SL2--RMRP | 3 | 18 | INCL_NON_REF_SPLICE |
| 34 | RN7SL2--RMRP | 20 | 208 | INCL_NON_REF_SPLICE |
| 34 | RN7SL2--RMRP | 7 | 204 | INCL_NON_REF_SPLICE |
| 34 | RN7SL2--RMRP | 4 | 204 | INCL_NON_REF_SPLICE |
| 34 | RN7SL2--RMRP | 5 | 198 | INCL_NON_REF_SPLICE |
| 34 | RN7SL2--RMRP | 11 | 173 | INCL_NON_REF_SPLICE |
| 34 | RN7SL2--RMRP | 3 | 204 | INCL_NON_REF_SPLICE |
| 34 | RN7SL2--RMRP | 6 | 171 | INCL_NON_REF_SPLICE |
| 34 | RN7SL2--RMRP | 15 | 46 | INCL_NON_REF_SPLICE |
| 34 | RN7SL2--RMRP | 12 | 46 | INCL_NON_REF_SPLICE |
| 34 | RN7SL2--RMRP | 9 | 46 | INCL_NON_REF_SPLICE |
| 34 | RN7SL2--RMRP | 8 | 46 | INCL_NON_REF_SPLICE |
| 34 | RN7SL2--RMRP | 8 | 46 | INCL_NON_REF_SPLICE |
| 34 | RN7SL2--RMRP | 8 | 46 | INCL_NON_REF_SPLICE |
| 34 | RN7SL2--RMRP | 8 | 46 | INCL_NON_REF_SPLICE |
| 34 | RN7SL2--RMRP | 7 | 46 | INCL_NON_REF_SPLICE |
| 34 | RN7SL2--RMRP | 7 | 46 | INCL_NON_REF_SPLICE |
| 34 | RN7SL2--RMRP | 7 | 45 | INCL_NON_REF_SPLICE |
| 34 | RN7SL2--RMRP | 6 | 46 | INCL_NON_REF_SPLICE |
| 34 | RN7SL2--RMRP | 6 | 46 | INCL_NON_REF_SPLICE |
| 34 | RN7SL2--RMRP | 5 | 46 | INCL_NON_REF_SPLICE |
| 34 | RN7SL2--RMRP | 5 | 46 | INCL_NON_REF_SPLICE |
| 34 | RN7SL2--RMRP | 5 | 46 | INCL_NON_REF_SPLICE |
| 34 | RN7SL2--RMRP | 5 | 46 | INCL_NON_REF_SPLICE |
| 34 | RN7SL2--RMRP | 5 | 46 | INCL_NON_REF_SPLICE |
| 34 | RN7SL2--RMRP | 4 | 46 | INCL_NON_REF_SPLICE |
| 34 | RN7SL2--RMRP | 4 | 46 | INCL_NON_REF_SPLICE |
| 34 | RN7SL2--RMRP | 4 | 46 | INCL_NON_REF_SPLICE |
| 34 | RN7SL2--RMRP | 4 | 46 | INCL_NON_REF_SPLICE |
| 34 | RN7SL2--RMRP | 4 | 46 | INCL_NON_REF_SPLICE |
| 34 | RN7SL2--RMRP | 4 | 46 | INCL_NON_REF_SPLICE |
| 34 | RN7SL2--RMRP | 3 | 46 | INCL_NON_REF_SPLICE |
| 34 | RN7SL2--RMRP | 3 | 46 | INCL_NON_REF_SPLICE |
| 34 | RN7SL2--RMRP | 3 | 46 | INCL_NON_REF_SPLICE |
| 34 | RN7SL2--RMRP | 3 | 46 | INCL_NON_REF_SPLICE |
| 34 | RN7SL2--RMRP | 3 | 46 | INCL_NON_REF_SPLICE |
| 34 | RN7SL2--RMRP | 3 | 46 | INCL_NON_REF_SPLICE |
| 34 | RN7SL2--RMRP | 3 | 46 | INCL_NON_REF_SPLICE |
| 34 | RN7SL2--RMRP | 3 | 46 | INCL_NON_REF_SPLICE |
| 34 | RN7SL2--RMRP | 3 | 46 | INCL_NON_REF_SPLICE |
| 34 | RN7SL2--RMRP | 3 | 46 | INCL_NON_REF_SPLICE |
| 34 | RN7SL2--RMRP | 3 | 46 | INCL_NON_REF_SPLICE |
| 34 | RN7SL2--RMRP | 3 | 46 | INCL_NON_REF_SPLICE |
| 34 | RN7SL2--RMRP | 3 | 46 | INCL_NON_REF_SPLICE |
| 27 | RN7SL2--RMRP | 26 | 51 | INCL_NON_REF_SPLICE |
| 27 | RN7SL2--RMRP | 15 | 51 | INCL_NON_REF_SPLICE |
| 27 | RN7SL2--RMRP | 24 | 12 | INCL_NON_REF_SPLICE |
| 27 | RN7SL2--RMRP | 12 | 51 | INCL_NON_REF_SPLICE |
| 27 | RN7SL2--RMRP | 9 | 51 | INCL_NON_REF_SPLICE |
| 27 | RN7SL2--RMRP | 11 | 40 | INCL_NON_REF_SPLICE |
| 27 | RN7SL2--RMRP | 8 | 51 | INCL_NON_REF_SPLICE |
| 27 | RN7SL2--RMRP | 7 | 51 | INCL_NON_REF_SPLICE |
| 27 | RN7SL2--RMRP | 6 | 51 | INCL_NON_REF_SPLICE |
| 27 | RN7SL2--RMRP | 5 | 51 | INCL_NON_REF_SPLICE |
| 27 | RN7SL2--RMRP | 4 | 51 | INCL_NON_REF_SPLICE |
| 27 | RN7SL2--RMRP | 3 | 51 | INCL_NON_REF_SPLICE |
| 27 | RN7SL2--RMRP | 3 | 51 | INCL_NON_REF_SPLICE |
| 27 | RN7SL2--RMRP | 3 | 51 | INCL_NON_REF_SPLICE |
| 27 | RN7SL2--RMRP | 3 | 51 | INCL_NON_REF_SPLICE |
| 30 | RN7SL2--RN7SK | 29 | 35 | INCL_NON_REF_SPLICE |
| 30 | RN7SL2--RN7SK | 14 | 35 | INCL_NON_REF_SPLICE |
| 30 | RN7SL2--RN7SK | 10 | 35 | INCL_NON_REF_SPLICE |
| 30 | RN7SL2--RN7SK | 9 | 35 | INCL_NON_REF_SPLICE |
| 30 | RN7SL2--RN7SK | 7 | 35 | INCL_NON_REF_SPLICE |
| 30 | RN7SL2--RN7SK | 7 | 35 | INCL_NON_REF_SPLICE |
| 30 | RN7SL2--RN7SK | 6 | 35 | INCL_NON_REF_SPLICE |
| 30 | RN7SL2--RN7SK | 6 | 35 | INCL_NON_REF_SPLICE |
| 30 | RN7SL2--RN7SK | 6 | 35 | INCL_NON_REF_SPLICE |
| 30 | RN7SL2--RN7SK | 5 | 35 | INCL_NON_REF_SPLICE |
| 30 | RN7SL2--RN7SK | 5 | 35 | INCL_NON_REF_SPLICE |
| 30 | RN7SL2--RN7SK | 5 | 35 | INCL_NON_REF_SPLICE |
| 30 | RN7SL2--RN7SK | 5 | 35 | INCL_NON_REF_SPLICE |
| 30 | RN7SL2--RN7SK | 4 | 35 | INCL_NON_REF_SPLICE |
| 30 | RN7SL2--RN7SK | 4 | 35 | INCL_NON_REF_SPLICE |
| 30 | RN7SL2--RN7SK | 3 | 35 | INCL_NON_REF_SPLICE |
| 30 | RN7SL2--RN7SK | 3 | 35 | INCL_NON_REF_SPLICE |
| 30 | RN7SL2--RN7SK | 3 | 35 | INCL_NON_REF_SPLICE |
| 30 | RN7SL2--RN7SK | 3 | 35 | INCL_NON_REF_SPLICE |
| 30 | RN7SL2--RN7SK | 3 | 35 | INCL_NON_REF_SPLICE |
| 30 | RN7SL2--RN7SK | 3 | 35 | INCL_NON_REF_SPLICE |
| 30 | RN7SL2--RN7SK | 3 | 35 | INCL_NON_REF_SPLICE |
| 30 | RN7SL2--RN7SK | 3 | 35 | INCL_NON_REF_SPLICE |
| 30 | RN7SL2--RN7SK | 3 | 35 | INCL_NON_REF_SPLICE |
| 30 | RN7SL2--RN7SK | 3 | 35 | INCL_NON_REF_SPLICE |
| 30 | RN7SL2--RN7SK | 3 | 35 | INCL_NON_REF_SPLICE |
| 30 | RN7SL2--RN7SK | 3 | 35 | INCL_NON_REF_SPLICE |
| 30 | RN7SL2--RN7SK | 3 | 35 | INCL_NON_REF_SPLICE |
| 30 | RN7SL2--RN7SK | 3 | 35 | INCL_NON_REF_SPLICE |
| 27 | RN7SL2--RN7SK | 56 | 71 | INCL_NON_REF_SPLICE |
| 27 | RN7SL2--RN7SK | 28 | 71 | INCL_NON_REF_SPLICE |
| 27 | RN7SL2--RN7SK | 25 | 71 | INCL_NON_REF_SPLICE |
| 27 | RN7SL2--RN7SK | 22 | 71 | INCL_NON_REF_SPLICE |
| 27 | RN7SL2--RN7SK | 21 | 71 | INCL_NON_REF_SPLICE |
| 27 | RN7SL2--RN7SK | 18 | 71 | INCL_NON_REF_SPLICE |
| 27 | RN7SL2--RN7SK | 18 | 71 | INCL_NON_REF_SPLICE |
| 27 | RN7SL2--RN7SK | 14 | 71 | INCL_NON_REF_SPLICE |
| 27 | RN7SL2--RN7SK | 13 | 71 | INCL_NON_REF_SPLICE |
| 27 | RN7SL2--RN7SK | 11 | 71 | INCL_NON_REF_SPLICE |
| 27 | RN7SL2--RN7SK | 11 | 71 | INCL_NON_REF_SPLICE |
| 27 | RN7SL2--RN7SK | 11 | 71 | INCL_NON_REF_SPLICE |
| 27 | RN7SL2--RN7SK | 11 | 71 | INCL_NON_REF_SPLICE |
| 27 | RN7SL2--RN7SK | 11 | 71 | INCL_NON_REF_SPLICE |
| 27 | RN7SL2--RN7SK | 11 | 71 | INCL_NON_REF_SPLICE |
| 27 | RN7SL2--RN7SK | 9 | 71 | INCL_NON_REF_SPLICE |
| 27 | RN7SL2--RN7SK | 8 | 71 | INCL_NON_REF_SPLICE |
| 27 | RN7SL2--RN7SK | 8 | 71 | INCL_NON_REF_SPLICE |
| 27 | RN7SL2--RN7SK | 8 | 71 | INCL_NON_REF_SPLICE |
| 27 | RN7SL2--RN7SK | 8 | 71 | INCL_NON_REF_SPLICE |
| 27 | RN7SL2--RN7SK | 8 | 71 | INCL_NON_REF_SPLICE |
| 27 | RN7SL2--RN7SK | 7 | 71 | INCL_NON_REF_SPLICE |
| 27 | RN7SL2--RN7SK | 7 | 71 | INCL_NON_REF_SPLICE |
| 27 | RN7SL2--RN7SK | 7 | 71 | INCL_NON_REF_SPLICE |
| 27 | RN7SL2--RN7SK | 7 | 71 | INCL_NON_REF_SPLICE |
| 27 | RN7SL2--RN7SK | 7 | 71 | INCL_NON_REF_SPLICE |
| 27 | RN7SL2--RN7SK | 7 | 71 | INCL_NON_REF_SPLICE |
| 27 | RN7SL2--RN7SK | 7 | 71 | INCL_NON_REF_SPLICE |
| 27 | RN7SL2--RN7SK | 7 | 71 | INCL_NON_REF_SPLICE |
| 27 | RN7SL2--RN7SK | 7 | 71 | INCL_NON_REF_SPLICE |
| 27 | RN7SL2--RN7SK | 6 | 71 | INCL_NON_REF_SPLICE |
| 27 | RN7SL2--RN7SK | 6 | 71 | INCL_NON_REF_SPLICE |
| 27 | RN7SL2--RN7SK | 6 | 71 | INCL_NON_REF_SPLICE |
| 27 | RN7SL2--RN7SK | 6 | 71 | INCL_NON_REF_SPLICE |
| 27 | RN7SL2--RN7SK | 6 | 71 | INCL_NON_REF_SPLICE |
| 27 | RN7SL2--RN7SK | 6 | 71 | INCL_NON_REF_SPLICE |
| 27 | RN7SL2--RN7SK | 6 | 71 | INCL_NON_REF_SPLICE |
| 27 | RN7SL2--RN7SK | 6 | 71 | INCL_NON_REF_SPLICE |
| 27 | RN7SL2--RN7SK | 6 | 71 | INCL_NON_REF_SPLICE |
| 27 | RN7SL2--RNU4-2 | 38 | 0 | INCL_NON_REF_SPLICE |
| 27 | RN7SL2--RNU5A-1 | 32 | 0 | INCL_NON_REF_SPLICE |
| 30 | RN7SL2--RPPH1 | 13 | 18 | INCL_NON_REF_SPLICE |
| 30 | RN7SL2--RPPH1 | 4 | 18 | INCL_NON_REF_SPLICE |
| 30 | RN7SL2--RPPH1 | 4 | 18 | INCL_NON_REF_SPLICE |
| 30 | RN7SL2--RPPH1 | 3 | 18 | INCL_NON_REF_SPLICE |
| 30 | RN7SL2--RPPH1 | 3 | 18 | INCL_NON_REF_SPLICE |
| 30 | RN7SL2--RPPH1 | 3 | 18 | INCL_NON_REF_SPLICE |
| 30 | RN7SL2--RPPH1 | 3 | 18 | INCL_NON_REF_SPLICE |
| 30 | RN7SL2--RPPH1 | 3 | 18 | INCL_NON_REF_SPLICE |
| 30 | RN7SL2--RPPH1 | 3 | 18 | INCL_NON_REF_SPLICE |
| 49 | RN7SL2--RPPH1 | 4 | 84 | INCL_NON_REF_SPLICE |
| 34 | RN7SL2--RPPH1 | 16 | 90 | INCL_NON_REF_SPLICE |
| 34 | RN7SL2--RPPH1 | 8 | 90 | INCL_NON_REF_SPLICE |
| 34 | RN7SL2--RPPH1 | 4 | 90 | INCL_NON_REF_SPLICE |
| 34 | RN7SL2--RPPH1 | 4 | 90 | INCL_NON_REF_SPLICE |
| 34 | RN7SL2--RPPH1 | 3 | 90 | INCL_NON_REF_SPLICE |
| 34 | RN7SL2--RPPH1 | 3 | 90 | INCL_NON_REF_SPLICE |
| 34 | RN7SL2--RPPH1 | 3 | 90 | INCL_NON_REF_SPLICE |
| 34 | RN7SL2--RPPH1 | 3 | 90 | INCL_NON_REF_SPLICE |
| 34 | RN7SL2--RPPH1 | 3 | 90 | INCL_NON_REF_SPLICE |
| 34 | RN7SL2--RPPH1 | 3 | 90 | INCL_NON_REF_SPLICE |
| 34 | RN7SL2--RPPH1 | 3 | 90 | INCL_NON_REF_SPLICE |
| 34 | RN7SL2--RPPH1 | 3 | 90 | INCL_NON_REF_SPLICE |
| 27 | RN7SL2--RPPH1 | 54 | 82 | INCL_NON_REF_SPLICE |
| 27 | RN7SL2--RPPH1 | 20 | 82 | INCL_NON_REF_SPLICE |
| 27 | RN7SL2--RPPH1 | 17 | 82 | INCL_NON_REF_SPLICE |
| 27 | RN7SL2--RPPH1 | 15 | 82 | INCL_NON_REF_SPLICE |
| 27 | RN7SL2--RPPH1 | 14 | 82 | INCL_NON_REF_SPLICE |
| 27 | RN7SL2--RPPH1 | 13 | 82 | INCL_NON_REF_SPLICE |
| 27 | RN7SL2--RPPH1 | 10 | 82 | INCL_NON_REF_SPLICE |
| 27 | RN7SL2--RPPH1 | 10 | 82 | INCL_NON_REF_SPLICE |
| 27 | RN7SL2--RPPH1 | 9 | 82 | INCL_NON_REF_SPLICE |
| 27 | RN7SL2--RPPH1 | 9 | 82 | INCL_NON_REF_SPLICE |
| 27 | RN7SL2--RPPH1 | 9 | 82 | INCL_NON_REF_SPLICE |
| 27 | RN7SL2--RPPH1 | 9 | 82 | INCL_NON_REF_SPLICE |
| 27 | RN7SL2--RPPH1 | 7 | 82 | INCL_NON_REF_SPLICE |
| 27 | RN7SL2--RPPH1 | 7 | 82 | INCL_NON_REF_SPLICE |
| 27 | RN7SL2--RPPH1 | 7 | 82 | INCL_NON_REF_SPLICE |
| 27 | RN7SL2--SNORA73B | 27 | 3 | INCL_NON_REF_SPLICE |
| 34 | RN7SL3--AC007952.4 | 6 | 0 | INCL_NON_REF_SPLICE |
| 30 | RN7SL4P--RNU4-2 | 12 | 0 | INCL_NON_REF_SPLICE |
| 27 | RN7SL5P--AC005005.4 | 8 | 0 | INCL_NON_REF_SPLICE |
| 48 | RN7SL5P--IFI6 | 15 | 1 | INCL_NON_REF_SPLICE |
| 49 | RNA5-8SN2--AL355075.4 | 3 | 2 | INCL_NON_REF_SPLICE |
| 49 | RNA5-8SN2--AL355075.4 | 3 | 2 | INCL_NON_REF_SPLICE |
| 49 | RNA5-8SN2--AL355075.4 | 3 | 2 | INCL_NON_REF_SPLICE |
| 49 | RNA5-8SN2--AL355075.4 | 3 | 2 | INCL_NON_REF_SPLICE |
| 49 | RNA5-8SN2--RPPH1 | 3 | 2 | INCL_NON_REF_SPLICE |
| 49 | RNA5-8SN2--RPPH1 | 3 | 2 | INCL_NON_REF_SPLICE |
| 49 | RNA5-8SN2--RPPH1 | 3 | 2 | INCL_NON_REF_SPLICE |
| 49 | RNA5-8SN2--RPPH1 | 3 | 2 | INCL_NON_REF_SPLICE |
| 34 | RNA5-8SN2--SNHG3 | 11 | 2 | INCL_NON_REF_SPLICE |
| 34 | RNA5-8SN2--SNHG3 | 11 | 2 | INCL_NON_REF_SPLICE |
| 34 | RNA5-8SN2--SNHG3 | 9 | 2 | INCL_NON_REF_SPLICE |
| 34 | RNA5-8SN2--SNHG3 | 9 | 2 | INCL_NON_REF_SPLICE |
| 49 | RNA5-8SN2--SNORA73B | 4 | 0 | INCL_NON_REF_SPLICE |
| 27 | RNU1-1--RF00100 | 28 | 0 | INCL_NON_REF_SPLICE |
| 27 | RNU1-1--SNORA8 | 5 | 0 | INCL_NON_REF_SPLICE |
| 27 | RNU1-1--TAF1D | 5 | 0 | INCL_NON_REF_SPLICE |
| 20 | RNU1-27P--AL139099.4 | 12 | 10 | INCL_NON_REF_SPLICE |
| 20 | RNU1-27P--AL139099.4 | 10 | 10 | INCL_NON_REF_SPLICE |
| 20 | RNU1-27P--AL139099.4 | 9 | 10 | INCL_NON_REF_SPLICE |
| 20 | RNU1-27P--AL139099.4 | 9 | 10 | INCL_NON_REF_SPLICE |
| 20 | RNU1-27P--AL139099.4 | 9 | 10 | INCL_NON_REF_SPLICE |
| 20 | RNU1-27P--AL139099.4 | 8 | 10 | INCL_NON_REF_SPLICE |
| 20 | RNU1-27P--AL139099.4 | 8 | 10 | INCL_NON_REF_SPLICE |
| 20 | RNU1-27P--AL139099.4 | 6 | 10 | INCL_NON_REF_SPLICE |
| 20 | RNU1-27P--AL139099.4 | 5 | 10 | INCL_NON_REF_SPLICE |
| 20 | RNU1-27P--AL139099.4 | 4 | 10 | INCL_NON_REF_SPLICE |
| 20 | RNU1-27P--AL139099.4 | 4 | 10 | INCL_NON_REF_SPLICE |
| 20 | RNU1-27P--AL139099.4 | 4 | 10 | INCL_NON_REF_SPLICE |
| 20 | RNU1-27P--AL139099.4 | 4 | 10 | INCL_NON_REF_SPLICE |
| 20 | RNU1-27P--AL139099.4 | 3 | 10 | INCL_NON_REF_SPLICE |
| 20 | RNU1-27P--AL139099.4 | 3 | 10 | INCL_NON_REF_SPLICE |
| 20 | RNU1-27P--AL139099.4 | 3 | 10 | INCL_NON_REF_SPLICE |
| 20 | RNU1-27P--AL139099.4 | 3 | 10 | INCL_NON_REF_SPLICE |
| 20 | RNU1-27P--AL139099.4 | 3 | 10 | INCL_NON_REF_SPLICE |
| 20 | RNU1-27P--AL139099.4 | 3 | 10 | INCL_NON_REF_SPLICE |
| 20 | RNU1-27P--AL139099.4 | 3 | 10 | INCL_NON_REF_SPLICE |
| 20 | RNU1-27P--AL139099.4 | 3 | 10 | INCL_NON_REF_SPLICE |
| 20 | RNU1-27P--AL355075.4 | 6 | 3 | INCL_NON_REF_SPLICE |
| 20 | RNU1-27P--AL355075.4 | 5 | 3 | INCL_NON_REF_SPLICE |
| 20 | RNU1-27P--AL355075.4 | 3 | 3 | INCL_NON_REF_SPLICE |
| 20 | RNU1-27P--RMRP | 10 | 2 | INCL_NON_REF_SPLICE |
| 20 | RNU1-27P--RMRP | 7 | 2 | INCL_NON_REF_SPLICE |
| 20 | RNU1-27P--RMRP | 4 | 2 | INCL_NON_REF_SPLICE |
| 20 | RNU1-27P--RMRP | 3 | 2 | INCL_NON_REF_SPLICE |
| 20 | RNU1-27P--RMRP | 3 | 1 | INCL_NON_REF_SPLICE |
| 20 | RNU1-27P--RMRP | 3 | 1 | INCL_NON_REF_SPLICE |
| 20 | RNU1-27P--RMRP | 3 | 1 | INCL_NON_REF_SPLICE |
| 20 | RNU1-27P--RMRP | 3 | 1 | INCL_NON_REF_SPLICE |
| 20 | RNU1-27P--RMRP | 3 | 1 | INCL_NON_REF_SPLICE |
| 20 | RNU1-27P--RMRP | 3 | 1 | INCL_NON_REF_SPLICE |
| 20 | RNU1-27P--RN7SL1 | 12 | 10 | INCL_NON_REF_SPLICE |
| 20 | RNU1-27P--RN7SL1 | 10 | 10 | INCL_NON_REF_SPLICE |
| 20 | RNU1-27P--RN7SL1 | 9 | 10 | INCL_NON_REF_SPLICE |
| 20 | RNU1-27P--RN7SL1 | 9 | 10 | INCL_NON_REF_SPLICE |
| 20 | RNU1-27P--RN7SL1 | 9 | 10 | INCL_NON_REF_SPLICE |
| 20 | RNU1-27P--RN7SL1 | 8 | 10 | INCL_NON_REF_SPLICE |
| 20 | RNU1-27P--RN7SL1 | 8 | 10 | INCL_NON_REF_SPLICE |
| 20 | RNU1-27P--RN7SL1 | 6 | 10 | INCL_NON_REF_SPLICE |
| 20 | RNU1-27P--RN7SL1 | 5 | 10 | INCL_NON_REF_SPLICE |
| 20 | RNU1-27P--RN7SL1 | 4 | 10 | INCL_NON_REF_SPLICE |
| 20 | RNU1-27P--RN7SL1 | 4 | 10 | INCL_NON_REF_SPLICE |
| 20 | RNU1-27P--RN7SL1 | 4 | 10 | INCL_NON_REF_SPLICE |
| 20 | RNU1-27P--RN7SL1 | 4 | 10 | INCL_NON_REF_SPLICE |
| 20 | RNU1-27P--RN7SL1 | 3 | 10 | INCL_NON_REF_SPLICE |
| 20 | RNU1-27P--RN7SL1 | 3 | 10 | INCL_NON_REF_SPLICE |
| 20 | RNU1-27P--RN7SL1 | 3 | 10 | INCL_NON_REF_SPLICE |
| 20 | RNU1-27P--RN7SL1 | 3 | 10 | INCL_NON_REF_SPLICE |
| 20 | RNU1-27P--RN7SL1 | 3 | 10 | INCL_NON_REF_SPLICE |
| 20 | RNU1-27P--RN7SL1 | 3 | 10 | INCL_NON_REF_SPLICE |
| 20 | RNU1-27P--RN7SL1 | 3 | 10 | INCL_NON_REF_SPLICE |
| 20 | RNU1-27P--RN7SL1 | 3 | 10 | INCL_NON_REF_SPLICE |
| 20 | RNU1-27P--RPPH1 | 6 | 3 | INCL_NON_REF_SPLICE |
| 20 | RNU1-27P--RPPH1 | 5 | 3 | INCL_NON_REF_SPLICE |
| 20 | RNU1-27P--RPPH1 | 3 | 3 | INCL_NON_REF_SPLICE |
| 27 | RNU1-4--RMRP | 27 | 0 | INCL_NON_REF_SPLICE |
| 27 | RNU1-4--RMRP | 24 | 0 | INCL_NON_REF_SPLICE |
| 27 | RNU1-4--RMRP | 16 | 0 | INCL_NON_REF_SPLICE |
| 20 | RNU4-1--RN7SL3 | 5 | 0 | INCL_NON_REF_SPLICE |
| 32 | RNU4-2--AL139099.4 | 4 | 0 | INCL_NON_REF_SPLICE |
| 32 | RNU4-2--AL355075.4 | 4 | 0 | INCL_NON_REF_SPLICE |
| 27 | RNU4-2--RF00003 | 14 | 0 | INCL_NON_REF_SPLICE |
| 27 | RNU4-2--RF00003 | 11 | 0 | INCL_NON_REF_SPLICE |
| 32 | RNU4-2--RN7SL1 | 4 | 0 | INCL_NON_REF_SPLICE |
| 32 | RNU4-2--RPPH1 | 4 | 0 | INCL_NON_REF_SPLICE |
| 27 | RNU5A-1--SNORA73A | 10 | 0 | INCL_NON_REF_SPLICE |
| 27 | RNU5B-1--RF00003 | 16 | 0 | INCL_NON_REF_SPLICE |
| 27 | RNU5B-1--RF00003 | 12 | 0 | INCL_NON_REF_SPLICE |
| 30 | RNU5B-1--RN7SKP203 | 5 | 0 | INCL_NON_REF_SPLICE |
| 27 | RNU5B-1--SNORA73B | 8 | 0 | INCL_NON_REF_SPLICE |
| 44 | RNY3P8--ABCC4 | 3 | 0 | INCL_NON_REF_SPLICE |
| 41 | RP11-1143G9.4--RN7SL2 | 4 | 2 | INCL_NON_REF_SPLICE |
| 52 | RP11-123K3.4--SHMT2 | 4 | 1 | INCL_NON_REF_SPLICE |
| 52 | RP11-163N6.2--C6 | 10 | 0 | ONLY_REF_SPLICE |
| 41 | RP11-290D2.6--MTND2P28 | 4 | 2 | INCL_NON_REF_SPLICE |
| 41 | RP11-96H19.1--RP11-446N19.1 | 5 | 0 | ONLY_REF_SPLICE |
| 52 | RPH3A--RP11-19O2.2 | 12 | 0 | ONLY_REF_SPLICE |
| 24 | RPL13--RPS18 | 4 | 1 | INCL_NON_REF_SPLICE |
| 28 | RPL18A--HBA1 | 5 | 0 | INCL_NON_REF_SPLICE |
| 25 | RPL18A--HBA1 | 4 | 2 | INCL_NON_REF_SPLICE |
| 24 | RPL18A--HBA1 | 11 | 0 | INCL_NON_REF_SPLICE |
| 38 | RPL21--CEP72 | 6 | 0 | INCL_NON_REF_SPLICE |
| 24 | RPL3--EEF1A1 | 5 | 2 | INCL_NON_REF_SPLICE |
| 52 | RPL6--ATF6 | 11 | 0 | INCL_NON_REF_SPLICE |
| 24 | RPLP0P6--EEF1A1P5 | 3 | 2 | INCL_NON_REF_SPLICE |
| 55 | RPLP2--TGIF2 | 8 | 0 | INCL_NON_REF_SPLICE |
| 46 | RPP14--RN7SL2 | 16 | 0 | INCL_NON_REF_SPLICE |
| 34 | RPPH1--AC007952.4 | 6 | 0 | INCL_NON_REF_SPLICE |
| 49 | RPPH1--AL139099.4 | 3 | 72 | INCL_NON_REF_SPLICE |
| 30 | RPPH1--PLCG2 | 3 | 3 | INCL_NON_REF_SPLICE |
| 34 | RPPH1--PLCG2 | 4 | 30 | INCL_NON_REF_SPLICE |
| 34 | RPPH1--PLCG2 | 4 | 30 | INCL_NON_REF_SPLICE |
| 27 | RPPH1--PLCG2 | 14 | 19 | INCL_NON_REF_SPLICE |
| 30 | RPPH1--RF00100 | 3 | 10 | INCL_NON_REF_SPLICE |
| 30 | RPPH1--RF00100 | 3 | 10 | INCL_NON_REF_SPLICE |
| 30 | RPPH1--RF00100 | 3 | 10 | INCL_NON_REF_SPLICE |
| 34 | RPPH1--RF00100 | 7 | 70 | INCL_NON_REF_SPLICE |
| 34 | RPPH1--RF00100 | 5 | 70 | INCL_NON_REF_SPLICE |
| 34 | RPPH1--RF00100 | 4 | 70 | INCL_NON_REF_SPLICE |
| 34 | RPPH1--RF00100 | 4 | 70 | INCL_NON_REF_SPLICE |
| 27 | RPPH1--RF00100 | 15 | 45 | INCL_NON_REF_SPLICE |
| 27 | RPPH1--RF00100 | 7 | 45 | INCL_NON_REF_SPLICE |
| 27 | RPPH1--RF00100 | 7 | 45 | INCL_NON_REF_SPLICE |
| 27 | RPPH1--RF00100 | 6 | 45 | INCL_NON_REF_SPLICE |
| 27 | RPPH1--RF00100 | 6 | 45 | INCL_NON_REF_SPLICE |
| 27 | RPPH1--RF00100 | 6 | 45 | INCL_NON_REF_SPLICE |
| 27 | RPPH1--RF00100 | 4 | 45 | INCL_NON_REF_SPLICE |
| 27 | RPPH1--RF00100 | 4 | 45 | INCL_NON_REF_SPLICE |
| 27 | RPPH1--RF00100 | 4 | 45 | INCL_NON_REF_SPLICE |
| 27 | RPPH1--RF00100 | 4 | 45 | INCL_NON_REF_SPLICE |
| 27 | RPPH1--RF00100 | 4 | 45 | INCL_NON_REF_SPLICE |
| 27 | RPPH1--RF00100 | 4 | 45 | INCL_NON_REF_SPLICE |
| 27 | RPPH1--RF00100 | 3 | 45 | INCL_NON_REF_SPLICE |
| 27 | RPPH1--RF00100 | 3 | 45 | INCL_NON_REF_SPLICE |
| 27 | RPPH1--RF00100 | 3 | 45 | INCL_NON_REF_SPLICE |
| 27 | RPPH1--RF00100 | 3 | 45 | INCL_NON_REF_SPLICE |
| 27 | RPPH1--RF00100 | 3 | 45 | INCL_NON_REF_SPLICE |
| 27 | RPPH1--RF00100 | 3 | 45 | INCL_NON_REF_SPLICE |
| 27 | RPPH1--RF00100 | 3 | 45 | INCL_NON_REF_SPLICE |
| 27 | RPPH1--RF00100 | 3 | 45 | INCL_NON_REF_SPLICE |
| 27 | RPPH1--RF00100 | 3 | 45 | INCL_NON_REF_SPLICE |
| 27 | RPPH1--RF00100 | 3 | 45 | INCL_NON_REF_SPLICE |
| 27 | RPPH1--RF00100 | 3 | 45 | INCL_NON_REF_SPLICE |
| 30 | RPPH1--RN7SK | 3 | 10 | INCL_NON_REF_SPLICE |
| 30 | RPPH1--RN7SK | 3 | 10 | INCL_NON_REF_SPLICE |
| 30 | RPPH1--RN7SK | 3 | 10 | INCL_NON_REF_SPLICE |
| 34 | RPPH1--RN7SK | 7 | 70 | INCL_NON_REF_SPLICE |
| 34 | RPPH1--RN7SK | 5 | 70 | INCL_NON_REF_SPLICE |
| 34 | RPPH1--RN7SK | 4 | 70 | INCL_NON_REF_SPLICE |
| 34 | RPPH1--RN7SK | 4 | 70 | INCL_NON_REF_SPLICE |
| 27 | RPPH1--RN7SK | 15 | 45 | INCL_NON_REF_SPLICE |
| 27 | RPPH1--RN7SK | 7 | 45 | INCL_NON_REF_SPLICE |
| 27 | RPPH1--RN7SK | 7 | 45 | INCL_NON_REF_SPLICE |
| 27 | RPPH1--RN7SK | 6 | 45 | INCL_NON_REF_SPLICE |
| 27 | RPPH1--RN7SK | 6 | 45 | INCL_NON_REF_SPLICE |
| 27 | RPPH1--RN7SK | 6 | 45 | INCL_NON_REF_SPLICE |
| 27 | RPPH1--RN7SK | 4 | 45 | INCL_NON_REF_SPLICE |
| 27 | RPPH1--RN7SK | 4 | 45 | INCL_NON_REF_SPLICE |
| 27 | RPPH1--RN7SK | 4 | 45 | INCL_NON_REF_SPLICE |
| 27 | RPPH1--RN7SK | 4 | 45 | INCL_NON_REF_SPLICE |
| 27 | RPPH1--RN7SK | 4 | 45 | INCL_NON_REF_SPLICE |
| 27 | RPPH1--RN7SK | 4 | 45 | INCL_NON_REF_SPLICE |
| 27 | RPPH1--RN7SK | 3 | 45 | INCL_NON_REF_SPLICE |
| 27 | RPPH1--RN7SK | 3 | 45 | INCL_NON_REF_SPLICE |
| 27 | RPPH1--RN7SK | 3 | 45 | INCL_NON_REF_SPLICE |
| 27 | RPPH1--RN7SK | 3 | 45 | INCL_NON_REF_SPLICE |
| 27 | RPPH1--RN7SK | 3 | 45 | INCL_NON_REF_SPLICE |
| 27 | RPPH1--RN7SK | 3 | 45 | INCL_NON_REF_SPLICE |
| 27 | RPPH1--RN7SK | 3 | 45 | INCL_NON_REF_SPLICE |
| 27 | RPPH1--RN7SK | 3 | 45 | INCL_NON_REF_SPLICE |
| 27 | RPPH1--RN7SK | 3 | 45 | INCL_NON_REF_SPLICE |
| 27 | RPPH1--RN7SK | 3 | 45 | INCL_NON_REF_SPLICE |
| 27 | RPPH1--RN7SK | 3 | 45 | INCL_NON_REF_SPLICE |
| 49 | RPPH1--RN7SL1 | 3 | 72 | INCL_NON_REF_SPLICE |
| 34 | RPPH1--RN7SL2 | 8 | 90 | INCL_NON_REF_SPLICE |
| 34 | RPPH1--RN7SL2 | 4 | 90 | INCL_NON_REF_SPLICE |
| 34 | RPPH1--RN7SL2 | 3 | 90 | INCL_NON_REF_SPLICE |
| 34 | RPPH1--RN7SL2 | 3 | 90 | INCL_NON_REF_SPLICE |
| 34 | RPPH1--RN7SL2 | 3 | 90 | INCL_NON_REF_SPLICE |
| 34 | RPPH1--RN7SL2 | 3 | 90 | INCL_NON_REF_SPLICE |
| 30 | RPPH1--RN7SL3 | 4 | 10 | INCL_NON_REF_SPLICE |
| 30 | RPPH1--RN7SL3 | 3 | 10 | INCL_NON_REF_SPLICE |
| 30 | RPPH1--RN7SL3 | 3 | 10 | INCL_NON_REF_SPLICE |
| 27 | RPPH1--RN7SL3 | 10 | 82 | INCL_NON_REF_SPLICE |
| 27 | RPPH1--RN7SL3 | 9 | 82 | INCL_NON_REF_SPLICE |
| 27 | RPPH1--RN7SL3 | 7 | 82 | INCL_NON_REF_SPLICE |
| 27 | RPPH1--RN7SL3 | 7 | 82 | INCL_NON_REF_SPLICE |
| 27 | RPPH1--RN7SL3 | 7 | 82 | INCL_NON_REF_SPLICE |
| 27 | RPPH1--RN7SL3 | 6 | 82 | INCL_NON_REF_SPLICE |
| 27 | RPPH1--RN7SL3 | 5 | 82 | INCL_NON_REF_SPLICE |
| 27 | RPPH1--RN7SL3 | 5 | 82 | INCL_NON_REF_SPLICE |
| 27 | RPPH1--RN7SL3 | 5 | 82 | INCL_NON_REF_SPLICE |
| 27 | RPPH1--RN7SL3 | 5 | 82 | INCL_NON_REF_SPLICE |
| 27 | RPPH1--RN7SL3 | 5 | 82 | INCL_NON_REF_SPLICE |
| 27 | RPPH1--RN7SL3 | 5 | 82 | INCL_NON_REF_SPLICE |
| 27 | RPPH1--RN7SL3 | 5 | 82 | INCL_NON_REF_SPLICE |
| 27 | RPPH1--RN7SL3 | 5 | 82 | INCL_NON_REF_SPLICE |
| 27 | RPPH1--RN7SL3 | 4 | 82 | INCL_NON_REF_SPLICE |
| 27 | RPPH1--RN7SL3 | 4 | 82 | INCL_NON_REF_SPLICE |
| 27 | RPPH1--RN7SL3 | 4 | 82 | INCL_NON_REF_SPLICE |
| 27 | RPPH1--RN7SL3 | 3 | 82 | INCL_NON_REF_SPLICE |
| 27 | RPPH1--RN7SL3 | 3 | 82 | INCL_NON_REF_SPLICE |
| 27 | RPPH1--RN7SL3 | 3 | 82 | INCL_NON_REF_SPLICE |
| 27 | RPPH1--RN7SL3 | 3 | 82 | INCL_NON_REF_SPLICE |
| 27 | RPPH1--RN7SL3 | 3 | 82 | INCL_NON_REF_SPLICE |
| 27 | RPPH1--RN7SL3 | 3 | 82 | INCL_NON_REF_SPLICE |
| 27 | RPPH1--RN7SL3 | 3 | 82 | INCL_NON_REF_SPLICE |
| 27 | RPPH1--RN7SL3 | 3 | 82 | INCL_NON_REF_SPLICE |
| 27 | RPPH1--RN7SL3 | 3 | 82 | INCL_NON_REF_SPLICE |
| 27 | RPPH1--RN7SL3 | 3 | 82 | INCL_NON_REF_SPLICE |
| 27 | RPPH1--RN7SL3 | 3 | 82 | INCL_NON_REF_SPLICE |
| 27 | RPPH1--RN7SL3 | 3 | 82 | INCL_NON_REF_SPLICE |
| 27 | RPPH1--RN7SL3 | 3 | 82 | INCL_NON_REF_SPLICE |
| 27 | RPPH1--RN7SL3 | 3 | 82 | INCL_NON_REF_SPLICE |
| 27 | RPPH1--RN7SL3 | 3 | 82 | INCL_NON_REF_SPLICE |
| 27 | RPPH1--RN7SL3 | 3 | 82 | INCL_NON_REF_SPLICE |
| 27 | RPPH1--RN7SL3 | 3 | 82 | INCL_NON_REF_SPLICE |
| 32 | RPPH1--RN7SL4P | 3 | 1 | INCL_NON_REF_SPLICE |
| 34 | RPPH1--RNU4-1 | 5 | 0 | INCL_NON_REF_SPLICE |
| 28 | RPS6--AL136231.1 | 7 | 0 | INCL_NON_REF_SPLICE |
| 24 | RPS6--AL136231.1 | 8 | 0 | INCL_NON_REF_SPLICE |
| 47 | RUNX1--RUNX1T1 | 49 | 3 | ONLY_REF_SPLICE |
| 47 | RUNX1--RUNX1T1 | 7 | 3 | ONLY_REF_SPLICE |
| 31 | RUNX1--RUNX1T1 | 77 | 6 | ONLY_REF_SPLICE |
| 31 | RUNX1--RUNX1T1 | 8 | 6 | ONLY_REF_SPLICE |
| 34 | RUNX1--RUNX1T1 | 133 | 6 | ONLY_REF_SPLICE |
| 48 | RUNX1--RUNX1T1 | 127 | 6 | ONLY_REF_SPLICE |
| 48 | RUNX1--RUNX1T1 | 34 | 6 | ONLY_REF_SPLICE |
| 20 | RUNX1--RUNX1T1 | 35 | 0 | ONLY_REF_SPLICE |
| 20 | RUNX1--RUNX1T1 | 4 | 0 | ONLY_REF_SPLICE |
| 45 | RUNX1--RUNX1T1 | 52 | 2 | ONLY_REF_SPLICE |
| 45 | SCARNA10--PTCH2 | 5 | 0 | INCL_NON_REF_SPLICE |
| 45 | SCARNA10--RNU5A-1 | 9 | 0 | INCL_NON_REF_SPLICE |
| 55 | SCD--FAM175A | 6 | 0 | INCL_NON_REF_SPLICE |
| 32 | SLC7A5--SMG1 | 8 | 0 | ONLY_REF_SPLICE |
| 49 | SLC7A5--SMG1 | 5 | 2 | ONLY_REF_SPLICE |
| 47 | SLC7A5--SMG1 | 10 | 0 | ONLY_REF_SPLICE |
| 20 | SLC7A5--SMG1 | 20 | 0 | ONLY_REF_SPLICE |
| 55 | SMARCD2--BRF2 | 5 | 0 | INCL_NON_REF_SPLICE |
| 34 | SNHG3--PLCG2 | 25 | 10 | INCL_NON_REF_SPLICE |
| 34 | SNHG3--RF00003 | 4 | 1 | INCL_NON_REF_SPLICE |
| 34 | SNHG3--RF00003 | 4 | 1 | INCL_NON_REF_SPLICE |
| 34 | SNHG3--RNA5-8SN2 | 23 | 1 | INCL_NON_REF_SPLICE |
| 34 | SNHG3--RNA5-8SN2 | 23 | 1 | INCL_NON_REF_SPLICE |
| 34 | SNHG3--RNA5-8SN2 | 20 | 2 | INCL_NON_REF_SPLICE |
| 34 | SNHG3--RNA5-8SN2 | 20 | 1 | INCL_NON_REF_SPLICE |
| 34 | SNHG3--RNA5-8SN2 | 8 | 2 | INCL_NON_REF_SPLICE |
| 27 | SNHG3--RNU1-1 | 11 | 0 | INCL_NON_REF_SPLICE |
| 27 | SNHG3--RNU1-1 | 7 | 0 | INCL_NON_REF_SPLICE |
| 20 | SNORA48--RNU1-1 | 8 | 0 | INCL_NON_REF_SPLICE |
| 20 | SNORA48--RNU1-1 | 5 | 0 | INCL_NON_REF_SPLICE |
| 20 | SNORA63--RF00003 | 4 | 0 | INCL_NON_REF_SPLICE |
| 27 | SNORA63--RNU4-1 | 6 | 0 | INCL_NON_REF_SPLICE |
| 34 | SNORA73A--AL355075.4 | 16 | 13 | INCL_NON_REF_SPLICE |
| 34 | SNORA73A--PLCG2 | 25 | 10 | INCL_NON_REF_SPLICE |
| 34 | SNORA73A--RF00100 | 28 | 40 | INCL_NON_REF_SPLICE |
| 34 | SNORA73A--RF00100 | 22 | 40 | INCL_NON_REF_SPLICE |
| 34 | SNORA73A--RF00100 | 17 | 40 | INCL_NON_REF_SPLICE |
| 34 | SNORA73A--RF00100 | 12 | 40 | INCL_NON_REF_SPLICE |
| 34 | SNORA73A--RF00100 | 10 | 40 | INCL_NON_REF_SPLICE |
| 34 | SNORA73A--RF00100 | 9 | 40 | INCL_NON_REF_SPLICE |
| 34 | SNORA73A--RF00100 | 9 | 40 | INCL_NON_REF_SPLICE |
| 34 | SNORA73A--RF00100 | 8 | 40 | INCL_NON_REF_SPLICE |
| 34 | SNORA73A--RF00100 | 7 | 40 | INCL_NON_REF_SPLICE |
| 34 | SNORA73A--RF00100 | 5 | 40 | INCL_NON_REF_SPLICE |
| 34 | SNORA73A--RF00100 | 5 | 40 | INCL_NON_REF_SPLICE |
| 34 | SNORA73A--RF00100 | 3 | 40 | INCL_NON_REF_SPLICE |
| 34 | SNORA73A--RF00100 | 3 | 40 | INCL_NON_REF_SPLICE |
| 34 | SNORA73A--RF00100 | 3 | 40 | INCL_NON_REF_SPLICE |
| 34 | SNORA73A--RF00100 | 3 | 40 | INCL_NON_REF_SPLICE |
| 34 | SNORA73A--RF00100 | 3 | 40 | INCL_NON_REF_SPLICE |
| 34 | SNORA73A--RF00100 | 3 | 40 | INCL_NON_REF_SPLICE |
| 34 | SNORA73A--RN7SK | 28 | 40 | INCL_NON_REF_SPLICE |
| 34 | SNORA73A--RN7SK | 22 | 40 | INCL_NON_REF_SPLICE |
| 34 | SNORA73A--RN7SK | 17 | 40 | INCL_NON_REF_SPLICE |
| 34 | SNORA73A--RN7SK | 12 | 40 | INCL_NON_REF_SPLICE |
| 34 | SNORA73A--RN7SK | 10 | 40 | INCL_NON_REF_SPLICE |
| 34 | SNORA73A--RN7SK | 9 | 40 | INCL_NON_REF_SPLICE |
| 34 | SNORA73A--RN7SK | 9 | 40 | INCL_NON_REF_SPLICE |
| 34 | SNORA73A--RN7SK | 8 | 40 | INCL_NON_REF_SPLICE |
| 34 | SNORA73A--RN7SK | 7 | 40 | INCL_NON_REF_SPLICE |
| 34 | SNORA73A--RN7SK | 5 | 40 | INCL_NON_REF_SPLICE |
| 34 | SNORA73A--RN7SK | 5 | 40 | INCL_NON_REF_SPLICE |
| 34 | SNORA73A--RN7SK | 3 | 40 | INCL_NON_REF_SPLICE |
| 34 | SNORA73A--RN7SK | 3 | 40 | INCL_NON_REF_SPLICE |
| 34 | SNORA73A--RN7SK | 3 | 40 | INCL_NON_REF_SPLICE |
| 34 | SNORA73A--RN7SK | 3 | 40 | INCL_NON_REF_SPLICE |
| 34 | SNORA73A--RN7SK | 3 | 40 | INCL_NON_REF_SPLICE |
| 34 | SNORA73A--RN7SK | 3 | 40 | INCL_NON_REF_SPLICE |
| 34 | SNORA73A--RNA5-8SN2 | 23 | 1 | INCL_NON_REF_SPLICE |
| 34 | SNORA73A--RNA5-8SN2 | 23 | 1 | INCL_NON_REF_SPLICE |
| 34 | SNORA73A--RNA5-8SN2 | 20 | 2 | INCL_NON_REF_SPLICE |
| 34 | SNORA73A--RNA5-8SN2 | 20 | 1 | INCL_NON_REF_SPLICE |
| 27 | SNORA73A--RNU1-1 | 11 | 0 | INCL_NON_REF_SPLICE |
| 27 | SNORA73A--RNU1-1 | 7 | 0 | INCL_NON_REF_SPLICE |
| 34 | SNORA73A--RPPH1 | 16 | 13 | INCL_NON_REF_SPLICE |
| 34 | SNORA73B--AL139099.4 | 12 | 41 | INCL_NON_REF_SPLICE |
| 34 | SNORA73B--AL139099.4 | 9 | 41 | INCL_NON_REF_SPLICE |
| 34 | SNORA73B--AL139099.4 | 8 | 41 | INCL_NON_REF_SPLICE |
| 34 | SNORA73B--AL139099.4 | 8 | 41 | INCL_NON_REF_SPLICE |
| 34 | SNORA73B--AL139099.4 | 6 | 41 | INCL_NON_REF_SPLICE |
| 34 | SNORA73B--AL139099.4 | 5 | 41 | INCL_NON_REF_SPLICE |
| 34 | SNORA73B--AL139099.4 | 5 | 41 | INCL_NON_REF_SPLICE |
| 34 | SNORA73B--AL139099.4 | 4 | 41 | INCL_NON_REF_SPLICE |
| 34 | SNORA73B--AL139099.4 | 4 | 41 | INCL_NON_REF_SPLICE |
| 34 | SNORA73B--AL139099.4 | 4 | 41 | INCL_NON_REF_SPLICE |
| 34 | SNORA73B--AL139099.4 | 3 | 41 | INCL_NON_REF_SPLICE |
| 34 | SNORA73B--AL139099.4 | 3 | 41 | INCL_NON_REF_SPLICE |
| 34 | SNORA73B--AL139099.4 | 3 | 41 | INCL_NON_REF_SPLICE |
| 34 | SNORA73B--AL139099.4 | 3 | 41 | INCL_NON_REF_SPLICE |
| 34 | SNORA73B--AL139099.4 | 3 | 41 | INCL_NON_REF_SPLICE |
| 34 | SNORA73B--AL139099.4 | 3 | 41 | INCL_NON_REF_SPLICE |
| 27 | SNORA73B--RF00003 | 13 | 0 | INCL_NON_REF_SPLICE |
| 34 | SNORA73B--RN7SL1 | 12 | 41 | INCL_NON_REF_SPLICE |
| 34 | SNORA73B--RN7SL1 | 9 | 41 | INCL_NON_REF_SPLICE |
| 34 | SNORA73B--RN7SL1 | 8 | 41 | INCL_NON_REF_SPLICE |
| 34 | SNORA73B--RN7SL1 | 8 | 41 | INCL_NON_REF_SPLICE |
| 34 | SNORA73B--RN7SL1 | 6 | 41 | INCL_NON_REF_SPLICE |
| 34 | SNORA73B--RN7SL1 | 5 | 41 | INCL_NON_REF_SPLICE |
| 34 | SNORA73B--RN7SL1 | 5 | 41 | INCL_NON_REF_SPLICE |
| 34 | SNORA73B--RN7SL1 | 4 | 41 | INCL_NON_REF_SPLICE |
| 34 | SNORA73B--RN7SL1 | 4 | 41 | INCL_NON_REF_SPLICE |
| 34 | SNORA73B--RN7SL1 | 4 | 41 | INCL_NON_REF_SPLICE |
| 34 | SNORA73B--RN7SL1 | 3 | 41 | INCL_NON_REF_SPLICE |
| 34 | SNORA73B--RN7SL1 | 3 | 41 | INCL_NON_REF_SPLICE |
| 34 | SNORA73B--RN7SL1 | 3 | 41 | INCL_NON_REF_SPLICE |
| 34 | SNORA73B--RN7SL1 | 3 | 41 | INCL_NON_REF_SPLICE |
| 34 | SNORA73B--RN7SL1 | 3 | 41 | INCL_NON_REF_SPLICE |
| 34 | SNORA73B--RN7SL1 | 3 | 41 | INCL_NON_REF_SPLICE |
| 49 | SNORA73B--RN7SL2 | 3 | 18 | INCL_NON_REF_SPLICE |
| 27 | SNORA73B--RNU4-1 | 9 | 0 | INCL_NON_REF_SPLICE |
| 52 | SPEF2--TPD52 | 4 | 1 | ONLY_REF_SPLICE |
| 52 | TCEB1--GGH | 5 | 0 | ONLY_REF_SPLICE |
| 52 | TCEB1--RPH3A | 5 | 0 | ONLY_REF_SPLICE |
| 44 | TERF2IP--UBB | 4 | 0 | ONLY_REF_SPLICE |
| 44 | TMCC2--AHSP | 4 | 0 | INCL_NON_REF_SPLICE |
| 55 | TNPO2--PRPS2 | 5 | 0 | INCL_NON_REF_SPLICE |
| 24 | TRIM58--OR2W3 | 6 | 0 | INCL_NON_REF_SPLICE |
| 52 | TSPAN31--CRY1 | 31 | 4 | INCL_NON_REF_SPLICE |
| 52 | UPK3BL--RN7SL5P | 3 | 2 | INCL_NON_REF_SPLICE |
| 47 | USP22--AL139099.4 | 5 | 0 | INCL_NON_REF_SPLICE |
| 47 | USP22--RN7SL1 | 5 | 0 | INCL_NON_REF_SPLICE |
| 32 | WAS--RMRP | 4 | 0 | INCL_NON_REF_SPLICE |
| 25 | ZNF292--PNRC1 | 5 | 1 | ONLY_REF_SPLICE |
| 32 | ZNF787--TOB2 | 4 | 0 | INCL_NON_REF_SPLICE |


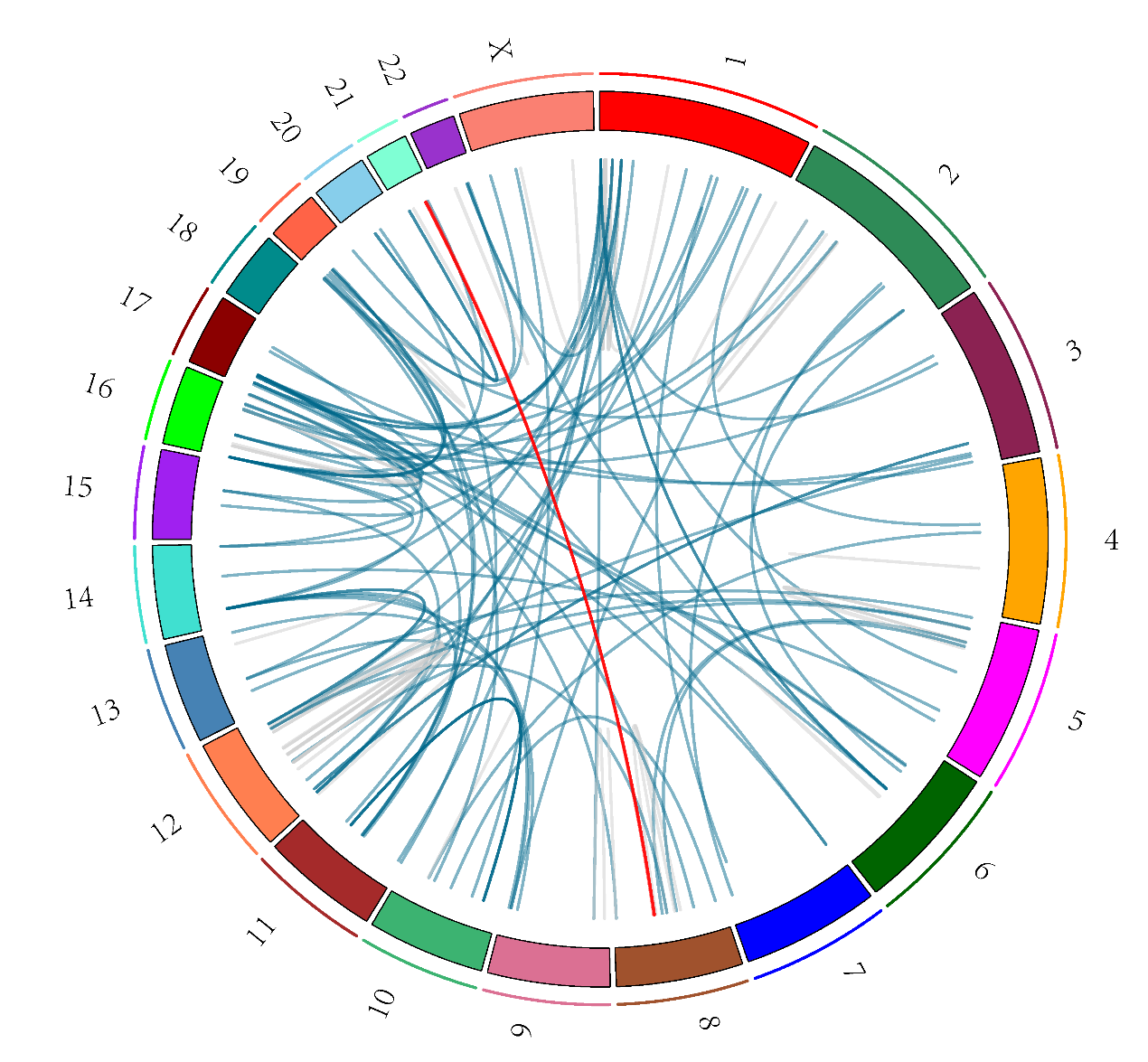


**Fig. S1**
